# Novel N-Substituted 3-Aryl-4-(diethoxyphosphoryl)azetidin-2-ones as Antibiotic Enhancers and Antiviral Agents in Search for a Successful Treatment of Complex Infections

**DOI:** 10.3390/ijms22158032

**Published:** 2021-07-27

**Authors:** Iwona E. Głowacka, Magdalena Grabkowska-Drużyc, Graciela Andrei, Dominique Schols, Robert Snoeck, Karolina Witek, Sabina Podlewska, Jadwiga Handzlik, Dorota G. Piotrowska

**Affiliations:** 1 Bioorganic Chemistry Laboratory, Faculty of Pharmacy, Medical University of Lodz, ul. Muszynskiego 1, 90-151 Lodz, Poland; iwona.glowacka@umed.lodz.pl (I.E.G.); magdalena.grabkowska-druzyc@umed.lodz.pl (M.G.-D.); 2 KU Leuven Department of Microbiology, Immunology and Transplantation, Rega Institute, Laboratory of Virology and Chemotherapy, Herestraat 49, Box 1030, B-3000 Leuven, Belgium; graciela.andrei@kuleuven.be (G.A.); dominique.schols@kuleuven.be (D.S.); robert.snoeck@kuleuven.be (R.S.); 3 Department of Technology and Biotechnology of Drugs, Faculty of Pharmacy, Jagiellonian University, Medical College, ul. Medyczna 9, 30-688 Krakow, Poland; karolina.witek@uj.edu.pl (K.W.); smusz@if-pan.krakow.pl (S.P.); j.handzlik@uj.edu.pl (J.H.); 4 Maj Institute of Pharmacology, Polish Academy of Sciences, Department of Medicinal Chemistry, ul. Smętna 12, 31-343 Krakow, Poland

**Keywords:** β-lactams, phosphonates, antiviral, cytostatic, MRSA, PBP2a, antibiotic adjuvant, molecular docking, molecular dynamics

## Abstract

A novel series of N-substituted *cis*- and *trans*-3-aryl-4-(diethoxyphosphoryl)azetidin-2-ones were synthesized by the Kinugasa reaction of N-methyl- or N-benzyl-(diethyoxyphosphoryl)nitrone and selected aryl alkynes. Stereochemistry of diastereoisomeric adducts was established based on vicinal H3–H4 coupling constants in azetidin-2-one ring. All the obtained azetidin-2-ones were evaluated for the antiviral activity against a broad range of DNA and RNA viruses. Azetidin-2-one *trans*-**11f** showed moderate inhibitory activity against human coronavirus (229E) with EC_50_ = 45 µM. The other isomer *cis*-**11f** was active against influenza A virus H1N1 subtype (EC_50_ = 12 µM by visual CPE score; EC_50_ = 8.3 µM by TMS score; MCC > 100 µM, CC_50_ = 39.9 µM). Several azetidin-2-ones **10** and **11** were tested for their cytostatic activity toward nine cancerous cell lines and several of them appeared slightly active for Capan-1, Hap1 and HCT-116 cells values of IC_50_ in the range 14.5–97.9 µM. Compound *trans*-**11f** was identified as adjuvant of oxacillin with significant ability to enhance the efficacy of this antibiotic toward the highly resistant *S. aureus* strain HEMSA 5. Docking and molecular dynamics simulations showed that enantiomer (3*R*,4*S*)-**11f** can be responsible for the promising activity due to the potency in displacing oxacillin at β-lactamase, thus protecting the antibiotic from undesirable biotransformation.

## 1. Introduction

**Figure 1:**
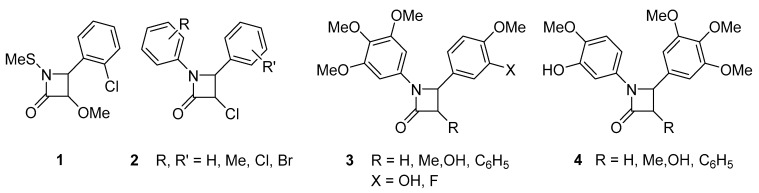
Example of azetidinones exhibiting anticancer activity.

The compounds containing azetidinone are of special importance both in chemistry and medicine. Since the discovery of penicillin, the application of azetidinone derivatives has been mainly associated with their antibacterial activity [1]. The family of azetidinone antibiotics (β-lactam antibiotics) includes penems, cephalosporins, monobactams and carbapenems, among others [2,3,4,5,6]. On the other hand, the azetidinone ring is a common structural motif of a vast number of compounds possessing a wide range of other biological properties, including antimalarial [7], antitubercular [8], anti-inflammatory [9], antifungal [10], antidepressant [11] and nootropic activity [11]. Azetidinone derivatives are also known as cholesterol absorption [12,13], human tryptase [14,15] and chymase inhibitors [15,16], as well as vasopressin V1a antagonists [17]. Anticancer [18] and antiviral [15,19] activities of compounds having azetidinone skeleton have been also recognized. Thus, 4-(2-chlorophenyl)-3-methoxy-1-(methylthio)azetidin-2-one **1** (Figure 1) has been found to inhibit proliferation and induce apoptosis in human solid cell lines, including breast and prostate [20]. Similarly, 1,4-diaryl-3-chloroazetidin-2-ones **2** have been recognized as potent agents for the treatment of human breast cancer [21]. Series of 1,4-diarylazetidinone with methoxyphenyl units have been obtained and their potency as inhibitors of various tumor types has been evaluated by Fuselier [22] and Meegan [23,24]. Among them, compounds **3** and **4** (Figure 1) exhibited antiproliferative activity against MCF-7 and MDA-MB-231 human breast carcinoma cells at nanomolar concentrations [23]. Moreover, the fluorinated analogues **3** exhibited significant anticancer activity against HT-29 colon cancer cells [25].

**Figure 2:**
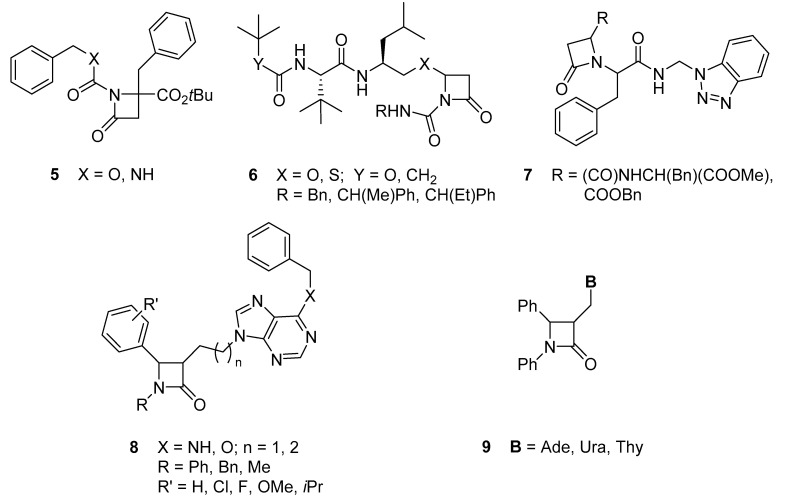
Example of azetidinones exhibiting antiviral activity.

Compounds with antiviral properties could be found among those containing an azetidinone unit in their structures. For example, non-nucleoside analogues of azetidinone **5** (Figure 2) exhibited activity towards human cytomegalovirus (HCMV) [26,27]. Peptide linked monocyclic azetidinones **6** showing an inhibitory activity against human cytomegalovirus protease have been also synthesized by Dézeil [28]. Similarly, Sperka and co-workers discovered compounds **7** as inhibitors of HIV-1 protease [29]. Moreover, D’hooghe et al. obtained compounds **8** by introduction of a modified purine nucleobase into an azetidinone ring [19]. Purine β-lactam hybrids showed moderate to good activities against different viruses, i.e., human respiratory syncytial virus (RSV), chikungunya virus (ChikV), HCMV, hepatitis B virus (HBV) and coxsackie B virus (CoxV) [19]. The results of antiviral and cytotoxic activity studies on compounds **9** were so encouraging that identification of several new lead structures among this type of compounds was possible [19,30,31].

The search for effective antiviral drugs, among newly designed compounds as well as already known ones, became even more challenging in the eyes of the coronavirus pandemic (COVID-19). In fact, symptomatic therapy is appropriate in the treatment of milder illnesses, and antimicrobial drugs are often necessary when bacterial complications occur. Especially, the methicillin resistant *Staphylococcus aureus* (MRSA) is a Gram-positive member of the most problematic bacteria in clinical treatment, so-called ESKAPE [32]. Various clinical isolates of MRSA are multidrug resistant (MDR), i.e., resistant to antibiotics representing different classes, including β-lactams, macrolides, tetracyclines, etc. Taking into account the structural analogy between β-lactam antibiotics and functionalized derivatives of azetidinones, the latter ones provide some hope in search for effective agents against MRSA, either as new antibacterials less susceptible to MDR mechanisms or as antibiotic “adjuvants” that, being bioisosteres of antibiotics, may be mistakenly recognized as substrates of various bacterial MDR proteins. Thus, further extensive pharmacomodulations among azetidinones are an important challenge for current medicinal chemistry in order to search for innovative therapeutic solutions in the treatment of complex infectious diseases.

**Scheme 1:**
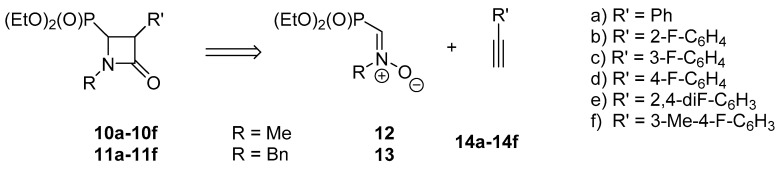
Retrosynthesis of azetidon-2-ones **10** and **11**.

Recently, we communicated a convenient method for the synthesis of 4-phosphonylated azetidin-2-ones substitued with various aryl groups at C3 [33]. The proposed methodology relied on the application of Kinugasa reaction of *N*-methyl *C*-phosphonylated nitrone with terminal acetylenes. In this paper, a full account of studies on preparation of the series of N-substitued 3-aryl-4-(diethoxyphosphonyl)azetidion-2-ones of the general formulae **10** and **11** (Scheme 1) is presented together with the results of their antiviral, cytostatic and antimicrobial/antibiotic adjuvant properties.

## 2. Results and Discussion

### 2.1. Chemistry

**Scheme 2:**
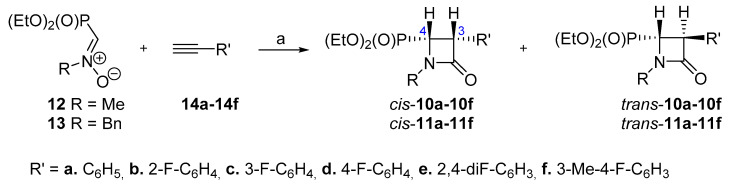
Reagents and conditions: a. Procedure A; (i) **14** (3eq), CuI (3 eq), NEt_3_ (3 eq), MeCN, 0 °C, 0.5 h; (ii) nitrone **12** or **13**, r.t., 72 h; procedure B: (i) **14** (1.5 eq), CuI (0.1 eq), NEt_3_ (0.05 eq), DMAP (0.05 eq), MeCN, 0 °C, 0.5 h; (ii) nitrone **12** or **13**, MW 30–40 °C, 4 h.

As reported earlier, standard conditions were applied for the Kinugasa reaction of nitrone **12** to terminal arylacetylenes **14a-c** and **14e** [33], namely 3 equivalents of CuI and triethylamine to generate respective copper(I)arylacetylide. After the optimization of the reaction conditions [33], cycloadditions of the nitrone **12** to arylalkynes **14** were carried out using 1.5 equivalent of the respective arylalkyne in the presence of catalytic amounts of CuI, triethylamine and DMAP under microwave irradiation, which significantly shortened the time required for full conversion of the nitrone (4 h vs. 72 h) (Scheme 2, Table 1). For the purpose of this project, the set of alkynes applied in this reaction was expanded by arylacetylene **14d** and **14f** (Scheme 2, Table 1, entry d and f). Moreover, *N*-benzyl-*C*-(diethoxyphosphoryl)nitrone **13** was also used in Kinugasa reaction to fill the library of the azetidinones for biological studies (Scheme 2, Table 2).

The ratios of diastereoisomers were calculated from ^31^P and ^1^H NMR spectra of crude reaction mixtures. Diastereoisomeric azetidinones were successfully separated by column chromatography and both pure isomers (except for *cis*-**10c** and *cis*-**11c**) were isolated in almost all cases, namely *cis*-**10a**, *cis*-**10b**, *cis*-**10d-f** and *trans*-**10a-f** as well as *cis*-**11a**, *cis*-**11b**, *cis*-**11d-f** and *trans*-**11a-f** (Table 1 and Table 2).

The correlation between the *cis* and *trans* configuration of 3,4-disubstituted azetidinones and the observed values of vicinal H3–H4 coupling constants has been well recognized [34,35,36]. In the case of compounds *cis*-**10** and *cis*-**11,** as well as *trans*-**10** and *trans*-**11** for configurational assignments, the analogous correlation was also applied. Thus, in the ^1^H NMR spectra of *cis*-**10** and *cis*-**11**, vicinal couplings for H3–H4 protons in the 5.5–6.9 Hz range were observed, whereas in the series of *trans*-**10** and *trans*-**11**, significantly lower coupling values were noticed for H3–H4 protons (^3^*J*_H3–H4_ = 2.4–2.9 Hz). Furthermore, the one-bond phosphorus-carbon coupling constant values (^1^*J*_C–P_) also appeared to be useful since diagnostic differences in coupling constants were found in the series of all 3-aryl-4-(diethoxyphosphoryl)azetidin-2-ones *cis*-**10** and *cis*-**11** in comparison to analogous *trans*-**10** and *trans*-**11**. The observed values of couplings for all *cis*-isomers were higher (^1^*J*_C–P_ = 170.6–173.0 Hz) when compared to the coupling constants for the other *trans*-configured diastereoisomers (^1^*J*_C–P_ = 164.6–166.6 Hz).

### 2.2. Pharmacology

#### 2.2.1. Antiviral Activity

All obtained azetidinones *cis*-**10** and *trans*-**10,** as well as *cis*-**11** and *trans*-**11,** were evaluated for inhibitory activity against a wide variety of DNA and RNA viruses, using the following cell-based assays: (a) human embryonic lung (HEL) cells: herpes simplex virus-1 (KOS), herpes simplex virus-2 (G), thymidine kinase deficient (acyclovir resistant) herpes simplex virus-1 (TK^−^ KOS ACV^r^), vaccinia virus, adenovirus-2, human coronavirus (229E), cytomegalovirus (AD-169 strain and Davis strain), varicella-zoster virus (TK^+^ VZV Oka strain and TK^−^ VZV 07-1 strain); (b) HeLa cell cultures: vesicular stomatitis virus, Coxsackie virus B4 and respiratory syncytial virus (RSV); (c) Vero cell cultures: parainfluenza-3 virus, reovirus-1, Sindbis virus, Coxsackie virus B4, Punta Toro virus, yellow fever virus and (d) MDCK cell cultures: influenza A virus (H1N1 and H3N2 subtypes) and influenza B virus. Ganciclovir, cidofovir, acyclovir, brivudin, zalcitabine, zanamivir, alovudine, amantadine, rimantadine, ribavirin, dextran sulfate (molecular weight 10,000, DS-10,000), mycophenolic acid, and Urtica dioica agglutinin (UDA) were used as reference compounds. The antiviral activity was expressed as the EC_50_: the compound concentration required to reduce virus plaque formation (VZV) by 50% or to reduce virus-induced cytopathogenicity by 50% (other viruses). The cytotoxicity of the tested compounds toward the uninfected HEL, HeLa, Vero and MDCK cells was defined as the minimum cytotoxic concentration (MCC) that causes a microscopically detectable alteration of normal cell morphology. The 50% cytotoxic concentration (CC_50_), causing a 50% decrease in cell viability was determined using a colorimetric 3-(4,5-dimethylthiazol-2-yl)-5-(3-carboxymethoxy-phenyl)-2-(4-sulfophenyl)-2H-tetrazolium (MTS) assay system.

**Figure 3:**
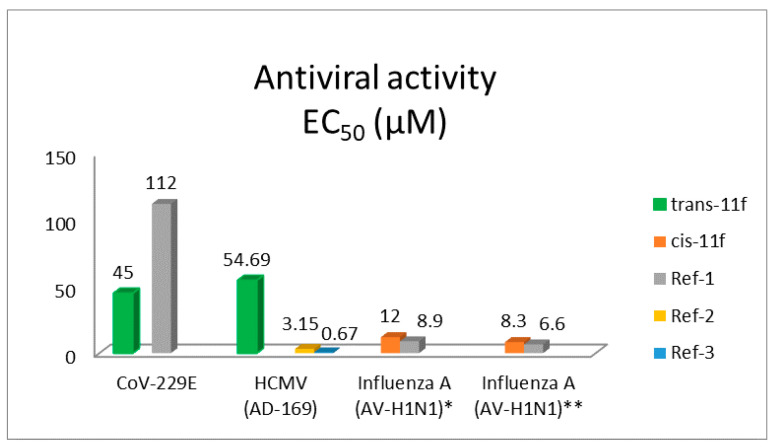
Antiviral activity found for the stereoisomers of 3-methyl-4-fluorophenyl derivatives of azetidinone *trans*-**11f** and *cis-***11f** in comparison with reference antiviral agents: ribavirin (Ref-1), ganciclovir (Ref-2), cidofovir (Ref-3). The ability to inhibit viral replication expressed with EC_50_ value (µM). * EC_50_ by visual CPE score; ** EC_50_ by TMS score.

Among all tested compounds, the stereoisomers having 3-methyl-4-fluorophenyl group at C3 in azetidinone ring **(11f)** showed modest antiviral activity (Figure 3). The isomeric azetidinone *trans*-**11f** was able to inhibit the replication of human coronavirus (229E) (EC_50_ = 45 µM) and its activity was almost 2.5-fold higher than that of a reference drug ribavirin (EC_50_ = 112 µM). Moreover, activity of azetidinone *trans*-**11f** toward cytomegalovirus AD-169 strain (EC_50_ = 54.69 µM) was also noticed, although it was less active than ganciclovir and cidofovir used as reference drugs. At the same time the compound *trans*-**11f** did not affect normal cell morphology. On the other hand, the isomer *cis*-**11f** appeared to be active against influenza A virus H1N1 subtype (EC_50_ = 12 µM by visual CPE score; EC_50_ = 8.3 µM by TMS score; MCC > 100 µM, CC_50_ = 39.9 µM) in Madin Darby canine kidney cells (MDCK) and its potency was comparable to ribavirin used as a reference compound (EC_50_ = 8.9 µM by visual CPE score; EC_50_ = 6.6 µM by TMS score; MCC > 100 µM, CC_50_ ≥ 100 µM), but much lower than that of zanamivir, amantadine and rimantadine. None of the compounds described herein were active against the other tested DNA and RNA viruses and none was cytotoxic toward used cell lines at concentrations up to 100 µM.

In regard to the structure-activity relationship, the introduction of benzyl instead of methyl group at nitrogen together with 3-methyl-4-fluorophenyl group at C3 in azetidinone ring seems to be crucial for the observed antiviral activity. Surprisingly, the presence of a monosubstituted phenyl function at C3, regardless of the position of the substituent, is insufficient to maintain the activity. Moreover, the stereochemistry of the azetidinone ring appeared to be important for the activity and selectivity of **11f** toward the targeted viruses, i.e., *trans*-isomer displayed selective action for coronavirus CoV-229 and cytomegalovirus HMCV (AD-169), while *cis*–for influenza A (H1N1). An additional advantage of the 3-methyl-4-fluorophenyl derivatives of azetidinone is their safety (no cytotoxic effects) for the whole tested panel of uninfected cell lines.

Although these initial results are not sufficient to recognize and explore likely molecular mechanisms of the antiviral activities of **11f** isomers, they mark the 3-methyl-4-fluorophenyl scaffold as an important pharmacophore feature worth to be considered in further search for antiviral drugs among the azetidine derivatives.

#### 2.2.2. Antibacterial Action

All synthesized azetidinones *cis*-**10** and *trans*-**10,** as well as *cis*-**11** and *trans*-**11,** were screened for their antibacterial activity against the Gram-positive *S. aureus,* including reference strain ATCC 25,923 and the multidrug resistant clinical isolate MRSA HEMSA 5. The tested compounds did not inhibit the growth of either *S. aureus* strains at concentrations up to 2 mM. Thus, their antibacterial activity can be considered negligible.

#### 2.2.3. Antibiotics Enhancer Properties

Since the lack of direct antibacterial activity of the obtained azetidinones was observed, all isomers *trans* and *cis* were investigated on their “adjuvant” properties, i.e., an ability to enhance the effectiveness of antibiotics against *S. aureus* strains. Thus, the compounds were tested in combination with the known β-lactam antibiotic, oxacillin, in the microdilution assays. The ability of the tested azetidinones to reduce minimum inhibitory concentration (MIC) of oxacillin against both, the referenced and the resistant *S. aureus* strains, was assessed. In the absence of the tested compounds, oxacillin showed MIC value of 0.5 µg/mL against the ATCC 25,923 strain, while 512 µg/mL for the MRSA HEMSA 5, the strain highly resistant to this antibiotic. The azetidinones were tested in the 0.5 mM, i.e., the inactive concentration of each compound (≤¼ MIC) against both strains. Results are shown in Table 3.

Among all tested compounds, the strong chemosensitizing effect was demonstrated by compound *trans*-**11f**, which reduced the MIC of oxacillin 16-fold against MRSA HEMSA 5 (oxacillin MIC in the presence of the tested compound reduced to 32 µg/mL). Other azetidinones did not improve the susceptibility of MRSA to oxacillin in a significant manner. On the other hand, none of the tested compounds had an impact on the oxacillin activity toward the *S. aureus* ATCC 25,923 strain, and an even higher concentration of the antibiotic was necessary to inhibit growth of the bacteria when the tested compound was added.

#### 2.2.4. Cytostatic Activity

The 50% cytostatic inhibitory concentration (IC_50_) causing a 50% decrease in cell proliferation was determined for all obtained compounds toward 9 cancerous cell lines, i.e.,: Capan-1 (pancreatic adenocarcinoma), Hap1 (chronic myeloid leukemia), HCT-116 (colorectal carcinoma), NCI-H460 (lung carcinoma), DND-41 (acute lymphoblastic leukemia), HL-60 (acute myeloid leukemia), K-562 (chronic myeloid leukemia), MM.1S (multiple myeloma), Z-138 non-Hodgkin lymphoma), as well as normal retina (non-cancerous) cells (hTERT RPE-1). Docetaxel, etoposide and stauroporine were used as the reference compounds. Results are shown in Table 4.

Among all tested compounds, none were active against DND-41, HL-60, K-562, MM.1S and Z-138 cancer cells at the concentrations up to 100 µM, except the compound *trans*-**11f** which showed low activity against DND-41 cells (IC_50_ = 65.8 µM). Most of the compounds described herein were also not toxic or showed negligible toxicity to non-cancerous retina cells (hERT RPE-1), except *trans*-**11c**, *trans*-**11d**, *trans*-**11e** and *trans*-**11f** which exhibited noticeable antiproliferation activities (IC_50_ = 45.9, 73.0, 25.6 and 33.5, µM, respectively) (Table 4). All of the tested azetidinones **10** and **11**, except *cis*-**10d** (Ar = 4-F-C_6_H_4_), exhibited moderate activity against pancreatic adenocarcinoma cells (Capan-1) (IC_50_ from 19.6 to 95.2 µM), and among them *trans*-**10c** (Ar = 3-F-C_6_H_4_) was the most active with IC_50_ value of 19.6 µM, but the inhibitory concentration was much lower than that of the reference drugs (Table 4). On the other hand, the highest inhibitory effect against the proliferation of chronic myeloid leukemia (Hap1) was observed for compounds *cis*-**10b** (Ar = 2-F-C_6_H_4_) (IC_50_ = 14.5 µM), however in most cases the activity values of the tested compounds toward chronic myeloid leukemia (Hap1) were slightly lower than these observed for the same series of compounds toward Capan-1 cells. Compound *trans*-**11e** having 2,4-difuorophenyl moiety at C3 in the azetidinone ring appeared to be the most active toward lung carcinoma (NCI-H460) (IC_50_ = 24.4 µM) but unfortunately no selectivity was observed when compared to normal retina (non-cancerous) cells (hTERT RPE-1) (IC_50_ = 25.6 µM). Interestingly, N-benzylated azetidinones **11** exhibited moderate activity toward colorectal carcinoma cells (HCT-116) (IC_50_ = 35.3 to 87.9 µM), whereas most of analogous N-methyl azetidinones **10** were inactive at the concentration up to 100 µM, except for *trans*-**10b** and *cis*-**10d** (IC_50_ = 44.1 and 86.5 µM, respectively). In the case of the other cancerous cell lines, no significant correlation between structure and the observed activity was noticed.

### 2.3. Computer-Aided Insight into Antibiotic “Adjuvant” Action

**Figure 4:**
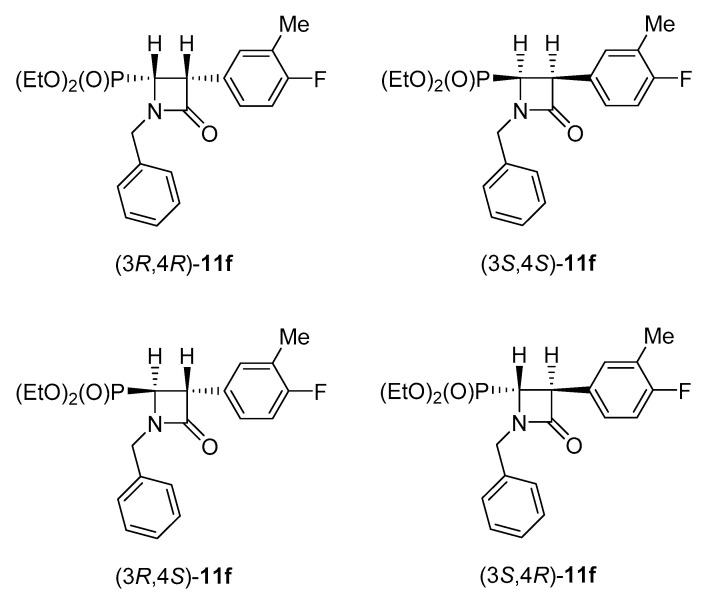
Four stereoisomers of **11f** explored in silico.

The semi-synthetic β-lactam antibiotic oxacillin used in this study is known as antistaphylococcal penicillin, which is resistant to hydrolysis by most staphylococcal β-lactamases [36]. Its bactericidal activity results from the inhibition of bacterial cell wall biosynthesis via interaction with penicillin binding proteins (PBPs) [37]. The resistance to oxacillin primarily stems from the acquisition of the *mecA* gene encoding PBP2a with lower affinity to β-lactams [38] but various other mechanisms are also possible. Taking into account both, the high structural similarity of investigated 3-aryl-4-(diethoxyphosphoryl)azetidin-2-ones to β-lactam antibiotics and unknown modifications of β-lactamase in the tested XDS strain HEMSA-5, a competitive displacement of oxacillin by *trans*-**11f** at β-lactamase seems to be a probable mechanism as well. Therefore, we decided to estimate either β-lactamase or PBP2a as possible targets involved into the oxacillin-enhancing action of *trans*-**11f**. In this context, advanced molecular modelling studies for four possible stereoisomers of **11f**, namely (3*R*,4*R*)-**11f**, (3*S*,4*S*)-**11f**, (3*R*,4*S*)-**11f** and (3*S*,4*R*)-**11f** (Figure 4), and oxacillin have been performed.

#### 2.3.1. Influence on β-Lactamase

**Figure 5:**
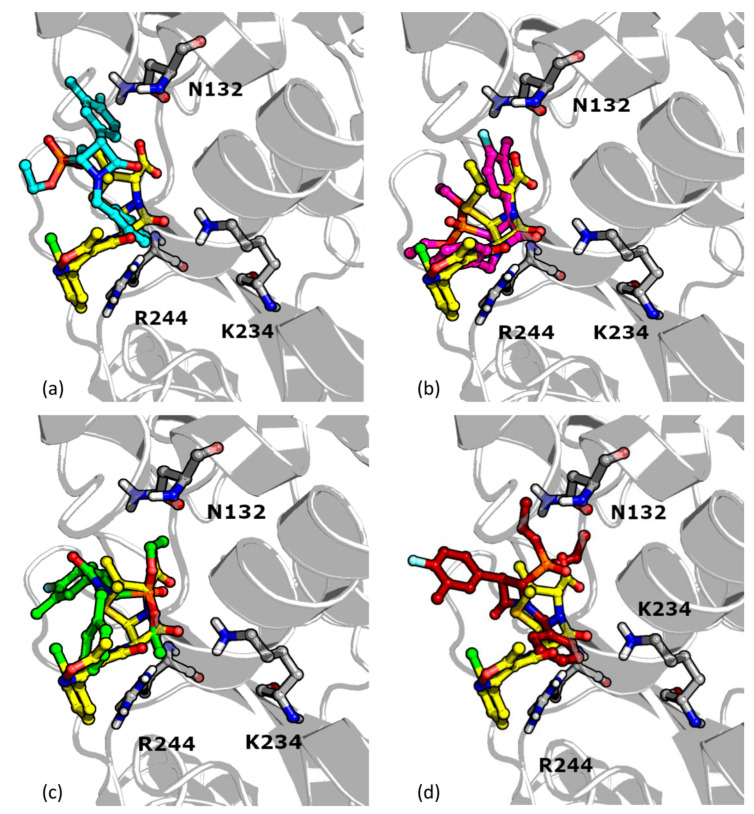
Docking results of compound **11f** and oxacillin to β-lactamase crystal structure: (**a**) (3*R*,4*R*)-**11f**, (**b**) (3*S*,4*S*)-**11f**, (**c**) (3*S*,4*R*)-**11f**, (**d**) (3*R*,4*S*)-**11f**; oxacillin is always depicted in yellow.

Due to the structural resemblance of the newly synthesized compounds to oxacillin, the possibility of them being the potential β-lactamase substrate was examined. Four stereoisomers of compound **11f** (Figure 4) were tested in docking and molecular dynamics (MD) simulations with β-lactamase from *Staphylococcus aureus*. For reference, oxacillin was also modelled in the same conditions. The structure of PC1 β-lactamase was used, which is the class A β-lactamase (class D β-lactamases are supposed to hydrolyze oxacillin; however, their crystal structures for *Staphylococcus aureus* are not available). The docking results of *cis*-**11f** (i.e., (3*R*,4*R*)-**11f** and (3*S*,4*S*)-**11f**) and *trans*-**11f** (i.e., (3*R*,4*S*)-**11f** and (3*S*,4*R*)-**11f**) are presented in Figure 5. Poses of all analyzed compounds are overlaid with the oxacillin orientation in the binding site.

**Figure 6:**
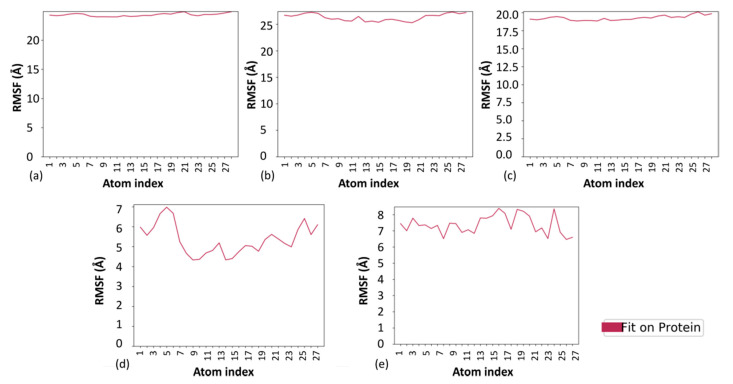
RMSF of modeled compounds during MD simulations with β-lactamase crystal structure (**a**) (3*R*,4*R*)-**11f**, (**b**) (3*S*,4*S*)-**11f**, (**c**) (3*S*,4*R*)-**11f**, (**d**) (3*R*,4*S*)-**11f**, (**e**) oxacillin.

**Figure 7:**
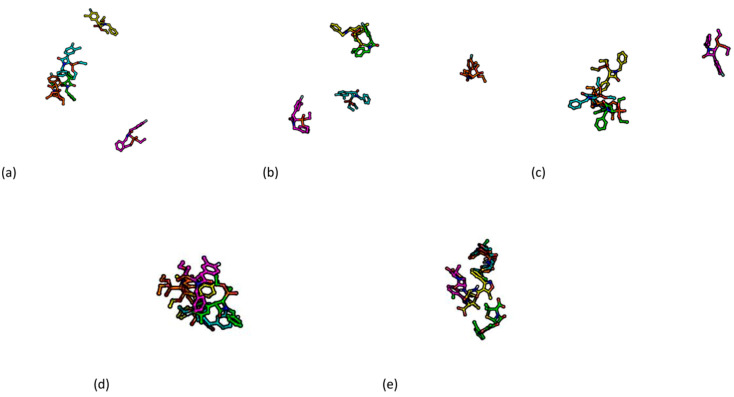
Selected frames from MD simulations for (**a**) (3*R*,4*R*)-**11f**, (**b**) (3*S*,4*S*)-**11f**, (**c**) (3*S*,4*R*)-**11f**, (**d**) (3*R*,4*S*)-**11f**, (**e**) oxacillin with β-lactamase; frame 1–green, 250–yellow, 500–magenta, 750–cyan, 1000–orange.

The analysis of the initial compound positions in docking indicates that most similar pose to this of oxacillin is obtained by (3*S*,4*S*)-**11f**. However, MD simulations showed that the docking poses were not very stable during MD and that the compound conformations were varying in the subsequent frames. The MD simulations revealed the possible mechanism of restoring oxacillin activity by *trans*-**11f**. One of its enantiomers, (3*R*,4*S*)-**11f**, is the only compound which (similarly to oxacillin) did not leave the β-lactamase active site and remains in the relatively similar position during the whole simulation (Figure 6 and Figure 7). On the other hand, all other compounds diffused away from their positions obtained in docking and they are unlikely to be the substrates for the β-lactamase.

These relationships are visible in Figure 6**,** which presents the Ligand Root Mean Square Fluctuation (L-RMSF). With the use of this parameter, changes in the ligand atom positions can be characterized and quantitatively measured. It is visible that for (3*R*,4*R*)-**11f**, (3*S*,4*S*)-**11f**, and (3*S*,4*R*)-**11f**, RMSF adopted high values: over 20 Å for *cis*-**11f** (both (3*R*,4*R*)-**11f** and (3*S*,4*S*)-**11f**) and close to 20 Å for (3*S*,4*R*)-**11f**. On the other hand, both (3*R*,4*S*)-**11f** and oxacillin remained in relatively similar positions during the whole MD, as their RMSF values did not exceed 10 Å.

The quantitative data were also in line with the qualitative analysis (Figure 7), where only (3*R*,4*S*)-**11f** and oxacillin remained in the same region during the whole MD simulation and did not diffuse away from the binding site. On the other hand, all the other compounds were not strongly fitted to β-lactamase and spent some simulation time away from the protein.

Due to the relatively stable position of (3*R*,4*S*)-**11f** in the β-lactamase active site during MD and its structural resemblance to oxacillin, we suggest that (3*R*,4*S*)-**11f** restores the oxacillin activity in MRSA via being a substrate for β-lactamase, which transforms (3*R*,4*S*)-**11f** instead of oxacillin, and thus oxacillin can play its antibacterial role in the unchanged form.

#### 2.3.2. Interactions with PBP2a

**Figure 8:**
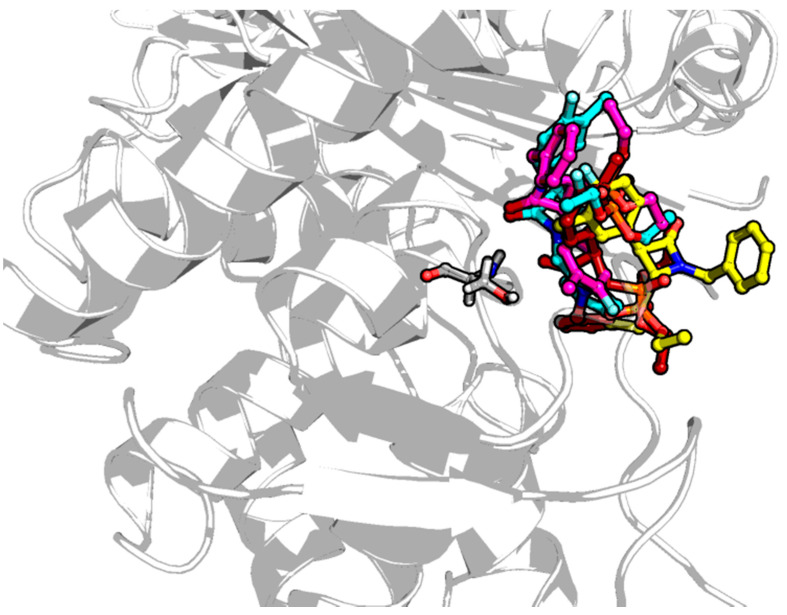
Docking results of various isomers of compound **11f** to the PBP2a binding site (S403 residue is indicated by sticks): (3*R*,4*R*)-**11f**–cyan, (3*S*,4*S*)-**11f**–magenta, (3*S*,4*R*)-**11f**–yellow, (3*R*,4*S*)-**11f**–red.

In the next step, the hypothesis of PBP2a being a potential target was examined. We verified the scheme of interactions of different isomers of compound **11f** with the PBP2a protein, the alternative penicillin binding protein with the reduced affinity for β-lactam antibiotics. Analogously, docking and MD simulations were applied, as for studies with β-lactamase. The docking results are shown in Figure 8.

The docking poses to the PBP2a active site indicate a very similar orientation of both *cis*-**11f** enantiomers, and (3*R*,4*S*)-**11f** was also docked similarly. On the other hand, (3*S*,4*R*)-**11f** adopted a significantly different pose, which is, however, the furthest away from the active-site serine (S403).

**Figure 9:**
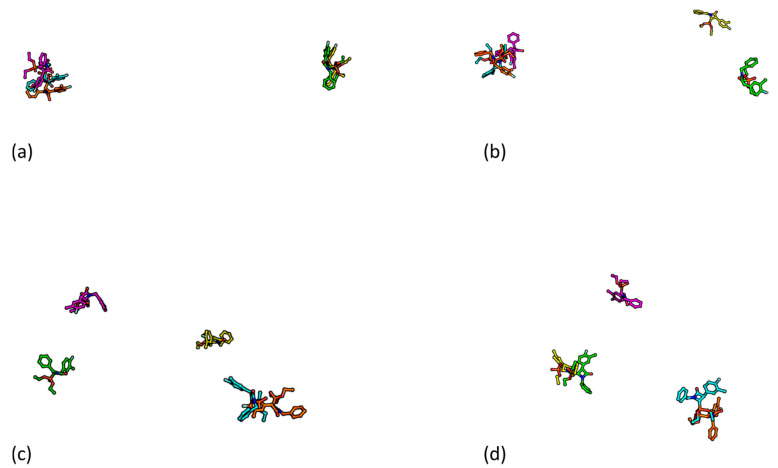
Docking selected frames from MD simulations for (**a**) (3*R*,4*R*)-**11f**, (**b**) 3*S*,4*S*)-**11f**, (**c**) (3*S*,4*R*)-**11f**, (**d**) (3*R*,4*S*)-**11f** with PBP2a; frame 1–green, 250–yellow, 500–magenta, 750–cyan, 1000–orange.

In order to validate the docking poses and examine their stability in the binding pocket, MD simulations were carried out (Figure 9). The analysis of compound poses obtained at different time points of the simulation indicates that their poses were very unstable during MD. If not immediately (as (3*S*,4*R*)-**11f**), all compounds left the active site of PBP2a after less than 200 ns of simulation. These results indicate that the interaction of PBP2a with examined compounds is very unstable and suggest rather low probability that any stereoisomers of **11f** is the PBP2a agent.

Thus, the most probable mechanism of the “adjuvant” action of *trans*-**11f** seems to be mediated by β-lactamase, in which the enantiomer (3*R*,4*S*)-**11f** is probably the responsible component due to predominant ability of β-lactamase substrate.

## 3. Materials and Methods

### 3.1. Chemistry

General information–^1^H NMR spectra were taken in CDCl_3_ on a Bruker Avance III (600 MHz); chemical shifts δ are given in ppm with respect to TMS and coupling constants *J* in Hz. ^13^C NMR and ^31^P NMR spectra were recorded in a ^1^H-decoupled mode for CDCl_3_ solutions on the Bruker Avance III (600 MHz) spectrometer at 151 and 243 MHz, respectively. IR spectral data were measured on a Bruker Alpha-T FT-IR spectrometer. Melting points were determined on a Boetius apparatus and are uncorrected. Elemental analyses were performed by the Microanalytical Laboratory of the Faculty of Pharmacy (Medical University of Lodz) on a Perkin Elmer PE 2400 CHNS analyzer and their results were found to be in good agreement (±0.3%) with the theoretical values.

The following adsorbents were used: column chromatography, Merck silica gel 60 (70–230 mesh), analytical TLC, Merck TLC plastic sheets silica gel 60 F_254_. TLC plates were developed in chloroform-methanol solvent systems. Visualization of spots was effected with iodine vapours. All solvents were purified by methods described in the literature. The nitrones **12** and **13** were obtained according to the literature procedure [39,40]. The purity of the samples of all synthesised compounds **10** and **11** used for biological studies was established as ≥99.99%.

^1^H, ^13^C and ^31^P NMR spectra of all new synthesized compounds are provided in Supplementary Materials.

### 3.2. General Procedures for the Synthesis of Azetidine-2-Ones cis-10/trans-10 and cis-11/trans-11

#### 3.2.1. General Procedure A

A solution of alkyne **14** (3.0 mmol) in MeCN (1 mL) was cooled to 0 °C under argon atmosphere and CuI (3 mmol) was added, followed by Et_3_N (3 mmol). After 30 min the temperature was allowed to reach 25 °C, the respective nitrone **12** or **13** (1 mmol) in MeCN (1 mL) was added, and the reaction mixture was stirred for 72 h. Subsequently, the reaction mixture was diluted with MeCN and the suspension was filtered through the layer of Celite. The solution was concentrated and the crude product was purified on a silica gel column with chloroform:methanol (100:1, 50:1, *v/v*) and in some cases also by high-performance liquid chromatography (HPLC) using a X Bridge Prep, C18, 5 µm, OBD (Optimum Bed Density), 19 × 100 mm column and methanol:water mixture (62:38, 60:40, 55:45, *v*/*v*) as eluent.

#### 3.2.2. General Procedure B

A solution of alkyne **14** (1.5 mmol) in MeCN (1 mL) was cooled to 0 °C under argon atmosphere and CuI (0.1 mmol) was added, followed by Et_3_N (0.05 mmol) and DMAP (0.05 mmol). After 30 min the temperature was allowed to reach 25 °C, the respective nitrone **12** or **13** (1 mmol) in MeCN (1 mL) was added, and the reaction mixture was irradiated in the Plazmatronika RM800 microwave reactor at 30–40 °C for 4 h. Subsequently, the reaction mixture was diluted with MeCN, and the suspension was filtered through the layer of Celite. The solution was concentrated and the crude product was purified on a silica gel column with chloroform:methanol (100:1, 50:1) and in some cases also by high-performance liquid chromatography (HPLC) using a X Bridge Prep, C18, 5 µm, OBD (Optimum Bed Density), 19 × 100 mm column and methanol:water mixture (62:38, 60:40, 55:45, *v*/*v*) as eluent.

#### 3.2.3. cis-N-methyl-3-phenyl-4-(diethoxyphosphoryl)azetidin-2-one (cis-**10a**)

Colorless oil. IR (film, cm^−1^): ν = 3485, 2983, 2931, 1757, 1236, 1025, 752, 699. ^1^H NMR (600 MHz, CDCl_3_): δ = 7.35–7.30 (m, 2H), 7.26–7.23 (m, 2H), 7.21–7.15 (m, 1H), 4.72 (dd, ^2^*J*_(HCP)_ = 7.7, ^3^*J*_(HCCH)_ = 5.9 Hz, 1H, HC4), 3.98 (dd, ^3^*J*_(HCCP)_ = 5.9 Hz, ^3^*J*_(HCCH)_ = 5.9 Hz, 1H, HC3), 3.82–3.74 (m, 1H, CH_2_OP), 3.74–3.68 (m, 1H, CH_2_OP), 3.68–3.60 (m, 1H, CH_2_OP), 3.56–3.48 (m, 1H, CH_2_OP), 2.93 (s, 3H, CH_3_), 1.18 (t, ^3^*J*_(HCCH)_ = 7.1 Hz, 3H, C*H*_3_CH_2_OP), 1.13 (t, ^3^*J*_(HCCH)_ = 7.1 Hz, 3H, C*H*_3_CH_2_OP). ^13^C NMR (151 MHz, CDCl_3_): δ = 167.63 (d, *J* = 9.0 Hz, C=O), 131.91 (d, *J* = 2.8 Hz), 129.50, 128.08, 127.86, 62.15 (d, ^2^*J*_(COP)_ = 7.0 Hz, CH_2_OP), 61.83 (d, ^2^*J*_(COP)_ = 6.8 Hz, CH_2_OP), 57.56 (d, ^2^*J*_(CCP)_ = 1.7 Hz, C3), 54.98 (d, ^1^*J*_(CP)_ = 172.3 Hz, C4), 28.40 (CH_3_), 16.37 (d, ^3^*J*_(CCOP)_ = 6.0 Hz, *C*H_3_CH_2_OP), 16.29 (d, ^3^*J*_(CCOP)_ = 6.0 Hz, *C*H_3_CH_2_OP). ^31^P NMR (243 MHz, CDCl_3_): δ = 19.08. Anal. Calcd for C_14_H_20_NO_4_P: C, 56.56; H, 6.78; N, 4.71. Found: C, 56.29; H, 6.76; N, 4.87.

#### 3.2.4. trans-N-methyl-3-phenyl-4-(diethoxyphosphoryl)azetidin-2-one (trans-**10a**)

Colorless oil. IR (film, cm^−1^): ν = 3450, 2979, 2895, 1761, 1239, 1021, 790, 696. ^1^H NMR (300 MHz, CDCl_3_): δ = 7.40–7.28 (m, 5H), 4.54 (dd, ^2^*J*_(HCP)_ = 8.9, ^3^*J*_(HCCH)_ = 2.6 Hz, 1H, HC4), 4.30–4.18 (m, 4H, 2 × CH_2_OP), 3.67 (dd, ^3^*J*_(HCCP)_ = 9.1, ^3^*J*_(HCCH)_ = 2.6 Hz, 1H, HC3), 3.01 (s, 3H, CH_3_), 1.38 (t, ^3^*J*_(HCCH)_ = 7.2 Hz, 3H, C*H*_3_CH_2_OP), 1.37 (t, ^3^*J*_(HCCH)_ = 3H, C*H*_3_CH_2_OP). ^13^C NMR (75.5 MHz, CDCl_3_): δ = 167.57 (d, *J* = 12.8 Hz, C=O), 134.20 (d, *J* = 2.6 Hz), 128.98, 127.94, 127.30, 63.25 (d, ^2^*J*_(COP)_ = 6.7 Hz, CH_2_OP), 62.84 (d, ^2^*J*_(COP)_ = 7.0 Hz, CH_2_OP), 57.85 (d, ^2^*J*_(CCP)_ = 2.5 Hz, C3), 56.36 (d, ^1^*J*_(CP)_ = 164.6 Hz, C4), 28.77 (CH_3_), 16.94 (d, ^3^*J*_(CCOP)_ = 5.3 Hz, *C*H_3_CH_2_OP), 16.87 (d, ^3^*J*_(CCOP)_ = 5.6 Hz, *C*H_3_CH_2_OP). 31P NMR (243 MHz, CDCl_3_): 20.52. Anal. Calcd for C_14_H_20_NO_4_P: C, 56.56; H, 6.78; N, 4.71. Found: C, 56.42; H, 6.70; N, 4.73.

#### 3.2.5. cis-N-methyl-3-(2-fluorophenyl)-4-(diethoxyphosphoryl)azetidin-2-one (cis-**10b**)

Yellowish oil. IR (film, cm^−1^): ν = 3488, 2984, 2933, 2911, 1767, 1618, 1585, 1495, 1422, 1239, 1163, 1026, 762, 671. ^1^H NMR (600 MHz, CDCl_3_): δ = 7.55–7.53 (m, 1H), 7.33–7.30 (m, 1H), 7.16–7.13 (m, 1H), 7.07–7.04 (m, 1H), 4.98 (dd, ^2^*J*_(HCP)_ = 6.4 Hz, ^3^*J*_(HCCH)_ = 5.9 Hz, 1H, HC4), 4.11 (dd, ^3^*J*_(HCCP)_ = 6.4 Hz, ^3^*J*_(HCCH)_ = 5.9 Hz, 1H, HC3), 3.89–3.83 (m, 4H, 2 × CH_2_OP), 3.06 (s, 3H, CH_3_), 1.22 (t, ^3^*J*_(HCCH)_ = 7.0 Hz, 3H, C*H*_3_CH_2_OP), 1.19 (t, ^3^*J*_(HCCH)_ = 7.0 Hz, 3H, C*H*_3_CH_2_OP). ^13^C NMR (151 MHz, CDCl_3_): δ = 166.88 (d, *J* = 8.3 Hz, C=O), 161.26 (d, ^1^*J*_(CF)_ = 247.3 Hz, C2’), 131.12 (d, *J* = 3.2 Hz), 129.87 (d, *J* = 7.9 Hz), 123.67 (d, *J* = 3.2 Hz), 119.72 (d, *J* = 15.8 Hz), 114.74 (d, *J* = 21.2 Hz), 62.39 (d, ^2^*J*_(COP)_ = 6.6 Hz, CH_2_OP), 62.02 (d, ^2^*J*_(COP)_ = 6.5 Hz, CH_2_OP), 54.52 (d, ^1^*J*_(CP)_ = 170.6 Hz, C4), 51.21 (C3), 28.55 (CH_3_), 16.32 (d, ^3^*J*_(CCOP)_ = 5.4 Hz, *C*H_3_CH_2_OP), 16.25 (d, ^3^*J*_(CCOP)_ = 5.4 Hz, *C*H_3_CH_2_OP). ^31^P NMR (243 MHz, CDCl_3_): δ = 18.59. Anal. Cald for C_14_H_19_FNO_4_P: C, 53.33; H, 6.07; N, 4.44. Found: C, 53.03; H, 5.91; N, 4.68.

#### 3.2.6. trans-N-methyl-3-(2-fluorophenyl)-4-(diethoxyphosphoryl)azetidin-2-one (trans-**10b**)

Yellowish oil. IR (film, cm^−1^): ν = 3489, 2984, 2934, 1765, 1618, 1585, 1494, 1238, 1163, 1051, 1025, 959, 762, 671. ^1^H NMR (600 MHz, CDCl_3_): δ = 7.38–7.30 (m, 2H), 7.17–7.14 (m, 1H), 7.12–7.08 (m, 1H), 4.65 (dd, ^2^*J*_(HCP)_ = 9.1 Hz, ^3^*J*_(HCCH)_ = 2.4 Hz, 1H, HC4), 4.31–4.20 (m, 4H, 2 × CH_2_OP), 3.06 (s, 3H, CH_3_), 3.72 (dd, ^3^*J*_(HCCP)_ = 9.0 Hz, ^3^*J*_(HCCH)_ = 2.4 Hz, 1H, HC3), 1.40 (t, ^3^*J*_(HCCH)_ = 7.1 Hz, 3H, C*H*_3_CH_2_OP), 1.38 (t, ^3^*J*_(HCCH)_ = 6.7 Hz, 3H, C*H*_3_CH_2_OP). ^13^C NMR (151 MHz, CDCl_3_): δ = 167.03 (d, *J* = 13.1 Hz, C=O), 160.97 (d, ^1^*J*_(CF)_ = 247.8 Hz, C2′), 129.92 (d, *J* = 7.8 Hz), 129.53 (d, *J* = 4.0 Hz), 124.59 (d, *J* = 3.4 Hz), 119.72 (d, *J* = 15.5 Hz), 115.79 (d, *J* = 21.7 Hz), 63.12 (d, ^2^*J*_(COP)_ = 6.7 Hz, CH_2_OP), 62.61 (d, ^2^*J*_(COP)_ = 6.8 Hz, CH_2_OP), 55.77 (d, ^1^*J*_(CP)_ = 164.6 Hz, C4), 52.73 (C3), 28.73 (CH_3_), 16.60 (d, ^3^*J*_(CCOP)_ = 5.4 Hz, *C*H_3_CH_2_OP), 16.48 (d, ^3^*J*_(CCOP)_ = 5.8 Hz, *C*H_3_CH_2_OP). ^31^P NMR (243 MHz, CDCl_3_): δ = 20.06. Anal. Cald for C_14_H_19_FNO_4_P × 0.5 H_2_O: C, 51.85; H, 6.22; N, 4.32. Found: C, 52.00; H, 6.11; N, 4.60.

#### 3.2.7. trans-N-methyl-3-(3-fluorophenyl)-4-(diethoxyphosphoryl)azetidin-2-one (trans-**10c**)

Colorless oil. IR (film, cm^−1^): ν = 3491, 3062, 2984, 2912, 1763, 1615, 1589, 1422, 1241, 1050, 1023, 787, 686, 608. ^1^H NMR (600 MHz, CDCl_3_): δ = 7.32–7.29 (m, 1H), 7.11–7.10 (m, 1H), 7.05–7.03 (m, 1H), 6.99–6.96 (m, 1H), 4.52 (dd, ^2^*J*_(HCP)_ = 8.8 Hz, ^3^*J*_(HCCH)_ = 2.8 Hz, 1H, HC4), 4.27–4.19 (m, 4H, 2 × CH_2_OP), 3.65 (dd, ^3^*J*_(HCCP)_ = 8.8 Hz, ^3^*J*_(HCCH)_ = 2.8 Hz, 1H, HC3), 2.99 (s, 3H, CH_3_), 1.37 (t, ^3^*J*_(HCCH)_ = 7.1 Hz, 3H, C*H*_3_CH_2_OP), 1.36 (t, ^3^*J*_(HCCH)_ = 7.1 Hz, 3H, C*H*_3_CH_2_OP). ^13^C NMR (151 MHz, CDCl_3_): δ = 166.82 (d, *J* = 13.1 Hz, C=O), 162.96 (d, ^1^*J*_(CF)_ = 246.9 Hz, C3’), 136.55 (dd, *J* = 7.6 Hz, *J* = 1.8 Hz), 130.47 (d, *J* = 8.5 Hz), 122.94 (d, *J* = 2.9 Hz), 114.82 (d, *J* = 21.0 Hz), 114.30 (d, *J* = 22.1 Hz), 63.11 (d, ^2^*J*_(COP)_ = 6.7 Hz, CH_2_OP), 62.73 (d, ^2^*J*_(COP)_ = 7.2 Hz, CH_2_OP), 57.15 (C3), 55.95 (d, ^1^*J*_(CP)_ = 165.5 Hz, C4), 28.50 (CH_3_), 16.59 (d, ^3^*J*_(CCOP)_ = 5.4 Hz, *C*H_3_CH_2_OP), 16.53 (d, ^3^*J*_(CCOP)_ = 5.6 Hz, *C*H_3_CH_2_OP). ^31^P NMR (243 MHz, CDCl_3_): δ = 20.10. Anal. Cald for C_14_H_19_FNO_4_P·0.25H_2_O: C, 52.58; H, 6.15; N, 4.38. Found: C, 52.49; H, 6.34; N, 4.69.

#### 3.2.8. cis-N-methyl-3-(4-fluorophenyl)-4-(diethoxyphosphoryl)azetidin-2-one (cis-**10d**)

Colorless oil. IR (film, cm^−1^): ν = 3425, 2984, 2923, 1757, 1512, 1386, 1266, 1162, 1050, 1025, 785, 665. ^1^H NMR (600 MHz, CDCl_3_): δ = 7.40–7.38 (m, 2H), 7.04–7.00 (m, 2H), 4.77 (dd, ^2^*J*_(HCP)_ = 7.9 Hz, ^3^*J*_(HCCH)_ = 5.5 Hz, 1H, HC4), 4.04 (dd, ^3^*J*_(HCCP)_ = 6.0 Hz, ^3^*J*_(HCCH)_ = 5.5 Hz, 1H, HC3), 3.94–3.82 (m, 2H, CH_2_OP), 3.81–3.76 (m, 1H, CH_2_OP), 3.73–3.66 (m, 1H, CH_2_OP), 3.01 (s, 3H, C*H*_3_), 1.20 (t, ^3^*J*_(HCCH)_ = 7.0 Hz, 3H, C*H*_3_CH_2_OP), 1.16 (t, ^3^*J*_(HCCH)_ = 7.1 Hz, 3H, C*H*_3_CH_2_OP). ^13^C NMR (151 MHz, CDCl_3_): δ = 167.40 (d, *J* = 9.5 Hz, C=O), 162.46 (d, ^1^*J*_(CF)_ = 247.1 Hz, C4’), 131.27 (d, *J* = 8.1 Hz), 127.76 (d, *J* = 2.6 Hz), 114.97 (d, *J* = 21.8 Hz), 62.31 (d, ^2^*J*_(COP)_ = 7.0 Hz, CH_2_OP), 61.90 (d, ^2^*J*_(COP)_ = 7.3 Hz, CH_2_OP), 56.74 (d, ^2^*J*_(CCP)_ = 1.8 Hz, C3), 54.83 (d, ^1^*J*_(CP)_ = 172.1 Hz, C4), 28.51 (CH_3_), 16.40 (d, ^3^*J*_(CCOP)_ = 5.8 Hz, *C*H_3_CH_2_OP), 16.31 (d, ^3^*J*_(CCOP)_ = 5.6 Hz, *C*H_3_CH_2_OP). ^31^P NMR (243 MHz, CDCl_3_): δ = 18.93. Anal. Cald for C_14_H_19_FNO_4_P: C, 53.33; H, 6.07; N, 4.44. Found: C, 53.12; H, 5.92; N, 4.55.

#### 3.2.9. trans-N-methyl-3-(4-fluorophenyl)-4-(diethoxyphosphoryl)azetidin-2-one (trans-**10d**)

Colorless oil. IR (film, cm^−1^): ν = 3477, 3317, 2984, 2911, 1758, 1644, 1511, 1386, 1266, 1051, 1025, 785, 586. ^1^H NMR (600 MHz, CDCl_3_): δ = 7.33–7.31 (m, 2H), 7.08–7.04 (m, 2H), 4.53 (dd, ^2^*J*_(HCP)_ = 8.6 Hz, ^3^*J*_(HCCH)_ = 2.8 Hz, 1H, HC4), 4.29–4.21 (m, 4H, 2 × CH_2_OP), 3.64 (dd, ^3^*J*_(HCCP)_ = 9.0 Hz, ^3^*J*_(HCCH)_ = 2.8 Hz, 1H, HC3), 3.02 (s, 3H, C*H*_3_), 1.40 (t, ^3^*J*_(HCCH)_ = 7.1 Hz, 3H, C*H*_3_CH_2_OP), 1.39 (t, ^3^*J*_(HCCH)_ = 7.1 Hz, 3H, C*H*_3_CH_2_OP). ^13^C NMR (151 MHz, CDCl3): δ = 167.32 (d, *J* = 13.0 Hz, C=O), 162.37 (d, ^1^*J*_(CF)_ = 246.8 Hz, C4’), 130.05 (d, *J* = 2.9 Hz), 128.89 (d, *J* = 8.4 Hz), 115.85 (d, *J* = 21.7 Hz), 63.07 (d, ^2^*J*_(COP)_ = 6.6 Hz, CH_2_OP), 62.70 (d, ^2^*J*_(COP)_ = 7.0 Hz, CH_2_OP), 56.94 (d, ^2^*J*_(CCP)_ = 2.5 Hz, C3), 56.35 (d, ^1^*J*_(CP)_ = 165.2 Hz, C4), 28.51 (CH_3_), 16.63 (d, ^3^*J*_(CCOP)_ = 5.4 Hz, *C*H_3_CH_2_OP), 16.48 (d, ^3^*J*_(CCOP)_ = 5.5 Hz, *C*H_3_CH_2_OP). ^31^P NMR (243 MHz, CDCl_3_): δ = 20.31. Anal. Cald for C_14_H_19_FNO_4_P·0.5H_2_O: C, 51.85; H, 6.22; N, 4.32. Found: C, 51.70; H, 6.19; N, 4.53.

#### 3.2.10. cis-N-methyl-3-(2,4-difluorophenyl)-4-(diethoxyphosphoryl)azetidin-2-one (cis-**10e**)

Colorless oil. IR (film, cm^−1^): ν = 3475, 2986, 2923, 1763, 1509, 1430, 1388, 1236, 1142, 1052, 1027, 969, 790. ^1^H NMR (600 MHz, CDCl_3_): δ = 7.54–7.50 (m, 1H), 6.90–6.87 (m, 1H), 6.84–6.80 (m, 1H), 4.91 (dd, ^2^*J*_(HCP)_ = 6.8 Hz, ^3^*J*_(HCCH)_ = 6.1 Hz, 1H, HC4), 4.08 (dd, ^3^*J*_(HCCP)_ = 6.8 Hz, ^3^*J*_(HCCH)_ = 6.1 Hz, 1H, HC3), 3.95–3.87 (m, 4H, 2 × CH_2_OP), 3.05 (s, 3H, CH_3_), 1.24 (t, ^3^*J*_(HCCH)_ = 7.1 Hz, 3H, C*H*_3_CH_2_OP), 1.22 (t, ^3^*J*_(HCCH)_ = 7.1 Hz, 3H, C*H*_3_CH_2_OP). ^13^C NMR (151 MHz, CDCl_3_): δ = 166.58 (d, *J* = 8.2 Hz, C=O), 162.98 (dd, ^1^*J*_(CF)_ = 241.1 Hz, ^3^*J*_(CCCF)_ = 12.0 Hz, C2’), 161.32 (dd, ^1^*J*_(CF)_ = 241.0 Hz, ^3^*J*_(CCCF)_ = 11.8 Hz, C4’), 131.99 (dd, *J* = 9.8 Hz, *J* = 5.1 Hz), 115.81 (dd, *J* = 14.5 Hz, *J* = 3.1 Hz), 110.77 (dd, *J* = 21.2 Hz, *J* = 3.1 Hz,), 103.31 (dd, *J* = 25.6 Hz, *J* = 25.6 Hz,), 62.45 (d, ^2^*J*_(COP)_ = 6.8 Hz, CH_2_OP), 62.08 (d, ^2^*J*_(COP)_ = 6.8 Hz, CH_2_OP), 54.29 (d, ^1^*J*_(CP)_ = 171.4 Hz, C4), 50.63 (C3), 28.55 (CH_3_), 16.33 (d, ^3^*J*_(CCOP)_ = 5.6 Hz, *C*H_3_CH_2_OP), 16.27 (d, ^3^*J*_(CCOP)_ = 5.9 Hz, *C*H_3_CH_2_OP). ^31^P NMR (243 MHz, CDCl_3_): δ = 18.93. Anal. Cald for C_14_H_18_F_2_NO_4_P: C, 50.45; H, 5.44; N, 4.20. Found: C, 50.40; H, 5.43; N, 4.11.

#### 3.2.11. trans-N-methyl-3-(2,4-difluorophenyl)-4-(diethoxyphosphoryl)azetidin-2-one (trans-**10e**)

Colorless oil. IR (film, cm^−1^): ν = 3484, 3073, 2986, 2912, 1764, 1620, 1604, 1508, 1430, 1387, 1238, 1141, 1051, 969, 671. ^1^H NMR (600 MHz, CDCl_3_): δ = 7.36–7.29 (m, 1H), 6.91–6.84 (m, 2H), 4.60 (dd, ^2^*J*_(HCP)_ = 9.0 Hz, ^3^*J*_(HCCH)_ = 2.7 Hz, 1H, HC4), 4.28–4.20 (m, 4H, 2 × CH_2_OP), 3.67 (dd, ^3^*J*_(HCCP)_ = 8.8 Hz, ^3^*J*_(HCCH)_ = 2.7 Hz, 1H, HC3), 3.05 (s, 3H, CH_3_), 1.39 (t, ^3^*J*_(HCCH)_ = 5.7 Hz, 3H, C*H*_3_CH_2_OP), 1.37 (t, ^3^*J*_(HCCH)_ = 7.0 Hz, 3H, C*H*_3_CH_2_OP). ^13^C NMR (151 MHz, CDCl_3_): δ = 166.73 (d, *J* = 13.2 Hz, C=O), 162.84 (dd, ^1^*J*_(CF)_ = 250.0 Hz, ^3^*J*_(CCCF)_ = 12.1 Hz, C2’), 161.05 (dd, ^1^*J*_(CF)_ = 250.1 Hz, ^3^*J*_(CCCF)_ = 12.1 Hz, C4’), 130.31 (dd, *J* = 9.8 Hz, *J* = 5.5 Hz), 117.44 (dd, *J* = 14.8 Hz, *J* = 2.8 Hz), 111.78 (dd, *J* = 21.7 Hz, *J* = 3.8 Hz), 104.35 (dd, *J* = 25.4 Hz, *J* = 25.4 Hz), 63.15 (d, ^2^*J*_(COP)_ = 6.8 Hz, CH_2_OP), 62.66 (d, ^2^*J*_(COP)_ = 7.0 Hz, CH_2_OP), 55.78 (d, ^1^*J*_(CP)_ = 165.5 Hz, C4), 52.13 (d, ^2^*J*_(CCP)_ = 1.9 Hz, C3), 28.73 (CH_3_), 16.59 (d, ^3^*J*_(CCOP)_ = 5.3 Hz, *C*H_3_CH_2_OP), 16.48 (d, ^3^*J*_(CCOP)_ = 5.6 Hz, *C*H_3_CH_2_OP). ^31^P NMR (243 MHz, CDCl_3_): δ = 20.23. Anal. Cald for C_14_H_18_F_2_NO_4_P: C, 50.45; H, 5.44; N, 4.20. Found: C, 50.66; H, 5.22; N, 4.37.

#### 3.2.12. cis-N-methyl-3-(4-fluoro-3-methylphenyl)-4-(diethoxyphosphoryl)azetidin-2-one (cis-**10f**)

Colorless oil. IR (film, cm^−1^): ν = 3488, 2984, 2931, 2912, 1760, 1667, 1504, 1385, 1240, 1123, 969, 791. ^1^H NMR (600 MHz, CDCl_3_): δ = 7.26–7.24 (m, 1H), 7.20–7.17 (m, 1H), 6.98–6.95 (m, 1H), 4.74 (dd, ^2^*J*_(HCP)_ = 6.6 Hz, ^3^*J*_(HCCH)_ = 5.9 Hz, 1H, HC4), 4.03 (dd, ^3^*J*_(HCCP)_ = 6.6 Hz, ^3^*J*_(HCCH)_ = 5.9 Hz, 1H, HC3), 3.94–3.77 (m, 3H, CH_2_OP), 3.72–3.68 (m, 1H, CH_2_OP), 3.02 (s, 3H, CH_3_), 2.28 (s, 3H, CH_3_), 1.21 (t, ^3^*J*_(HCCH)_ = 7.1 Hz, 3H, C*H*_3_CH_2_OP), 1.17 (t, ^3^*J*_(HCCH)_ = 7.0 Hz, 3H, C*H*_3_CH_2_OP). ^13^C NMR (151 MHz, CDCl_3_): δ = 167.55 (d, *J* = 9.1 Hz, C=O), 160.98 (d, ^1^*J*_(CF)_ = 245.5 Hz, C4’), 132.60 (d, *J* = 5.0 Hz), 128.56 (d, *J* = 8.4 Hz), 127.36 (d, *J* = 2.8 Hz), 124.39 (d, *J* = 17.5 Hz), 114.59 (d, *J* = 22.6 Hz), 62.23 (d, ^2^*J*_(COP)_ = 6.8 Hz, CH_2_OP), 61.81 (d, ^2^*J*_(COP)_ = 7.2 Hz, CH_2_OP), 56.85 (C3), 54.94 (d, ^1^*J*_(CP)_ = 171.9 Hz, C4), 28.38 (CH_3_), 16.38 (d, ^3^*J*_(CCOP)_ = 5.6 Hz, *C*H_3_CH_2_OP), 16.29 (d, ^3^*J*_(CCOP)_ = 5.6 Hz, *C*H_3_CH_2_OP), 14.39 (d, ^3^*J* = 3.2 Hz, CH_3_). ^31^P NMR (243 MHz, CDCl_3_): δ = 19.04. Anal. Cald for C_15_H_21_FNO_4_P: C, 54.71; H, 6.43; N, 4.25. Found: C, 54.51; H, 6.37; N, 4.44.

#### 3.2.13. trans-N-methyl-3-(4-fluoro-3-methylphenyl)-4-(diethoxyphosphoryl)azetidin-2-one (trans-**10f**)

Colorless oil. IR (film, cm^−1^): ν = 3475, 2985, 2931, 2911, 1757, 1505, 1387, 1239, 1050, 1025, 970, 682. ^1^H NMR (600 MHz, CDCl_3_): δ = 7.18–7.16 (m, 1H), 7.13–7.10 (m, 1H), 7.00–6.98 (m, 1H), 4.49 (dd, ^2^*J*_(HCP)_ = 8.9 Hz, ^3^*J*_(HCCH)_ = 2.6 Hz, 1H, HC4), 3.63 (dd, ^3^*J*_(HCCP)_ = 9.1 Hz, ^3^*J*_(HCCH)_ = 2.6 Hz, 1H, HC3), 4.28–4.21 (m, 4H, 2 × CH_2_OP), 3.02 (s, 3H, CH_3_), 2.28 (d, ^4^*J* = 1.4 Hz, 3H, CH_3_), 1.40 (t, ^3^*J*_(HCCH)_ = 7.0 Hz, 3H, C*H*_3_CH_2_OP), 1.39 (t, ^3^*J*_(HCCH)_ = 7.1 Hz, 3H, C*H*_3_CH_2_OP). ^13^C NMR (151 MHz, CDCl_3_): δ = 167.51 (d, *J* = 13.1 Hz, C=O), 160.92 (d, ^1^*J*_(CF)_ = 245.5 Hz, C4’), 130.25 (d, *J* = 5.3 Hz), 129.67 (d, *J* = 3.0 Hz), 127.36 (d, *J* = 7.7 Hz), 125.49 (d, *J* = 17.6 Hz), 115.42 (d, *J* = 23.0 Hz), 63.03 (d, ^2^*J*_(COP)_ = 6.8 Hz, CH_2_OP), 62.68 (d, ^2^*J*_(COP)_ = 6.8 Hz, CH_2_OP), 57.02 (d, ^2^*J*_(CCP)_ = 2.5 Hz, C3), 56.41 (d, ^1^*J*_(CP)_ = 164.8 Hz, C4), 28.49 (CH_3_), 16.62 (d, ^3^*J*_(CCOP)_ = 5.3 Hz, *C*H_3_CH_2_OP), 16.57 (d, ^3^*J*_(CCOP)_ = 5.6 Hz, *C*H_3_CH_2_OP), 14.47 (d, ^3^*J* = 3.3 Hz, CH_3_). ^31^P NMR (243 MHz, CDCl_3_): δ = 20.40. Anal. Cald for C_15_H_21_FNO_4_P: C, 54.71; H, 6.43; N, 4.25. Found: 54.59; H, 6.33; N, 4.35.

#### 3.2.14. cis-N-benzyl-3-phenyl-4-(diethoxyphosphoryl)azetidin-2-one (cis-**11a**)

Colorless oil. Retention time: R*_t_*_,HPLC_ = 9.13 min. IR (film, cm^−1^): ν = 3488, 3088, 3031, 2983, 2930, 2910, 1760, 1604, 1498, 1390, 1239, 1049, 1022, 969, 701. ^1^H NMR (600 MHz, CDCl_3_): δ = 7.45–7.43 (m, 2H), 7.41–7.34 (m, 7H), 7.32–7.28 (m, 1H), 5.02 (d, ^2^*J* = 15.0 Hz, 1H, N-CH_2_), 4.79 (dd, ^2^*J*_(HCP)_ = 7.2 Hz, ^3^*J*_(HCCH)_ = 5.9 Hz, 1H, HC4), 4.21 (dd, ^2^*J* = 15.0 Hz, ^4^*J* = 1.3 Hz, 1H, N-CH_2_), 4.01 (dd, ^3^*J*_(HCCP)_ = 6.7 Hz, ^3^*J*_(HCCH)_ = 5.9 Hz, 1H, HC3), 3.85–3.79 (m, 1H, CH_2_OP), 3.74–3.66 (m, 2H, CH_2_OP), 3.60–3.53 (m, 1H, CH_2_OP), 1.15 (t, ^3^*J*_(HCCH)_ = 7.1 Hz, 3H, C*H*_3_CH_2_OP), 1.13 (t, ^3^*J*_(HCCH)_ = 7.1 Hz, 3H, C*H*_3_CH_2_OP). ^13^C NMR (151 MHz, CDCl_3_): δ = 167.51 (d, *J* = 9.9 Hz, C=O), 135.44, 131.90 (d, *J* = 2.8 Hz), 129.57, 128.80, 128.51, 128.11, 127.91, 127.89, 62.14 (d, ^2^*J*_(COP)_ = 6.7 Hz, CH_2_OP), 61.74 (d, ^2^*J*_(COP)_ = 6.8 Hz, CH_2_OP), 57.50 (d, ^2^*J*_(CCP)_ = 1.5 Hz, C3), 52.52 (d, ^1^*J*_(CP)_ = 172.6 Hz, C4), 45.64 (CH_2_-N), 16.35 (d, ^3^*J*_(CCOP)_ = 5.8 Hz, *C*H_3_CH_2_OP), 16.27 (d, ^3^*J*_(CCOP)_ = 6.0 Hz, *C*H_3_CH_2_OP). ^31^P NMR (243 MHz, CDCl_3_): δ = 19.15. Anal. Cald for C_20_H_24_NO_4_P·0.5H_2_O: C, 62.82; H, 6.59; N, 3.66. Found: C, 63.05; H, 6.25; N, 3.81.

#### 3.2.15. trans-N-benzyl-3-phenyl-4-(diethoxyphosphoryl)azetidin-2-one (trans-**11a**)

Colorless oil. Retention time: R*_t_*_,HPLC_ = 14.01 min. IR (film, cm^−1^): ν = 3287, 3030, 2961, 2927, 1636, 1553, 1453, 1432, 1259, 1162, 1132, 1030, 727, 694. ^1^H NMR (600 MHz, CDCl_3_): δ = 7.38–7.37 (m, 4H), 7.35–7.30 (m, 4H), 7.28–7.25 (m, 2H), 4.98 (d, ^2^*J* = 15.0 Hz, 1H, N-CH_2_), 4.59 (dd, ^2^*J*_(HCP)_ = 9.1 Hz, ^2^*J*_(HCCH)_ = 2.7 Hz, 1H, HC4), 4.24–4.13 (m, 5H, N-CH_2_, 2 × CH_2_OP), 3.61 (dd, ^3^*J*_(HCCP)_ = 9.0 Hz, ^3^*J*_(HCCH)_ = 2.7 Hz, 1H, HC3), 1.36 (t, ^3^*J*_(HCCH)_ = 7.0 Hz, 3H, C*H*_3_CH_2_OP), 1.35 (t, ^3^*J*_(HCCH)_ = 7.0 Hz, 3H, C*H*_3_CH_2_OP). ^13^C NMR (151 MHz, CDCl_3_): δ = 167.49 (d, *J* = 13.8 Hz, C=O), 135.52, 134.13 (d, *J* = 2.0 Hz), 128.94, 128.83, 128.60, 127.91, 127.86, 127.28, 63.06 (d, ^2^*J*_(COP)_ = 6.7 Hz, CH_2_OP), 62.55 (d, ^2^*J*_(COP)_ = 6.8 Hz, CH_2_OP), 57.43 (d, ^2^*J*_(CCP)_ = 1.9 Hz, C3), 54.07 (d, ^1^*J*_(CP)_ = 165.2 Hz, C4), 45.86 (CH_2_-N), 16.59 (d, ^3^*J*_(CCOP)_ = 5.4 Hz, *C*H_3_CH_2_OP), 16.53 (d, ^3^*J*_(CCOP)_ = 5.7 Hz, *C*H_3_CH_2_OP). ^31^P NMR (243 MHz, CDCl_3_): δ = 20.48. Anal. Cald for C_20_H_24_NO_4_P·0.25H_2_O: C, 63.57; H, 6.54; N, 3.71. Found: C, 63.61; H, 6.53; N, 3.78.

#### 3.2.16. cis-N-benzyl-3-(2-fluorophenyl)-4-(diethoxyphosphoryl)azetidin-2-one (cis-**11b**)

Colorless oil. Retention time: R*_t_*_,HPLC_ = 7.64 min. IR (film, cm^−1^): ν = 3424, 2985, 2934, 2912, 1760, 1665, 1495, 1456, 1390, 1238, 1050, 1026, 761. ^1^H NMR (600 MHz, CDCl_3_): δ = 7.59–7.56 (m, 1H), 7.41–7.38 (m, 4H), 7.35–7.29 (m, 2H), 7.17–7.14 (m, 1H), 7.05–7.02 (m, 1H), 5.00 (d, ^2^*J* = 14.9 Hz, 1H, N-CH_2_), 4.93 (dd, ^2^*J*_(HCP)_ = 7.0 Hz, ^2^*J*_(HCP)_ = 6.1 Hz, 1H, HC4), 4.23 (dd, ^2^*J* = 14.9 Hz, ^4^*J* = 1.4 Hz, 1H, N-CH_2_), 3.85–3.78 (m, 3H, CH_2_OP), 3.76–3.70 (m, 1H, CH_2_OP), 3.64 (dd, ^3^*J*_(HCCP)_ = 6.1 Hz, ^3^*J*_(HCCP)_ = 6.1 Hz, 1H, HC3), 1.18 (t, ^3^*J*_(HCCH)_ = 7.1 Hz, 3H, C*H*_3_CH_2_OP), 1.14 (t, ^3^*J*_(HCCH)_ = 7.0 Hz, 3H, C*H*_3_CH_2_OP). ^13^C NMR (151 MHz, CDCl_3_): δ = 166.70 (d, *J* = 8.6 Hz, C=O), 161.27 (d, ^1^*J*_(CF)_ = 247.6 Hz, C2’), 135.26, 131.18 (d, *J* = 3.2 Hz), 129.91 (d, *J* = 8.3 Hz), 128.84, 128.59, 127.94, 123.67 (d, *J* = 3.3 Hz), 119.68 (dd, *J* = 15.4 Hz, *J* = 2.2 Hz), 114.76 (d, *J* = 21.7 Hz), 62.37 (d, ^2^*J*_(COP)_ = 6.7 Hz, CH_2_OP), 61.90 (d, ^2^*J*_(COP)_ = 7.2 Hz, CH_2_OP), 52.07 (d, ^1^*J*_(CP)_ = 171.9 Hz, C4), 51.09 (d, ^2^*J*_(CCP)_ = 2.1 Hz, C3), 45.82 (CH_2_-N) 16.26 (d, ^3^*J*_(CCOP)_ = 5.8 Hz, *C*H_3_CH_2_OP), 16.21 (d, ^3^*J*_(CCOP)_ = 5.9 Hz, *C*H_3_CH_2_OP). ^31^P NMR (243 MHz, CDCl_3_): δ = 18.58. Anal. Cald for C_20_H_23_FNO_4_P: C, 61.38; H, 5.92; N, 3.58. Found: C, 61.49; H, 6.16; N, 3.63.

#### 3.2.17. trans-N-benzyl-3-(2-fluorophenyl)-4-(diethoxyphosphoryl)azetidin-2-one (trans-**11b**)

Colorless oil. Retention time: R*_t_*_,HPLC_ = 10.13 min. IR (film, cm^−1^): ν = 3475, 2984, 2911, 1761, 1585, 1495, 1387, 1239, 1051, 1025, 761, 621. ^1^H NMR (600 MHz, CDCl_3_): δ = 7.42–7.37 (m, 4H), 7.32–7.27 (m, 2H), 7.25–7.22 (m, 1H), 7.12–7.09 (m, 1H), 7.07–7.04 (m, 1H), 4.99 (d, ^2^*J* = 14.9 Hz, 1H, N-CH_2_), 4.70 (dd, ^2^*J*_(HCP)_ = 9.2 Hz, ^3^*J*_(HCCH)_ = 2.8 Hz, 1H, HC4), 4.24 (d, ^2^*J* = 14.9 Hz, 1H, N-CH_2_), 4.22–4.13 (m, 4H, 2 × CH_2_OP), 3.64 (dd, ^3^*J*_(HCCP)_ = 8.9 Hz, ^3^*J*_(HCCH)_ = 2.8 Hz 1H, HC3), 1.34 (t, ^3^*J*_(HCCH)_ = 6.9 Hz, 3H, C*H*_3_CH_2_OP), 1.33 (t, ^3^*J*_(HCCH)_ = 7.0 Hz, 3H, C*H*_3_CH_2_OP). ^13^C NMR (151 MHz, CDCl_3_): δ = 166.84 (d, *J* = 13.9 Hz, C=O), 160.95 (d, ^1^*J*_(CF)_ = 248.4 Hz, C2’), 135.30, 129.93 (d, *J* = 8.6 Hz), 129.45 (d, *J* = 3.4 Hz), 128.77, 128.73, 127.90, 124.59 (d, *J* = 3.4 Hz), 121.19 (dd, *J* = 17.3 Hz, *J* = 2.0 Hz), 115.80 (d, *J* = 21.6 Hz), 63.14 (d, ^2^*J*_(COP)_ = 6.6 Hz, CH_2_OP), 62.52 (d, ^2^*J*_(COP)_ = 6.9 Hz, CH_2_OP), 53.49 (d, ^1^*J*_(CP)_ = 166.6 Hz, C4), 52.25 (C3), 45.96 (N-CH_2_), 16.55 (d, ^3^*J*_(CCOP)_ = 5.4 Hz, *C*H_3_CH_2_OP), 16.44 (d, ^3^*J*_(CCOP)_ = 5.8 Hz, *C*H_3_CH_2_OP). ^31^P NMR (243 MHz, CDCl_3_): δ = 20.05. Anal. Cald for C_20_H_23_FNO_4_P: C, 61.38; H, 5.92; N, 3.58. Found: C, 61.66; H, 5.99; N, 3.56.

#### 3.2.18. trans-N-benzyl-3-(3-fluorophenyl)-4-(diethoxyphosphoryl)azetidin-2-one (trans-**11c**)

Yellowish oil. Retention time: R*_t_*_,HPLC_ = 7.45 min. IR (film, cm^−1^): ν = 3493, 2985, 2913, 1762, 1616, 1589, 1445, 1384, 1239, 1050, 1023, 967, 787, 687. ^1^H NMR (600 MHz, CDCl_3_): δ = 7.40–7.36 (m, 4H), 7.34–7.28 (m, 2H), 7.06–7.04 (m, 1H), 7.00–6.98 (m, 2H), 4.96 (d, ^2^*J* = 15.0 Hz, 1H, N-CH_2_), 4.58 (dd, ^2^*J*_(HCP)_ = 9.1 Hz, ^3^*J*_(HCCH)_ = 2.8 Hz, 1H, HC4), 4.24–4.13 (m, 5H, N-CH_2_, 2 × CH_2_OP), 3.58 (dd, ^3^*J*_(HCCP)_ = 8.6 Hz, ^3^*J*_(HCCPH)_ = 2.8 Hz, 1H, HC3), 1.37 (t, ^3^*J*_(HCCH)_ = 7.1 Hz, 3H, C*H*_3_CH_2_OP), 1.36 (t, ^3^*J*_(HCCH)_ = 7.1 Hz, 3H, C*H*_3_CH_2_OP). ^13^C NMR (151 MHz, CDCl_3_): δ = 166.80 (d, *J* = 13.8 Hz, C=O), 162.97 (d, ^1^*J*_(CF)_ = 247.2 Hz, C3’), 136.43 (dd, *J* = 7.7 Hz, *J* = 2.0 Hz, C1’), 135.35, 130.52 (d, *J* = 8.6 Hz), 128.89, 128.58, 128.02, 122.96 (d, *J* = 3.1 Hz), 114.89 (d, *J* = 21.0 Hz), 114.30 (d, *J* = 22.1 Hz), 63.16 (d, ^2^*J*_(COP)_ = 6.8 Hz, CH_2_OP), 62.64 (d, ^2^*J*_(COP)_ = 6.9 Hz, CH_2_OP), 56.85 (C3), 53.81 (d, ^1^*J*_(CP)_ = 166.1 Hz, C4), 45.92 (N-CH_2_), 16.59 (d, ^3^*J*_(CCOP)_ = 5.5 Hz, *C*H_3_CH_2_OP), 16.54 (d, ^3^*J*_(CCOP)_ = 5.6 Hz, *C*H_3_CH_2_OP). ^31^P NMR (243 MHz, CDCl_3_): δ = 20.90. Anal. Cald for C_20_H_23_FNO_4_P·0.25H_2_O: C, 60.68; H, 5.98; N, 3.54. Found: C, 60.91; H, 5.93; N, 3.55.

#### 3.2.19. cis-N-benzyl-3-(4-fluorophenyl)-4-(diethoxyphosphoryl)azetidin-2-one (cis-**11d**)

Yellowish oil. Retention time: R*_t_*_,HPLC_ = 4.42 min. IR (film, cm^−1^): ν = 3454, 3034, 2984, 2927, 1765, 1620, 1604, 1508, 1434, 1392, 1280, 1239, 1140, 1050, 1022, 969, 852, 701. ^1^H NMR (600 MHz, CDCl_3_): δ = 7.42–7.33 (m, 7H), 7.07–7.03 (m, 2H), 5.00 (d, ^2^*J* = 15.0 Hz, 1H, N-CH_2_), 4.75 (dd, ^2^*J*_(HCP)_ = 6.5 Hz, ^3^*J*_(HCCH)_ = 5.8 Hz, 1H, HC4), 4.20 (dd, ^2^*J* = 15.0 Hz, ^3^*J* = 1.6 Hz, 1H, N-CH_2_), 3.98 (dd, ^3^*J*_(HCCP)_ = 5.8 Hz, ^3^*J*_(HCCH)_ = 5.8 Hz, 1H, HC3), 3.89–3.82 (m, 1H, C*H*_2_OP), 3.79–3.71 (m, 2H, CH_2_OP), 3.70–3.63 (m, 1H, C*H*_2_OP), 1. 16 (t, ^3^*J*_(HCCH)_ = 7.1 Hz, 3H, C*H*_3_CH_2_OP), 1.15 (t, ^3^*J*_(HCCH)_ = 7.0 Hz, 3H, C*H*_3_CH_2_OP). ^13^C NMR (151 MHz, CDCl_3_): δ = 167.28 (d, *J* = 9.1 Hz, C=O), 162.50 (d, ^1^*J*_(CF)_ = 246.7 Hz, C4’), 135.31, 131.33 (d, *J* = 8.5 Hz), 128.83, 128.48, 127.94, 127.73 (dd, *J* = 3.1 Hz, *J* = 3.1 Hz), 115.00 (d, *J* = 21.7 Hz), 62.29 (d, ^2^*J*_(COP)_ = 6.7 Hz, CH_2_OP), 61.79 (d, ^2^*J*_(COP)_ = 7.4 Hz, CH_2_OP), 56.69 (d, ^2^*J*_(CCP)_ = 1.6 Hz, C3), 52.42 (d, ^1^*J*_(CP)_ = 172.3 Hz, C4), 45.69 (N-CH_2_), 16.36 (d, ^3^*J*_(CCOP)_ = 5.7 Hz, *C*H_3_CH_2_OP), 16.28 (d, ^3^*J*_(CCOP)_ = 6.1 Hz, *C*H_3_CH_2_OP). ^31^P NMR (243 MHz, CDCl_3_): δ = 18.95. Anal. Cald for C_20_H_23_FNO_4_P·0.25H_2_O: C, 60.68; H, 5.98; N, 3.54. Found: C, 60.54; H, 5.68; N, 3.50.

#### 3.2.20. trans-N-benzyl-3-(4-fluorophenyl)-4-(diethoxyphosphoryl)azetidin-2-one (trans-**11d**)

Colorless oil. Retention time: R*_t_*_,HPLC_ = 7.33 min. IR (film, cm^−1^): ν = 3477, 3066, 3035, 2984, 2929, 1758, 1606, 1512, 1394, 1299, 1237, 1161, 1051, 1024, 816, 767, 702. ^1^H NMR (600 MHz, CDCl_3_): δ = 7.39–7.36 (m, 4H), 7.34–7.32 (m, 1H), 7.24–7.22 (m, 2H), 7.04–7.01 (m, 2H), 4.96 (d, ^2^*J* = 15.0 Hz, 1H, N-CH_2_), 4.56 (dd, ^2^*J*_(HCP)_ = 9.0 Hz, ^3^*J*_(HCCH)_ = 2.8 Hz, 1H, HC4), 4.24–4.12 (m, 5H, N-CH_2_, 2 × CH_2_OP), 3.55 (dd, ^3^*J*_(HCCP)_ = 8.7 Hz, ^3^*J*_(HCCH)_ = 2.8 Hz, 1H, HC3), 1.37 (t, ^3^*J*_(HCCH)_ = 7.2 Hz, 3H, C*H*_3_CH_2_OP), 1.35 (t, ^3^*J*_(HCCH)_ = 7.2 Hz, 3H, C*H*_3_CH_2_OP). ^13^C NMR (151 MHz, CDCl_3_): δ = 167.29 (d, *J* = 13.8 Hz, C=O), 162.35 (d, ^1^*J*_(CF)_ = 247.1 Hz, C4’), 135.43, 130.05 (dd, *J* = 2.7 Hz, *J* = 2.7 Hz), 128.94 (d, *J* = 8.2 Hz), 128.88, 128.57, 127.99, 115.90 (d, *J* = 21.9 Hz), 63.10 (d, ^2^*J*_(COP)_ = 6.8 Hz, CH_2_OP), 62.60 (d, ^2^*J*_(COP)_ = 6.8 Hz, CH_2_OP), 56.60 (d, ^3^*J*_(CCOP)_ = 1.9 Hz, C3), 54.16 (d, ^1^*J*_(CP)_ = 165.7 Hz, C4), 45.88 (N-CH_2_), 16.59 (d, ^3^*J*_(CCOP)_ = 6.4 Hz, *C*H_3_CH_2_OP), 16.55 (d, ^3^*J*_(CCOP)_ = 6.4 Hz, *C*H_3_CH_2_OP). ^31^P NMR (243 MHz, CDCl_3_): δ = 20.26. Anal. Cald for C_20_H_23_FNO_4_P: C, 61.38; H, 5.92; N, 3.58. Found: C, 61.17; H, 5.77; N, 3.50.

#### 3.2.21. cis-N-benzyl-3-(2,4-difluorophenyl)-4-(diethoxyphosphoryl)azetidin-2-one (cis-**11e**)

Colorless oil. Retention time: R*_t_*_,HPLC_ = 4.88 min. IR (film, cm^−1^): ν = 3426, 3075, 2993, 2931, 1754, 1506, 1429, 1388, 1276, 1164, 1052, 1026, 964, 715. ^1^H NMR (600 MHz, CDCl_3_): δ = 7.56–7.52 (m, 1H), 7.40–7.37 (m, 4H), 7.35–7.32 (m, 1H), 6.90–6.87 (m, 1H), 6.82–6.78 (m, 1H), 4.98 (d, ^2^*J* = 15.0 Hz, 1H, N-CH_2_), 4.86 (dd, ^2^*J*_(HCP)_ = 7.0 Hz, ^3^*J*_(HCCH)_ = 6.1 Hz, 1H, HC4), 4.22 (dd, ^2^*J* = 15.0 Hz, ^4^*J* = 1.2 Hz, 1H, N-CH_2_), 4.00 (dd, ^3^*J*_(HCCP)_ = 6.1 Hz, ^3^*J*_(HCCH)_ = 6.1 Hz 1H, HC3), 3.88–3.83 (m, 3H, CH_2_OP), 3.81–3.75 (m, 1H, CH_2_OP), 1.20 (t, ^3^*J*_(HCCH)_ = 7.0 Hz, 3H, C*H*_3_CH_2_OP), 1.15 (t, ^3^*J*_(HCCH)_ = 7.1 Hz, 3H, C*H*_3_CH_2_OP). ^13^C NMR (151 MHz, CDCl_3_): δ = 166.39 (d, *J* = 8.5 Hz, C=O), 162.99 (dd, ^1^*J*_(CF)_ = 239.5 Hz, ^3^*J*_(CCCF)_ = 12.0 Hz, C2’), 161.33 (dd, *J* = 239.7 Hz, *J* = 11.8 Hz), 135.13, 132.06 (dd, *J* = 9.6 Hz, *J* = 4.7 Hz), 128.86, 128.57, 128.00, 115.73 (dd, *J* = 15.3 Hz, *J* = 2.5 Hz), 110.78 (dd, *J* = 21.2 Hz, *J* = 3.4 Hz), 103.33 (dd, *J* = 25.4 Hz, *J* = 25.4 Hz), 62.46 (d, ^2^*J*_(COP)_ = 7.0 Hz, CH_2_OP), 61.96 (d, ^2^*J*_(COP)_ = 6.8 Hz, CH_2_OP), 51.95 (d, ^1^*J*_(CP)_ = 171.8 Hz, C4), 50.52 (C3), 45.87 (N-CH_2_), 16.29 (d, ^3^*J*_(CCOP)_ = 5.8 Hz, *C*H_3_CH_2_OP), 16.24 (d, ^3^*J*_(CCOP)_ = 6.6 Hz, *C*H_3_CH_2_OP). ^31^P NMR (243 MHz, CDCl_3_): δ = 18.41. Anal. Cald for C_20_H_22_F_2_NO_4_P·0.25H_2_O: C, 58.04; H, 5.48; N, 3.38. Found: C, 57.93; H, 5.23; N, 3.44.

#### 3.2.22. trans-N-benzyl-3-(2,4-difluorophenyl)-4-(diethoxyphosphoryl)azetidin-2-one (trans-**11e**)

Colorless oil. Retention time: R*_t_*_,HPLC_ = 6.59 min. IR (film, cm^−1^): ν = 3489, 3066, 2984, 2930, 1766, 1508, 1435, 1278, 1164, 1050, 969, 701. ^1^H NMR (600 MHz, CDCl_3_): δ = 7.41–7.37 (m, 4H), 7.35–7.32 (m, 1H), 7.23–7.20 (m, 1H), 6.87–6.80 (m, 2H), 4.99 (d, ^2^*J* = 14.9 Hz, 1H, N-CH_2_), 4.65 (dd, ^2^*J*_(HCP)_ = 9.2 Hz, ^2^*J*_(HCCH)_ = 2.8 Hz, 1H, HC4), 4.25–4.13 (m, 5H, N-CH_2_, 2 × CH_2_OP), 3.59 (dd, ^3^*J*_(HCCP)_ = 8.6 Hz, ^3^*J*_(HCCH)_ = 2.8 Hz, 1H, HC3), 1.35 (t, ^3^*J*_(HCCH)_ = 7.1 Hz, 3H, C*H*_3_CH_2_OP), 1.34 (t, ^3^*J*_(HCCH)_ = 7.1 Hz, 3H, C*H*_3_CH_2_OP). ^13^C NMR (151 MHz, CDCl_3_): δ = 166.55 (d, *J* = 13.9 Hz, C=O), 162.80 (dd, ^1^*J*_(CF)_ = 250.1 Hz, ^3^*J*_(CCCF)_ = 12.0 Hz, C2’), 161.06 (dd, ^1^*J*_(CF)_ = 250.9 Hz, ^3^*J*_(CCCF)_ = 12.0 Hz, C4’), 135.21, 130.25 (dd, *J* = 9.8 Hz, *J* = 5.4 Hz), 128.81, 128.72, 127.98, 115.73 (ddd, *J* = 14.4 Hz, *J* = 5.2 Hz, *J* = 2.8 Hz), 111.81 (dd, *J* = 21.3 Hz, *J* = 3.4 Hz), 104.36 (dd, *J* = 25.7 Hz, *J* = 25.7 Hz), 63.18 (d, ^2^*J*_(COP)_ = 6.8 Hz, CH_2_OP), 62.56 (d, ^2^*J*_(COP)_ = 7.3 Hz, CH_2_OP), 53.48 (d, ^1^*J*_(CP)_ = 166.0 Hz, C4), 51.72 (d, ^2^*J*_(CCP)_ = 1.5 Hz, C3), 45.99 (N-CH_2_), 16.56 (d, ^3^*J*_(CCOP)_ = 5.4 Hz, *C*H_3_CH_2_OP), 16.46 (d, ^3^*J*_(CCOP)_ = 5.7 Hz, *C*H_3_CH_2_OP). ^31^P NMR (243 MHz, CDCl_3_): δ = 19.84. Anal. Cald for C_20_H_22_F_2_NO_4_P·0.25H_2_O: C, 58.04; H, 5.48; N, 3.38. Found: C, 58.00; H, 5.26; N, 3.50.

#### 3.2.23. cis-N-benzyl-3-(4-fluoro-3-methylphenyl)-4-(diethoxyphosphoryl)azetidin-2-one (cis-**11f**)

Yellowish oil. Retention time: R*_t_*_,HPLC_ = 5.15 min. IR (film, cm^−1^): ν = 3425, 3075, 2927, 2854, 1753, 1506, 1393, 1276, 1140, 1053, 1026, 965, 856, 701. ^1^H NMR (600 MHz, CDCl_3_): δ = 7.41–7.33 (m, 5H), 7.27–7.25 (m, 1H), 7.21–7.18 (m, 1H), 6.99–6.96 (m, 1H), 5.00 (d, ^2^*J* = 15.0 Hz, 1H, N-CH_2_), 4.71 (dd, ^2^*J*_(HCP)_ = 7.3 Hz, ^3^*J*_(HCCH)_ = 5.9 Hz, 1H, HC4), 4.19 (dd, ^2^*J* = 15.0 Hz, ^4^*J* = 1.4 Hz, 1H, N-CH_2_), 3.97 (dd, ^3^*J*_(HCCP)_ = 5.9 Hz, ^3^*J*_(HCCH)_ = 5.9 Hz 1H, HC3), 3.89–3.83 (m, 1H, CH_2_OP), 3.79–3.71 (m, 2H, CH_2_OP), 3.69–3.63 (m, 1H, CH_2_OP), 2.29 (d, ^4^*J* = 1.4 Hz, CH_3_), 1.16 (t, ^3^*J*_(HCCH)_ = 7.1 Hz, 3H, C*H*_3_CH_2_OP), 1.15 (t, ^3^*J*_(HCCH)_ = 7.1 Hz, 3H, C*H*_3_CH_2_OP). ^13^C NMR (151 MHz, CDCl_3_): δ = 167.45 (d, *J* = 9.9 Hz, C=O), 161.01 (d, ^1^*J*_(CF)_ = *J* = 245.5 Hz, C4’), 135.34, 132.66 (d, *J* = 5.5 Hz), 128.82, 128.62, 128.51 (d, *J* = 8.2 Hz), 127.93, 127.31 (dd, *J* = 2.9 Hz, *J* = 2.9 Hz), 124.42 (d, *J* = 17.6 Hz), 114.62 (d, *J* = 22.0 Hz), 62.25 (d, ^2^*J*_(COP)_ = 6.7 Hz, CH_2_OP), 61.73 (d, ^2^*J*_(COP)_ = 7.4 Hz, CH_2_OP), 56.78 (d, ^2^*J*_(CCP)_ = 2.1 Hz, C3), 52.47 (d, ^1^*J*_(CP)_ = 173.0 Hz, C4), 45.66 (N-CH_2_), 16.35 (d, ^3^*J*_(CCOP)_ = 5.7 Hz, *C*H_3_CH_2_OP), 16.28 (d, ^3^*J*_(CCOP)_ = 6.4 Hz, *C*H_3_CH_2_OP), 14.44 (d, ^3^*J* = 3.3 Hz, CH_3_). ^31^P NMR (243 MHz, CDCl_3_): δ = 19.08. Anal. Cald for C_21_H_25_FNO_4_P: C, 62.22; H, 6.22; N, 3.46. Found: C, 62.36; H, 6.41; N, 3.28.

#### 3.2.24. trans-N-benzyl-3-(4-fluoro-3-methylphenyl)-4-(diethoxyphosphoryl)azetidin-2-one (trans-**11f**)

Yellowish oil. Retention time: R*_t_*_,HPLC_ = 9.21 min. IR (film, cm^−1^): ν = 3456, 2985, 2933, 1668, 1583, 1566, 1454, 1396, 1249, 1162, 1024, 977, 953, 693. ^1^H NMR (600 MHz, CDCl_3_): δ = 7.39–7.37 (m, 4H), 7.35–7.32 (m, 1H), 7.04–7.01 (m, 2H), 6.96–6.93 (m, 1H), 4.96 (d, ^2^*J* = 14.9 Hz, 1H, N-CH_2_), 4.51 (dd, ^2^*J*_(HCP)_ = 9.0 Hz, ^3^*J*_(HCCH)_ = 2.8 Hz, 1H, HC4), 4.22–4.13 (m, 5H, 2 × CH_2_OP, N-CH_2_), 3.54 (dd, ^3^*J*_(HCCP)_ = 8.8 Hz, ^3^*J*_(HCCH)_ = 2.8 Hz 1H, HC3), 2.24 (d, ^4^*J* = 1.7 Hz, CH_3_), 1.37 (t, ^3^*J*_(HCCH)_ = 7.1 Hz, 3H, C*H*_3_CH_2_OP), 1.35 (t, ^3^*J*_(HCCH)_ = 7.0 Hz, 3H, C*H*_3_CH_2_OP). ^13^C NMR (151 MHz, CDCl_3_): δ = 167.52 (d, ^3^*J* = 13.5 Hz, C=O), 160.91 (d, ^1^*J*_(CF)_ = 245.4 Hz, C4’), 135.53, 130.25 (d, *J* = 5.5 Hz), 129.58 (d, *J* = 2.3 Hz), 128.86, 128.64, 127.98, 126.20 (d, *J* = 8.8 Hz), 125.52 (d, *J* = 17.6 Hz), 115.42 (d, *J* = 22.0 Hz), 63.07 (d, ^2^*J*_(COP)_ = 6.8 Hz, CH_2_OP), 62.57 (d, ^2^*J*_(COP)_ = 6.7 Hz, CH_2_OP), 56.66 (d, ^2^*J*_(CCP)_ = 2.1 Hz, C3), 54.20 (d, ^1^*J*_(CP)_ = 165.2 Hz, C4), 45.89 (N-CH_2_), 16.59 (d, ^3^*J*_(CCOP)_ = 5.5 Hz, *C*H_3_CH_2_OP), 16.54 (d, ^3^*J*_(CCOP)_ = 5.6 Hz, *C*H_3_CH_2_OP), 14.48 (d, ^3^*J* = 3.9 Hz, CH_3_). ^31^P NMR (243 MHz, CDCl_3_): δ = 20.37. Anal. Cald for C_21_H_25_FNO_4_P: C, 62.22; H, 6.22; N, 3.46. Found: C, 62.31; H, 6.35; N, 3.33.

### 3.3. Molecular Modelling

The preparation of compounds (generation of 3-dimensional conformations and protonation states at pH 7.0 +/−2.0) was performed with the use of LigPrep [41] from the Schrödinger Suite and Glide [42] from the same software package was used for docking (extra precision mode was applied). The dockings were performed to the crystal structure of beta-lactamase from *Staphylococcus aureus* (PDB ID: 3BLM) and PBP2a protein (PDP ID: 3ZFZ). The proteins were prepared for molecular modeling studies using the Protein Preparation Wizard [43] and the grid centerings were set to K234 (3BLM) and S403 (3ZFZ). The MD simulations were carried out using Schrodinger’s Desmond software [44] for each of the obtained ligand-receptor complexes (duration time = 500 ns; TIP3P as a solvent model [45]).

### 3.4. Antiviral Activity Assays

The compounds were evaluated against different herpesviruses, including herpes simplex virus type 1 (HSV-1) strain KOS, thymidine kinase-deficient (TK^−^) HSV-1 KOS strain resistant to ACV (ACV^r^), herpes simplex virus type 2 (HSV-2) strain G, varicella-zoster virus (VZV) strain Oka, TK^−^ VZV strain 07-1, human cytomegalovirus (HCMV) strains AD-169 and Davis as well as vaccinia virus, adenovirus-2, human coronavirus, parainfluenza-3 virus, reovirus-1, Sindbis virus, Coxsackie virus B4, Punta Toro virus, respiratory syncytial virus (RSV) and influenza A virus subtypes H1N1 (A/PR/8), H3N2 (A/HK/7/87) and influenza B virus (B/HK/5/72), were based on inhibition of virus-induced cytopathicity or plaque formation in human embryonic lung (HEL) fibroblasts, African green monkey kidney cells (Vero), human epithelial cervix carcinoma cells (HeLa) or Madin Darby canine kidney cells (MDCK). Confluent cell cultures in microtiter 96-well plates were inoculated with 100 CCID_50_ of virus (1 CCID_50_ being the virus dose to infect 50% of the cell cultures) or with 20 plaque forming units (PFU) and the cell cultures were incubated in the presence of varying concentrations of the test compounds. Viral cytopathicity or plaque formation (VZV) was recorded as soon as it reached completion in the control virus-infected cell cultures that were not treated with the test compounds. Antiviral activity was expressed as the EC_50_ or compound concentration required reducing virus-induced cytopathicity or viral plaque formation by 50%. Cytotoxicity of the test compounds was expressed as the minimum cytotoxic concentration (MCC) or the compound concentration that caused a microscopically detectable alteration of cell morphology.

### 3.5. Cytostatic Activity against Immortalized Cell Lines

All assays were performed in 96-well microtiter plates. To each well was added (5–7.5) × 10^4^ tumor cells and a given amount of the tested compound. The cells were allowed to proliferate at 37 °C in a humidified, CO_2_-controlled atmosphere. At the end of the incubation period, the cells were counted in a Coulter counter. The IC_50_ (50% inhibitory concentration) was defined as the concentration of the compound that inhibited cell proliferation by 50%.

### 3.6. Bacterial Assays

The in-vitro antibacterial property and the capacity of tested compounds to increase the efficacy of antibiotics were evaluated in two *Staphylococcus aureus* strains, i.e., the reference clonal complex 5 (CC5) methicillin-susceptible (MSSA) strain ATCC 25923, and the methicillin-resistant (MRSA) extensively drug-resistant (XDR) clinical isolate HEMSA-5 [46].

In order to assess the increase of antibiotic efficacy, the assays were conducted by determining if/to what extent the investigated compounds reduce MICs of oxacillin by means of a serial dilution broth microplate method, in accordance with the CLSI requirements [47]. The concentrations of compounds used in the MICs reduction assay were no greater than 1/4 of their respective MICs to ensure that cell viability was not affected by the intrinsic antibacterial activity of the molecules. Serial two-fold dilutions of oxacillin (Sigma-Aldrich; St. Louis, MI, USA, cat. no. 28221), were prepared in 65 mL of the Mueller–Hinton broth (Merck; Darmstadt, Germany, cat. no. 1102930500). Suitable concentrations of the compounds (total volume 10 mL) were then added. Bacterial suspensions were diluted to OD ¼ 0.5. The resulting suspensions were then diluted 1:100 and added in the volume of 75 mL into the oxacillin serial dilutions with the compounds. The results were read after 20-h incubation at 37 °C.

**Figure 10:**
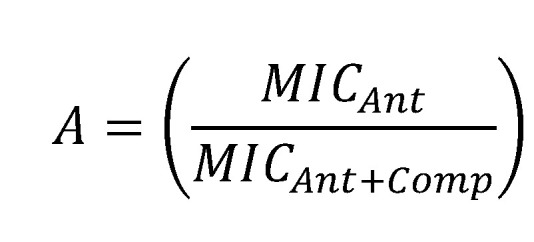
Activity gain. MIC_Ant_ corresponds to the MIC of oxacillin in the absence of a compound tested and MIC_Ant_ + _Comp_ refers to the MIC of oxacillin paired with a compound tested.

The ability of compounds to improve antibiotic efficacy was expressed as the activity gain [A] parameter calculated according to the formula given in Figure 10.

## 4. Conclusions

A new series of N-substituted 3-aryl-4-(diethoxyphosphoryl)azetidin-2-ones *cis*-**10**/*trans*-**10** and *cis*-**11**/*trans*-**11** was efficiently synthesized from N-methyl- or N-benzyl-(diethyoxyphosphoryl)nitrone **12** and **13** with the respective aryl alkynes **14a**-**14f** via the Kinugase reaction. All synthesized compounds were tested for their antiviral activities toward DNA and RNA viruses. Among them, compound *trans*-**11f** exhibited activity against human coronavirus (229E) with EC_50_ = 45 µM, while the other isomer *cis*-**11f** was active against influenza A virus H1N1 subtype (EC_50_ = 12 µM by visual CPE score; EC_50_ = 8.3 µM by TMS score; MCC > 100 µM, CC_50_ = 39.9 µM). Several azetidin-2-ones **10** and **11** showed moderate cytostatic activity toward Capan-1, Hap1 and HCT-116 cells values of IC_50_ in the range 14.5–97.9 µM.

According to our knowledge, this study allowed for identifying the first azetidinone-derived “adjuvant” of oxacillin with significant ability to enhance efficacy of this antibiotic in the highly resistant *S. aureus* strain HEMSA 5. The computer-aided insight into potential mechanisms of action indicated that the enantiomer (3*R*,4*S*)-**11f**, rather than (3*S*,4*R*)-**11f**, can be responsible for such a promising biological activity due to the potency in displacing oxacillin at β-lactamase, thus protecting this antibiotic from undesirable biotransformation. These results demonstrate that both the presence of the respective aryl group and the appropriate configuration at the stereogenic centers in azetidin-2-one ring a play crucial role in overcoming bacterial MDR mechanisms. This finding may be significant in the extended search for effective adjuvants for the treatment of infection diseases, in which the enantiomer of the obtained compound *trans*-**11f**, namely (3*R*,4*S*)-*N*-benzyl-3-(4-fluoro-3-methylphenyl)-4-(diethoxyphosphoryl)azetidin-2-one (3*R*,4*S*)-**11f**, can be used as a lead structure for further pharmacomodulations and a broader understanding of molecular mechanisms.

## Figures and Tables

**Table 1:** Cycloaddtion of nitrone **12** with arylalkynes **14**.

Entry	Alkyne 14 (R)	^31^P NMR δ [ppm]		Procedure	*cis/trans* Ratio ^1^	Total Yield (%) ^2^	Yield of *cis*/ *trans* Isomers (*cis-*10/*trans*-10) ^3^
		*cis*-10	*trans*-10				
**a** [33]	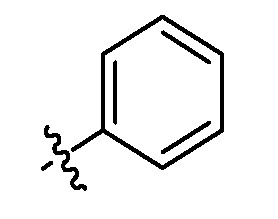	**19.08**	**20.52**	**A**	22:78	76	*cis*-**10a**–11% *trans*-**10a**–54%
				B	41:59	80	*cis*-**10a**–21% *trans*-**10a**–40%
**b** [33]	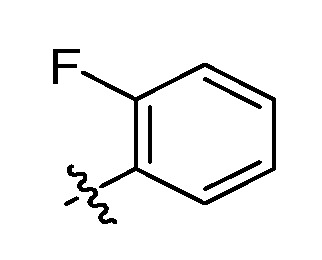	18.59	20.06	A	51:49	84	*cis*-**10b**–33% *trans*-**10b**–24%
				B	43:57	86	*cis*-**10b**–19% *trans*-**10b**–42%
**c** [33]	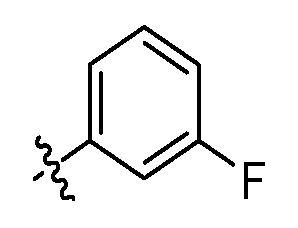	18.64	20.10	A	20:80	74	*trans*-**10c**–65%
				B	36:64	88	*cis*-**10c**–7% *trans*-**10c**–55%
**d**	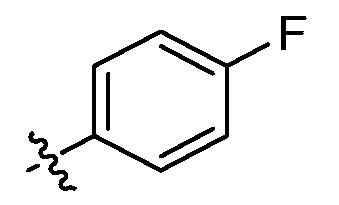	18.93	20.31	A	59:41	64	*cis*-**10d**–30% *trans*-**10d**–14%
				B	30:70	65	*cis*-**10d**–17% *trans*-**10d**–31%
**e** [33]	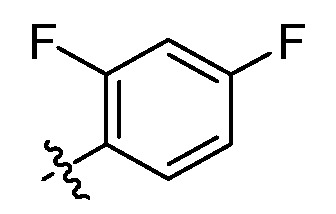	18.93	20.23	A	48:52	92	*cis*-**10e**–4% *trans*-**10e**–16%
				B	34:66	60	*cis*-**10e**–23%
**f**	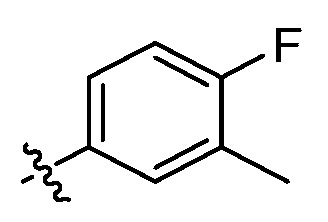	19.04	20.40	A	37:63	65	*cis*-**10f**–10% *trans*-**10f**–37%
				B	32:68	65	*cis*-**10f**–17% *trans*-**10f**–31%

^1^ The *cis*/*trans* ratio was calculated from the ^31^P NMR spectra of crude reaction mixtures. ^2^ Total yield = yield of pure isomers and mixtures of isomers after column chromatography. ^3^ Yield of pure isomers obtained after chromatographic purification.

**Table 2:** Cycloaddtion of nitrone **13** with arylalkynes **14**.

Entry	Alkyne 14 (R)	^31^P NMR δ [ppm]		Procedure	*cis/trans* Ratio ^1^	Total Yield (%) ^2^	Yield of *cis*/*trans* Isomers (*cis-*11/*trans*-11) ^3^
		*cis*-11	*trans*-11				
**a**	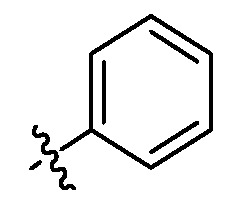	19.15	20.48	A	21:79	57	*cis*-**11a**–8% *trans*-**11a**–24%
				B	26:74	79	*cis*-**11a**–10% *trans*-**11a**–36%
**b**	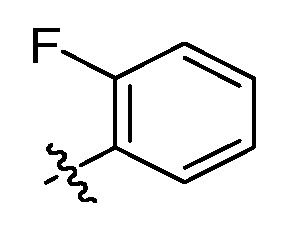	18.58	20.05	A	31:69	78	*cis*-**11b**–20% *trans*-**11b**–26%
				B	30:70	65	*cis*-**11b**–18% *trans*-**11b** –33%
**c**	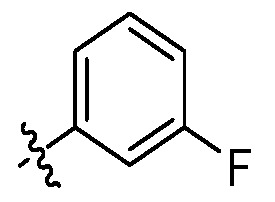	20.51	20.90	A	13:87	63	*trans*-**11c**–44%
				B	28:72	82	*trans*-**11c** –56%
**d**	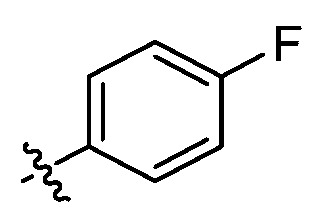	18.95	20.26	A	41:59	66	*cis*-**11d**–10% *trans*-**11d**–20%
				B	25:75	54	*cis*-**11d**–5% *trans*-**11d**–37%
**e**	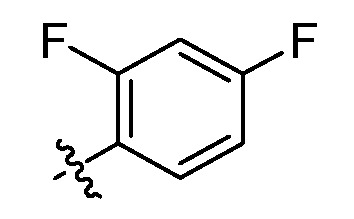	18.41	19.84	A	68:32	67	*cis*-**11e**–30% *trans*-**11e**–20%
				B	33:67	61	*cis*-**11e** –24% *trans*-**11e**–9%
**f**	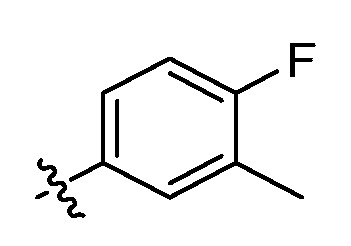	19.08	20.37	A	35:65	45	*cis*-**11f**–7% *trans*-**11f**–14%
				B	30:70	54	*cis*-**11f**–6% *trans*-**11f**–35%

^1^ The *cis*/*trans* ratio was calculated from the ^31^P NMR spectra of crude reaction mixtures. ^2^ Total yield = yield of pure isomers and mixtures of isomers after column chromatography. ^3^ Yield of pure isomers obtained after chromatographic purification.

**Table 3:** The ability of azetidinone compounds to enhance antibacterial activity of oxacillin against *S. aureus* ATCC 25923 and *S. aureus* MRSA HEMSA 5.

Cpd ^1^	*S. aureus* ATCC 25923			MRSA HEMSA 5		
	OXA MIC [µg/mL]	OXA+ Cpd MIC [µg/mL]	Activity Gain [A] ^2^	OXA MIC [µg/mL]	OXA+ Cpd MIC [µg/mL]	Activity Gain [A] ^2^
*trans*-**10a**	0.5	0.5	1	512	512	1
*cis*-**10a**	0.5	1	0.5	512	256–512	1–2
*trans*-**10b**	0.5	1	0.5	512	512	1
*cis*-**10b**	0.5	1	0.5	512	512	1
*trans*-**10c**	0.5	1	0.5	512	512	1
*trans*-**10d**	0.5	1	0.5	512	512	1
*cis*-**10d**	0.5	1	0.5	512	512	1
*trans*-**10e**	0.5	>1	<0.5	512	512	1
*cis*-**10e**	0.5	0.5	1	512	512	1
*trans*-**10f**	0.5	1	0.5	512	512	1
*cis*-**10f**	0.5	1	0.5	512	256–512	1–2
*trans*-**11a**	0.5	>1	<0.5	512	256–512	1–2
*cis*-**11a**	0.5	1	0.5	512	256–512	1–2
*trans*-**11b**	0.5	>1	<0.5	512	256–512	1–2
*cis*-**11b**	0.5	0.5	1	512	512	1
*trans*-**11c**	0.5	>1	<0.5	512	512	1
*trans*-**11d**	0.5	1	0.5	512	256	2
*cis*-**11d**	0.5	0.5	1	512	512	1
*trans*-**11e**	0.5	1	0.5	512	512	1
*cis*-**11e**	0.5	1	0.5	512	512	1
*trans*-**11f**	0.5	1	0.5	512	32	16
*cis*-**11f**	0.5	>1	<0.5	512	256–512	1–2

^1^ The compounds were tested at 0.5 mM, i.e., the concentration corresponding to the MIC/4. ^2^ “Adjuvant” activity observed if A ≥ 4.

**Table 4:** Inhibitory effect of azetidinones *cis*-**10**/*trans*-**10** and *cis*-**11**/*trans*-**11** against the proliferation of cancerous cells.

Cpd	IC_50_ (µM)					
	hTERT RPE-1	Capan-1	Hap1	HCT-116	NCI-H460	DND-41
*trans*-**10a**	>100	46.2	>100	>100	54.6	>100
*cis*-**10a**	>100	37.8	46.3	>100	>100	>100
*trans*-**10b**	>100	53.1	38.7	44.1	34.8	>100
*cis*-**10b**	>100	31.9	14.5	>100	45.2	>100
*trans*-**10c**	>100	19.6	69.1	>100	62.8	>100
*trans*-**10d**	>100	45.7	58.1	>100	41.3	>100
*cis*-**10d**	>100	>100	70.3	86.5	71.0	>100
*trans*-**10e**	>100	51.7	51.7	>100	67.6	>100
*cis*-**10e**	>100	56.8	>100	>100	92.6	>100
*trans*-**10f**	90.2	47.7	41.8	>100	59.7	>100
*cis*-**10f**	>100	61.4	97.9	>100	87.0	>100
*trans*-**11a**	>100	61.0	63.6	>100	50.9	>100
*cis*-**11a**	>100	95.2	>100	87.9	78.8	>100
*trans*-**11b**	>100	51.9	>100	>100	56.7	>100
*cis*-**11b**	>100	53.0	>100	74.1	72.8	>100
*trans*-**11c**	45.9	40.1	56.1	45.5	44.3	>100
*trans*-**11d**	73.0	35.8	48.3	44.3	28.4	>100
*cis*-**11d**	>100	35.9	61.9	54.1	45.9	>100
*trans*-**11e**	25.6	36.5	42.9	36.6	24.4	>100
*cis*-**11e**	>100	48.9	>100	>100	43.0	>100
*trans*-**11f**	33.5	34.8	46.5	35.3	37.0	65.8
*cis*-**11f**	>100	38.4	>100	>100	53.6	>100
Docetaxel	25.0	0.95	1.19	0.25	0.89	1.63
Etoposide	0.23	0.15	0.04	1.03	1.35	0.06
Stauroporine	0.25	0.66	3.55	0.09	11.50	21.5

## Data Availability

Not applicable.

## Supplementary Materials

The following are available online at https://www.mdpi.com/article/10.3390/ijms22158032/s1.
