# Supplementary material for: Novel N-Substituted 3-Aryl-4-(diethoxyphosphoryl)azetidin-2-ones as Antibiotic Enhancers and Antiviral Agents in Search for a Successful Treatment of Complex Infections
Source: Int J Mol Sci. 2021 Jul 27;22(15):8032. doi: 10.3390/ijms22158032 (PMC8348901; doi:10.3390/ijms22158032)
Supplement: Supplementary file 1 [file ijms-22-08032-s001.zip › ijms-1296332-supplementary.pdf]

Supplementary Materials

NMR spectra for compounds *cis*-**10**/*trans*-**10** and *cis*-**11**/*trans*-**11**:

Figure S1: ^{1}H NMR Spectrum for *cis*-**10b** in $CDCl_3$
Figure S2: ^{13}C NMR Spectrum for *cis*-**10b** in $CDCl_3$
Figure S3: ^{31}P NMR Spectrum for *cis*-**10b** in $CDCl_3$
Figure S4: ^{1}H NMR Spectrum for *trans*-**10b** in $CDCl_3$
Figure S5: ^{13}C NMR Spectrum for *trans*-**10b** in $CDCl_3$
Figure S6: ^{31}P NMR Spectrum for *trans*-**10b** in $CDCl_3$
Figure S7: ^{1}H NMR Spectrum for *trans*-**10c** in $CDCl_3$
Figure S8: ^{13}C NMR Spectrum for *trans*-**10c** in $CDCl_3$
Figure S9: ^{31}P NMR Spectrum for *trans*-**10c** in $CDCl_3$
Figure S10: ^{1}H NMR Spectrum for *cis*-**10d** in $CDCl_3$
Figure S11: ^{13}C NMR Spectrum for *cis*-**10d** in $CDCl_3$
Figure S12: ^{31}P NMR Spectrum for *cis*-**10d** in $CDCl_3$
Figure S13: ^{1}H NMR Spectrum for *trans*-**10d** in $CDCl_3$
Figure S14: ^{13}C NMR Spectrum for *trans*-**10d** in $CDCl_3$
Figure S15: ^{31}P NMR Spectrum for *trans*-**10d** in $CDCl_3$
Figure S16: ^{1}H NMR Spectrum for *cis*-**10e** in $CDCl_3$
Figure S17: ^{13}C NMR Spectrum for *cis*-**10e** in $CDCl_3$
Figure S18: ^{31}P NMR Spectrum for *cis*-**10e** in $CDCl_3$
Figure S19: ^{1}H NMR Spectrum for *trans*-**10e** in $CDCl_3$
Figure S20: ^{13}C NMR Spectrum for *trans*-**10e** in $CDCl_3$
Figure S21: ^{31}P NMR Spectrum for *trans*-**10e** in $CDCl_3$
Figure S22: ^{1}H NMR Spectrum for *cis*-**10f** in $CDCl_3$
Figure S23: ^{13}C NMR Spectrum for *cis*-**10f** in $CDCl_3$
Figure S24: ^{31}P NMR Spectrum for *cis*-**10f** in $CDCl_3$
Figure S25: ^{1}H NMR Spectrum for *trans*-**10f** in $CDCl_3$
Figure S26: ^{13}C NMR Spectrum for *trans*-**10f** in $CDCl_3$
Figure S27: ^{31}P NMR Spectrum for *trans*-**10f** in $CDCl_3$
Figure S28: ^{1}H NMR Spectrum for *cis*-**11a** in $CDCl_3$
Figure S29: ^{13}C NMR Spectrum for *cis*-**11a** in $CDCl_3$
Figure S30: ^{31}P NMR Spectrum for *cis*-**11a** in $CDCl_3$
Figure S31: ^{1}H NMR Spectrum for *trans*-**11a** in $CDCl_3$
Figure S32: ^{13}C NMR Spectrum for *trans*-**11a** in $CDCl_3$
Figure S33: ^{31}P NMR Spectrum for *trans*-**11a** in $CDCl_3$
Figure S34: ^{1}H NMR Spectrum for *cis*-**11b** in $CDCl_3$
Figure S35: ^{13}C NMR Spectrum for *cis*-**11b** in $CDCl_3$
Figure S36: ^{31}P NMR Spectrum for *cis*-**11b** in $CDCl_3$
Figure S37: ^{1}H NMR Spectrum for *trans*-**11b** in $CDCl_3$
Figure S38: ^{13}C NMR Spectrum for *trans*-**11b** in $CDCl_3$
Figure S39: ^{31}P NMR Spectrum for *trans*-**11b** in $CDCl_3$
Figure S40: ^{1}H NMR Spectrum for *trans*-**11c** in $CDCl_3$
Figure S41: ^{13}C NMR Spectrum for *trans*-**11c** in $CDCl_3$
Figure S42: ^{31}P NMR Spectrum for *trans*-**11c** in $CDCl_3$
Figure S43: ^{1}H NMR Spectrum for *cis*-**11d** in $CDCl_3$
Figure S44: ^{13}C NMR Spectrum for *cis*-**11d** in $CDCl_3$
Figure S45: ^{31}P NMR Spectrum for *cis*-**11d** in $CDCl_3$
Figure S46: ^{1}H NMR Spectrum for *trans*-**11d** in $CDCl_3$
Figure S47: ^{13}C NMR Spectrum for *trans*-**11d** in $CDCl_3$
Figure S48: ^{31}P NMR Spectrum for *trans*-**11d** in $CDCl_3$
Figure S49: ^{1}H NMR Spectrum for *cis*-**11e** in $CDCl_3$
Figure S50: ^{13}C NMR Spectrum for *cis*-**11e** in $CDCl_3$
Figure S51: ^{31}P NMR Spectrum for *cis*-**11e** in $CDCl_3$

Figure S52: ^{1}H NMR Spectrum for *trans*-**11e** in $CDCl_3$
Figure S53: ^{13}C NMR Spectrum for *trans*-**11e** in $CDCl_3$
Figure S54: ^{31}P NMR Spectrum for *trans*-**11e** in $CDCl_3$
Figure S55: ^{1}H NMR Spectrum for *cis*-**11f** in $CDCl_3$
Figure S56: ^{13}C NMR Spectrum for *cis*-**11f** in $CDCl_3$
Figure S57: ^{31}P NMR Spectrum for *cis*-**11f** in $CDCl_3$
Figure S58: ^{1}H NMR Spectrum for *trans*-**11f** in $CDCl_3$
Figure S59: ^{13}C NMR Spectrum for *trans*-**11f** in $CDCl_3$
Figure S60: ^{31}P NMR Spectrum for *trans*-**11f** in $CDCl_3$

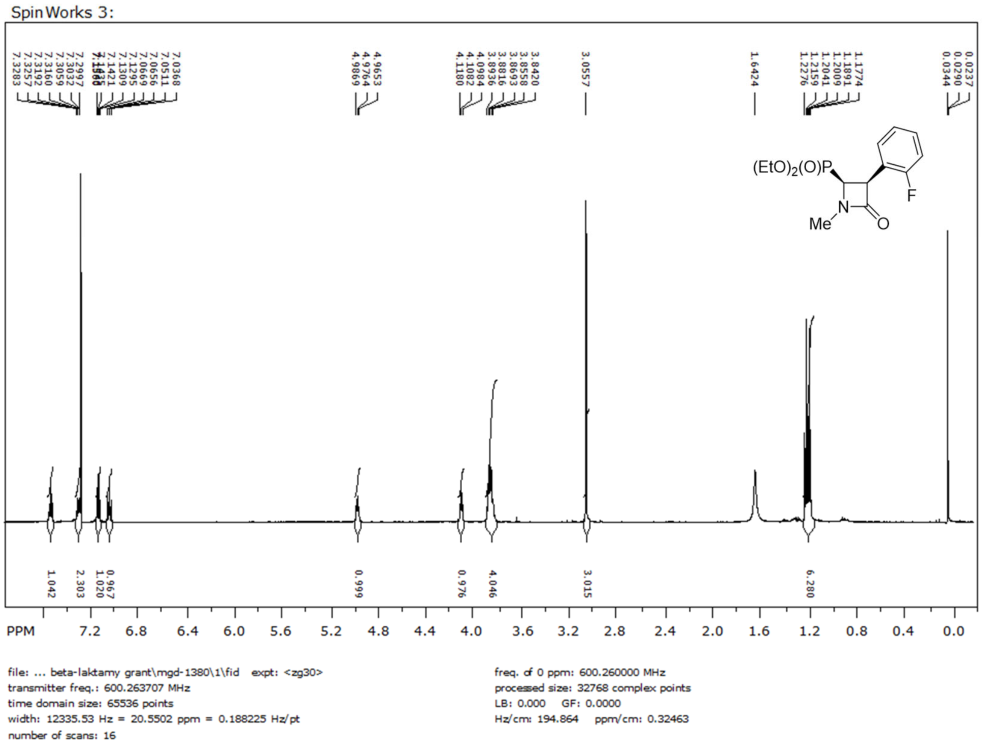

Figure S1. ^{1}H NMR Spectrum for *cis*-**10b** in $CDCl_3$.

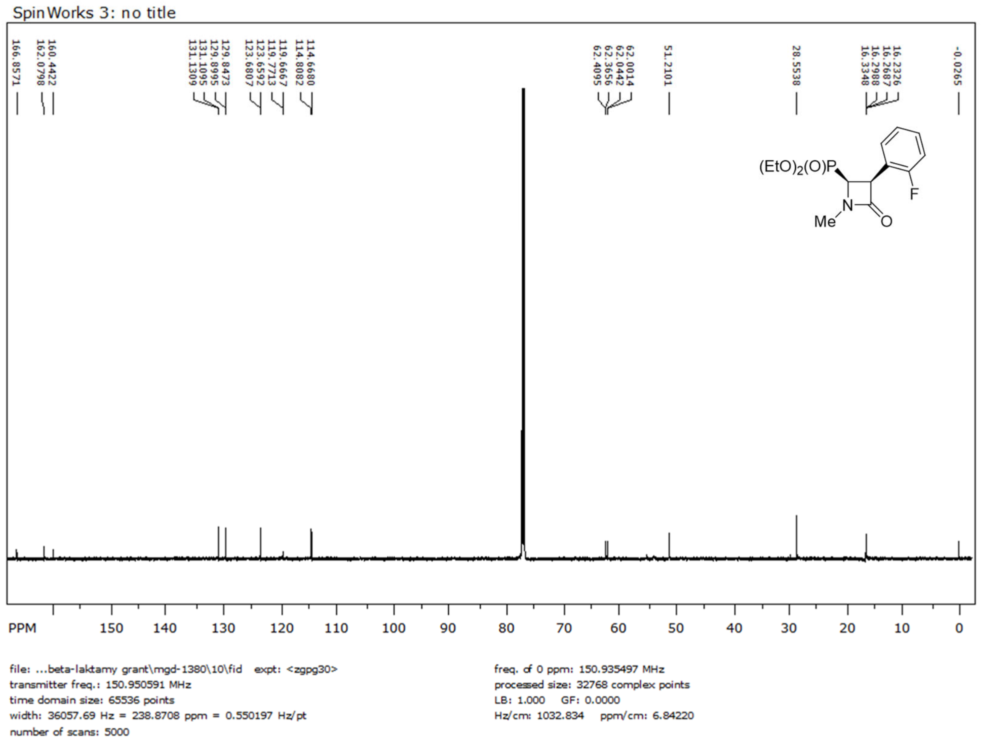

Figure S2. ^{13}C NMR Spectrum for *cis*-**10b** in $CDCl_3$.

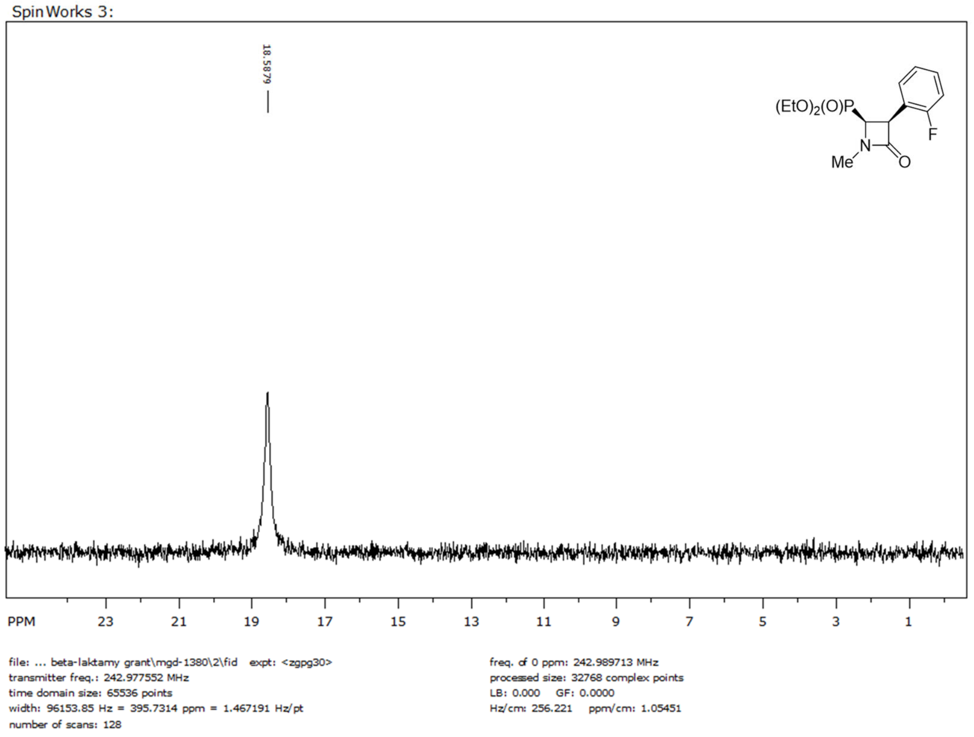

Figure S3. ^{31}P NMR Spectrum for *cis*-**10b** in $CDCl_3$.

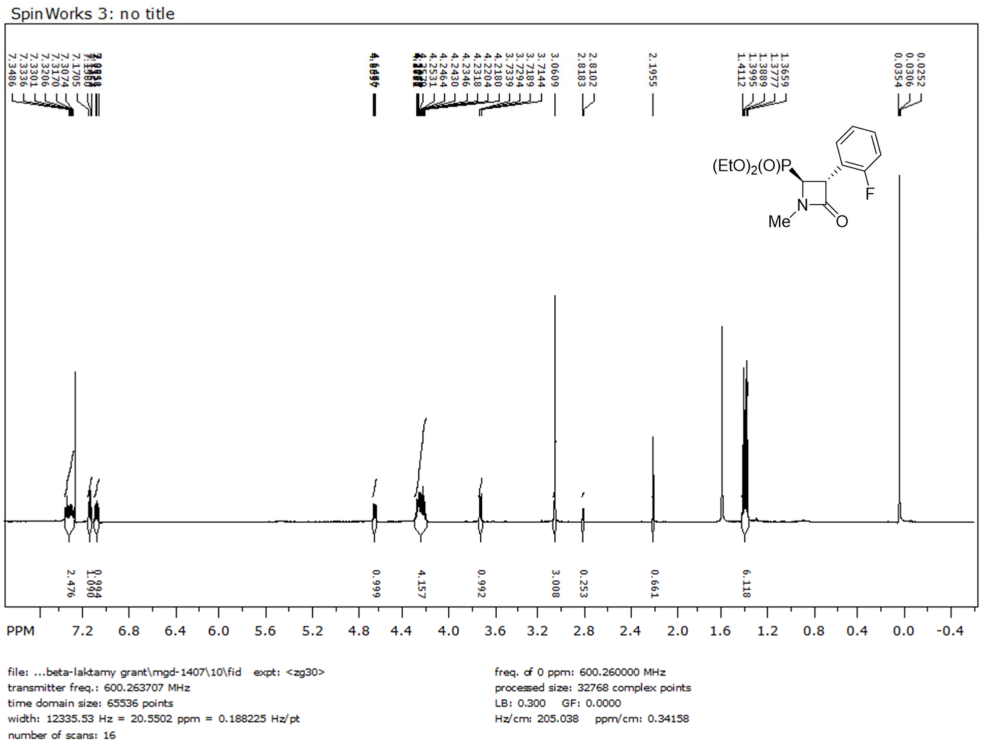

Figure S4: ^{1}H NMR Spectrum for *trans*-**10b** in $CDCl_3$

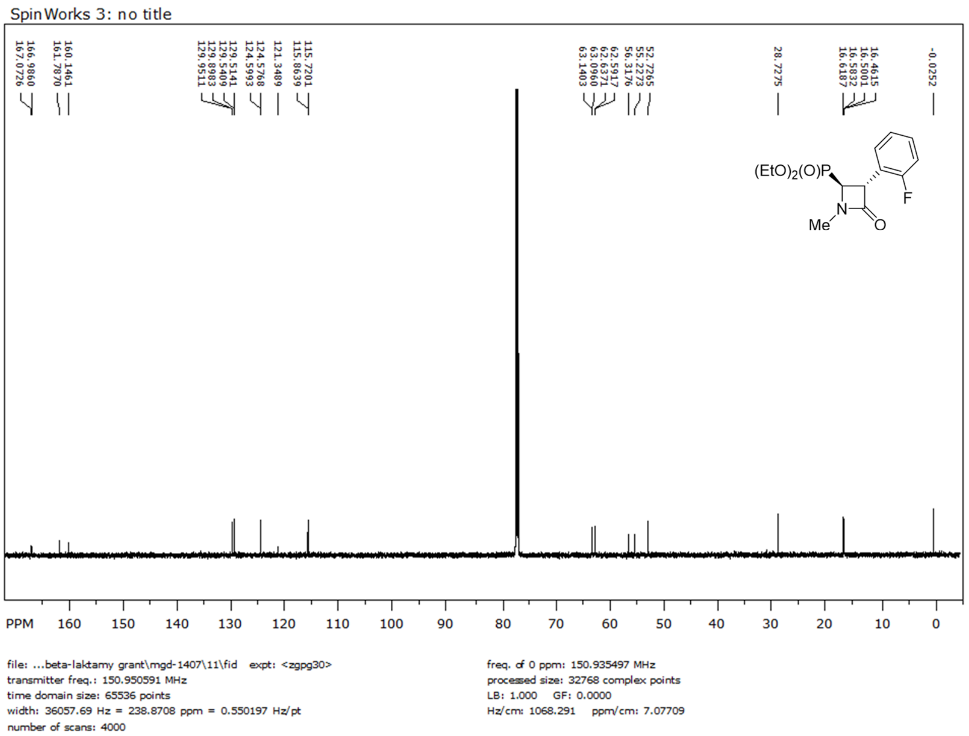

Figure S5: ^{13}C NMR Spectrum for *trans*-**10b** in $CDCl_3$

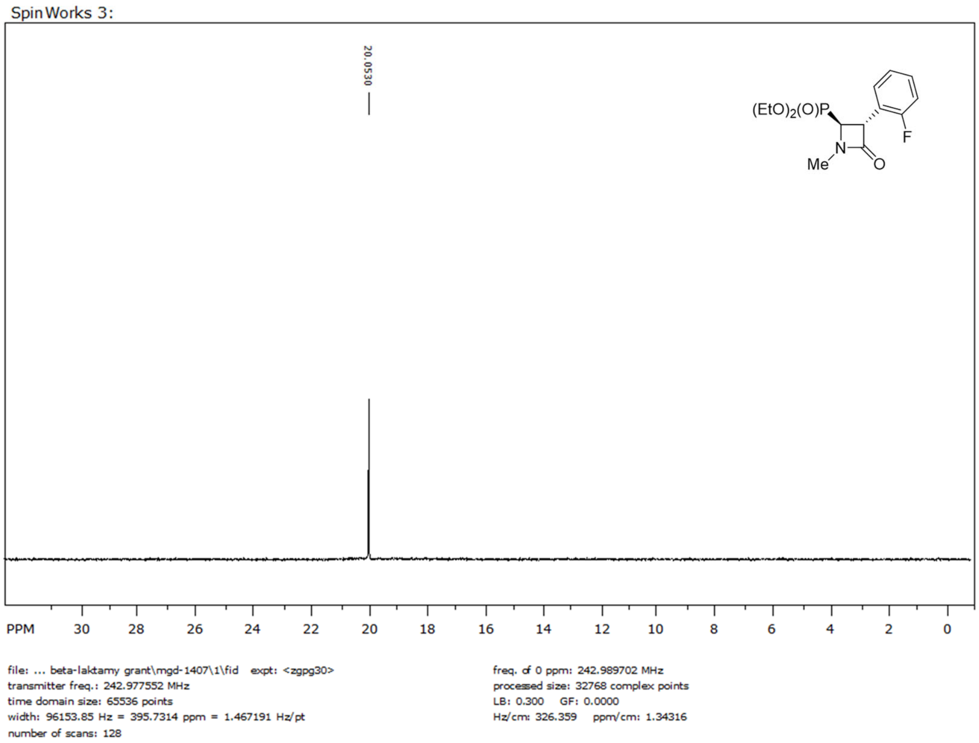

Figure S6: ^{31}P NMR Spectrum for *trans*-**10b** in $CDCl_3$

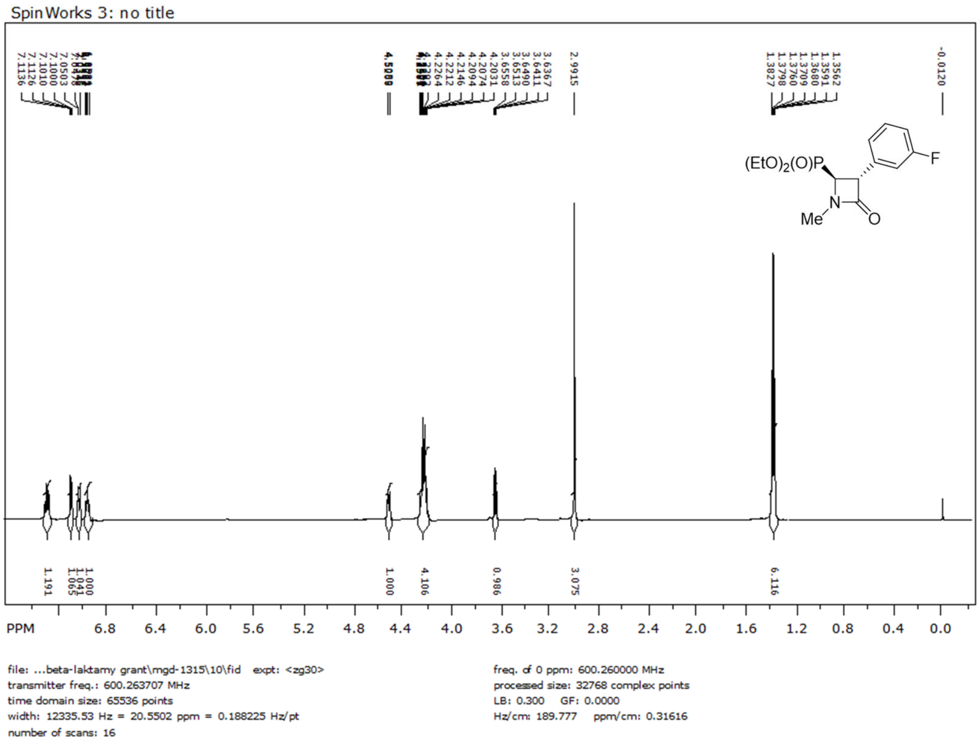

Figure S7: ^{1}H NMR Spectrum for *trans*-**10c** in $CDCl_3$

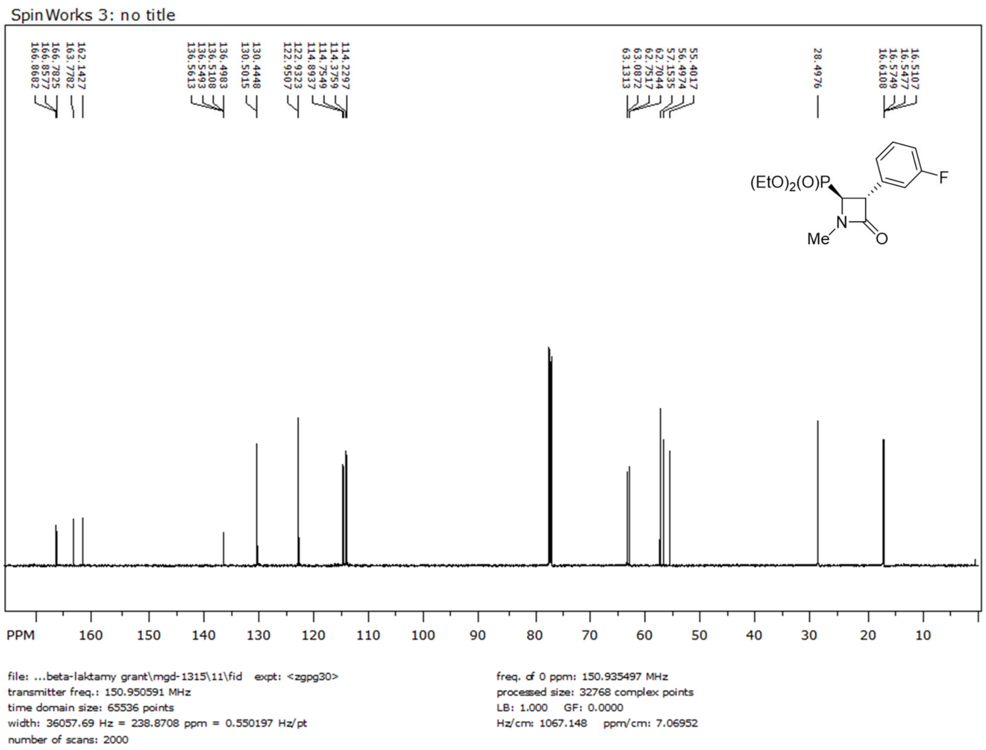

Figure S8: ^{13}C NMR Spectrum for *trans*-**10c** in $CDCl_3$

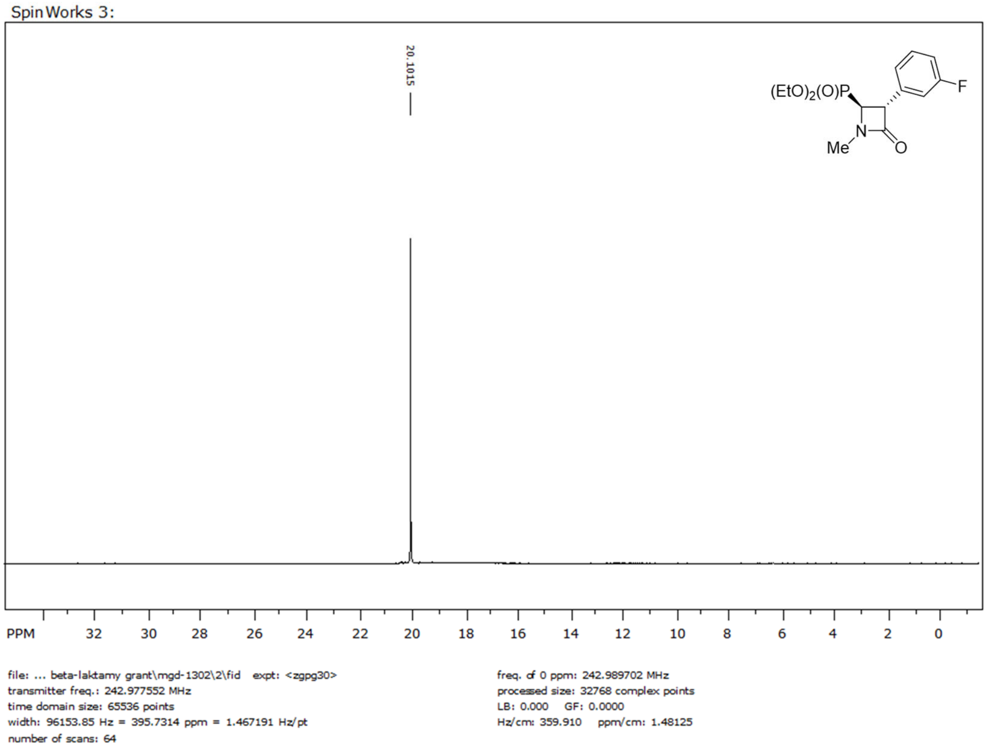

Figure S9: ^{31}P NMR Spectrum for *trans*-**10c** in $CDCl_3$

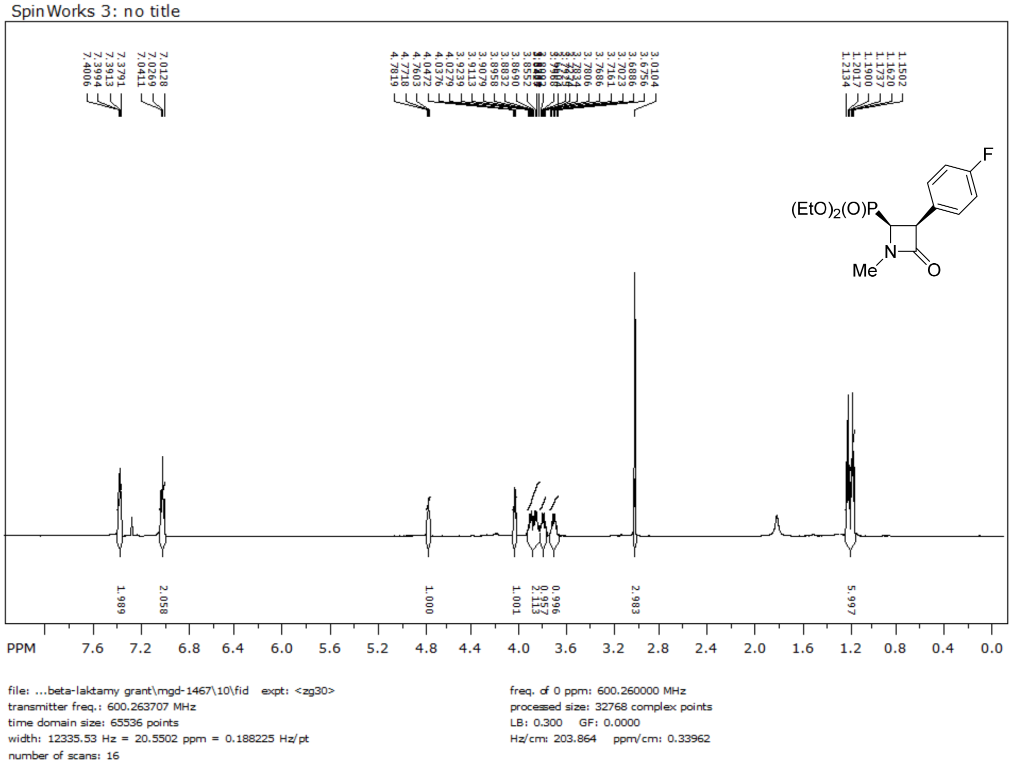

Figure S10: ^{1}H NMR Spectrum for *cis*-**10d** in $CDCl_3$

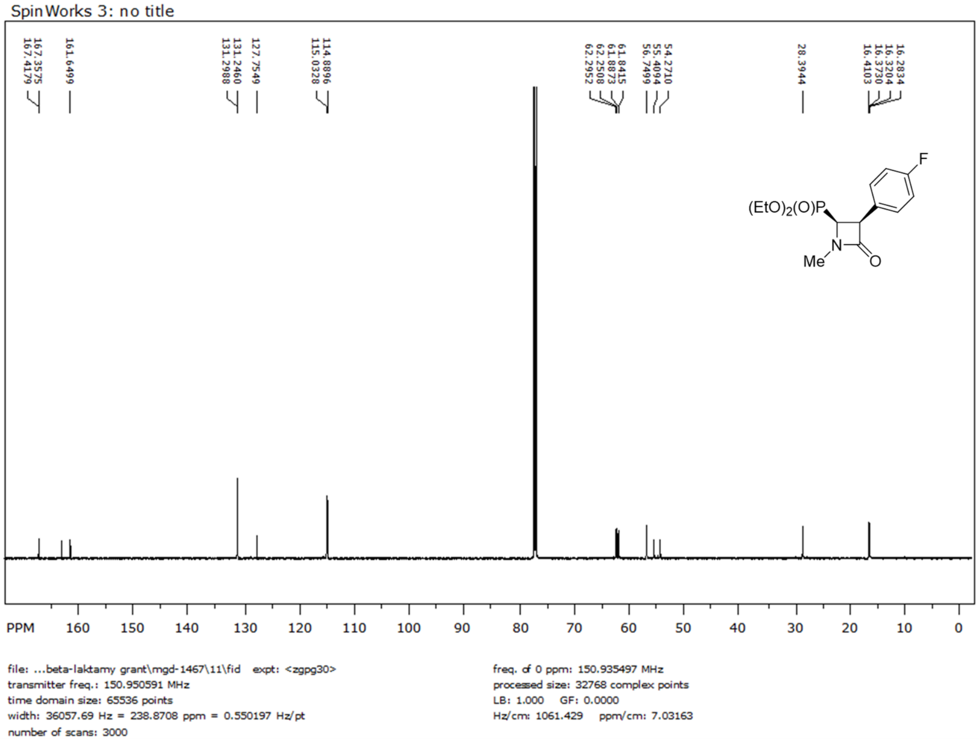

Figure S11: ^{13}C NMR Spectrum for *cis*-**10d** in $CDCl_3$

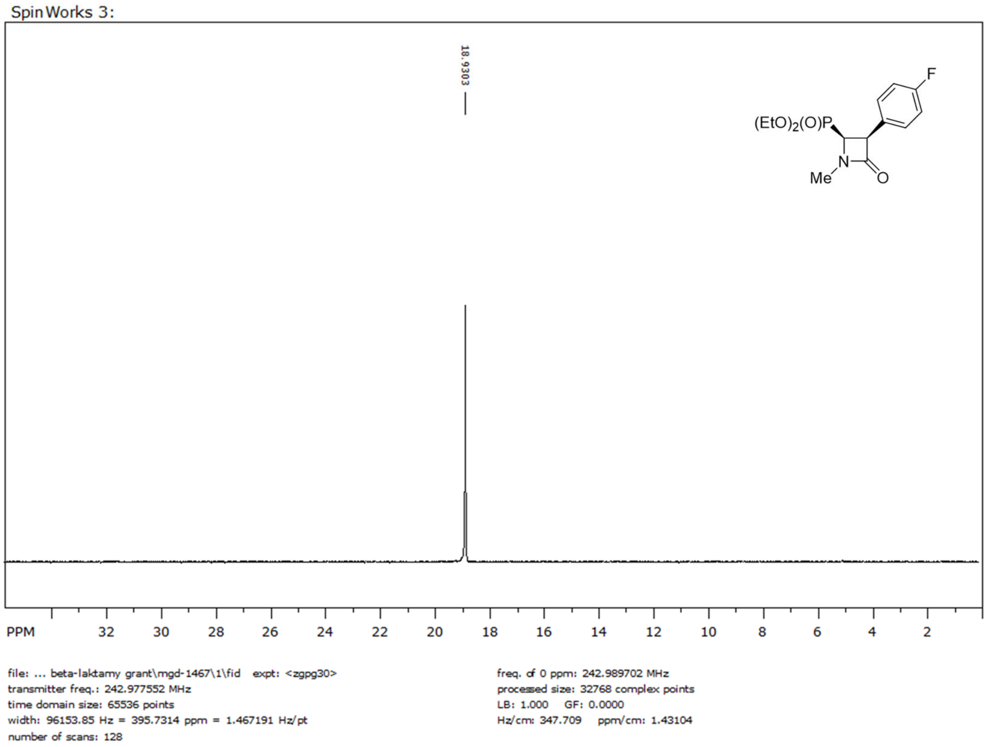

Figure S12: ^{31}P NMR Spectrum for *cis*-**10d** in $CDCl_3$

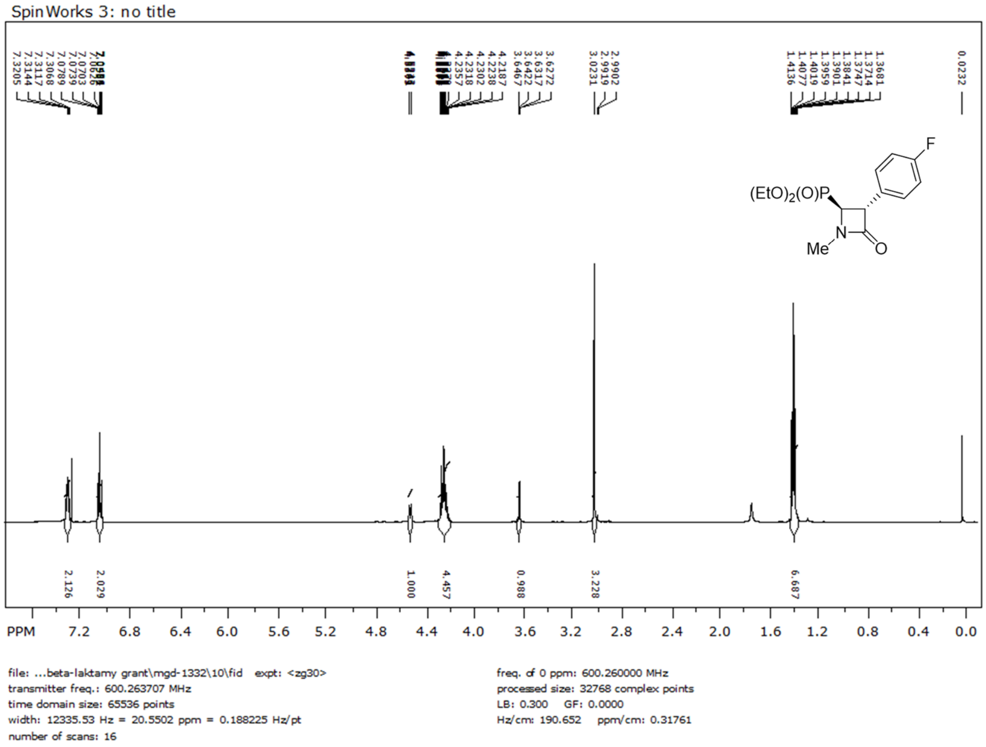

Figure S13: ^{1}H NMR Spectrum for *trans*-**10d** in $CDCl_3$

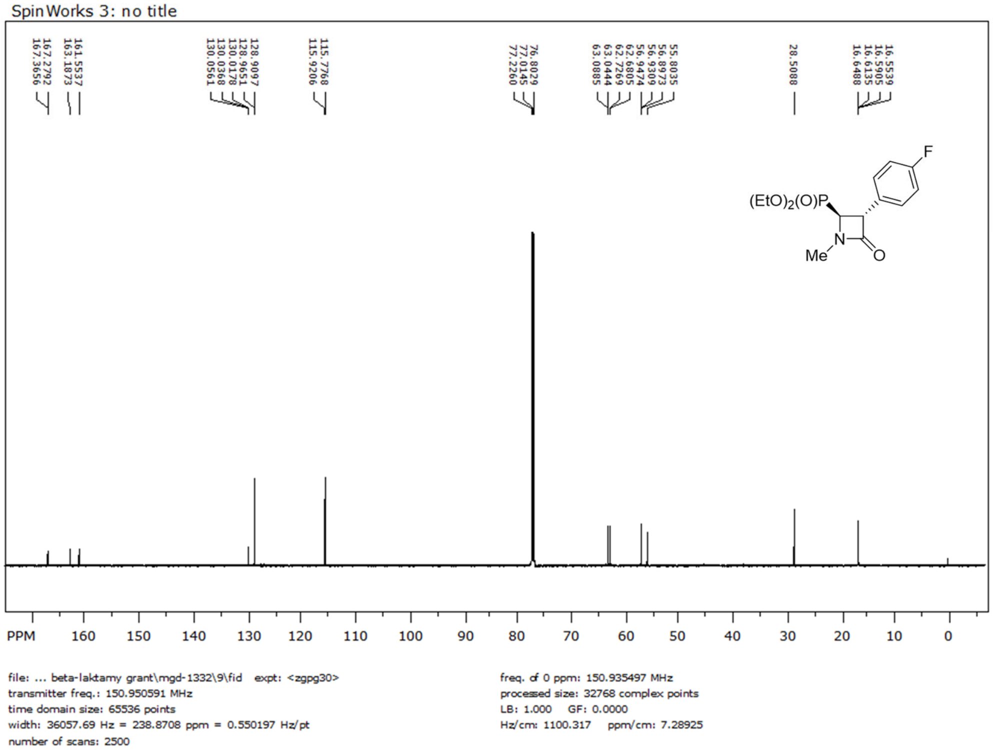

Figure S14: ^{13}C NMR Spectrum for *trans*-**10d** in $CDCl_3$

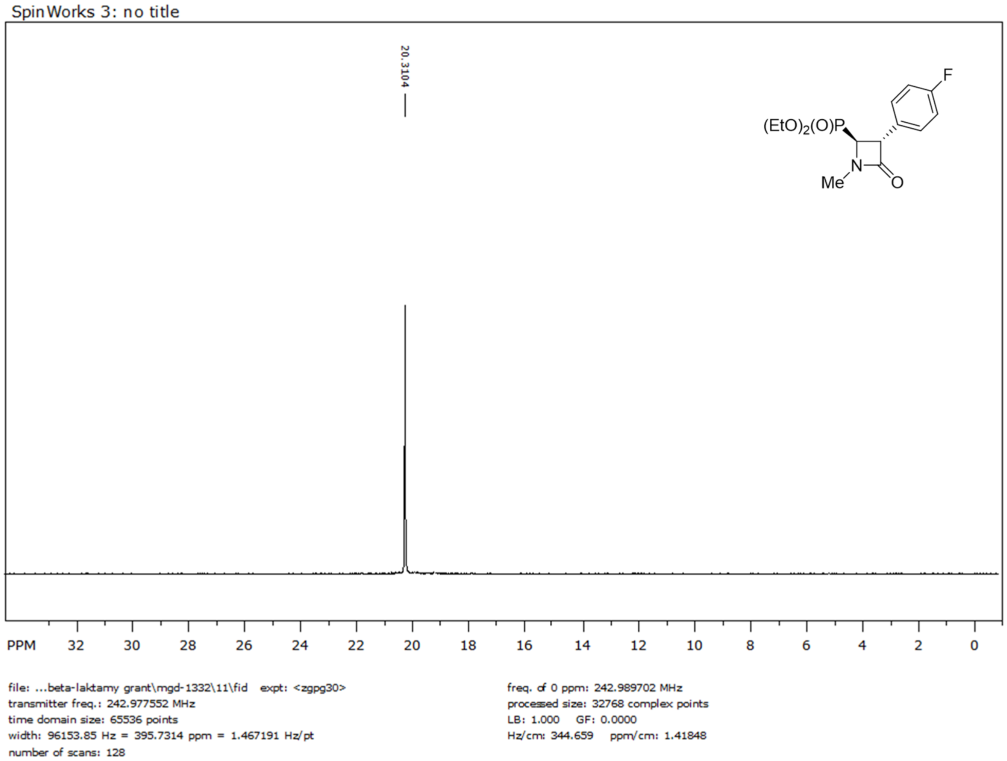

Figure S15: ^{31}P NMR Spectrum for *trans*-**10d** in $CDCl_3$

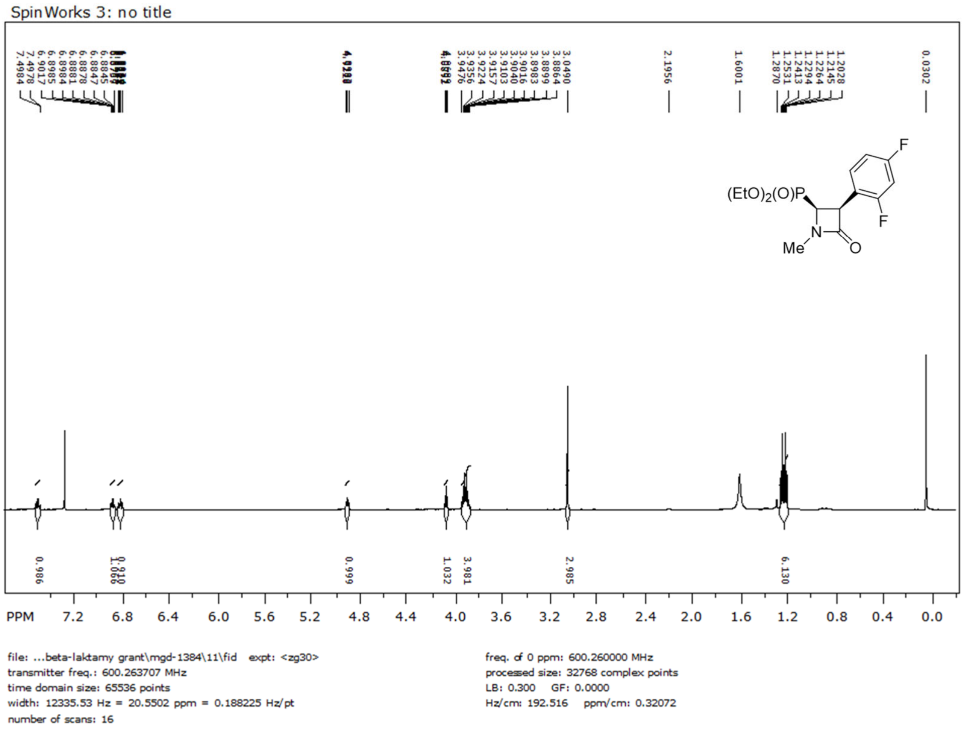

Figure S16: ^{1}H NMR Spectrum for *cis*-**10e** in $CDCl_3$

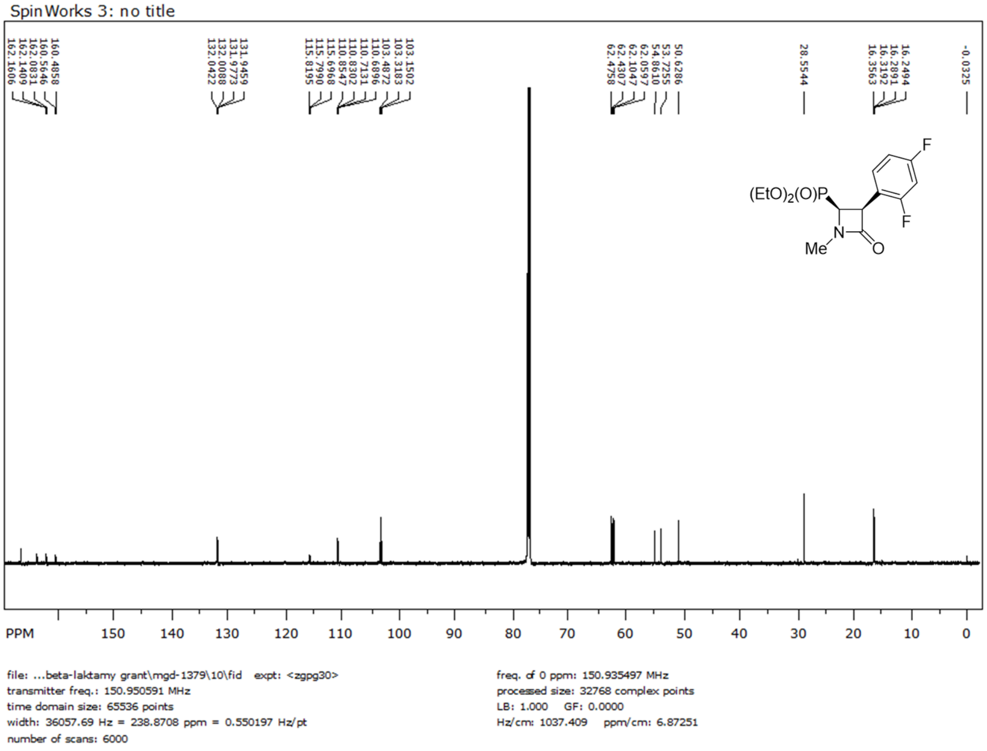

Figure S17: ^{13}C NMR Spectrum for *cis*-**10e** in $CDCl_3$

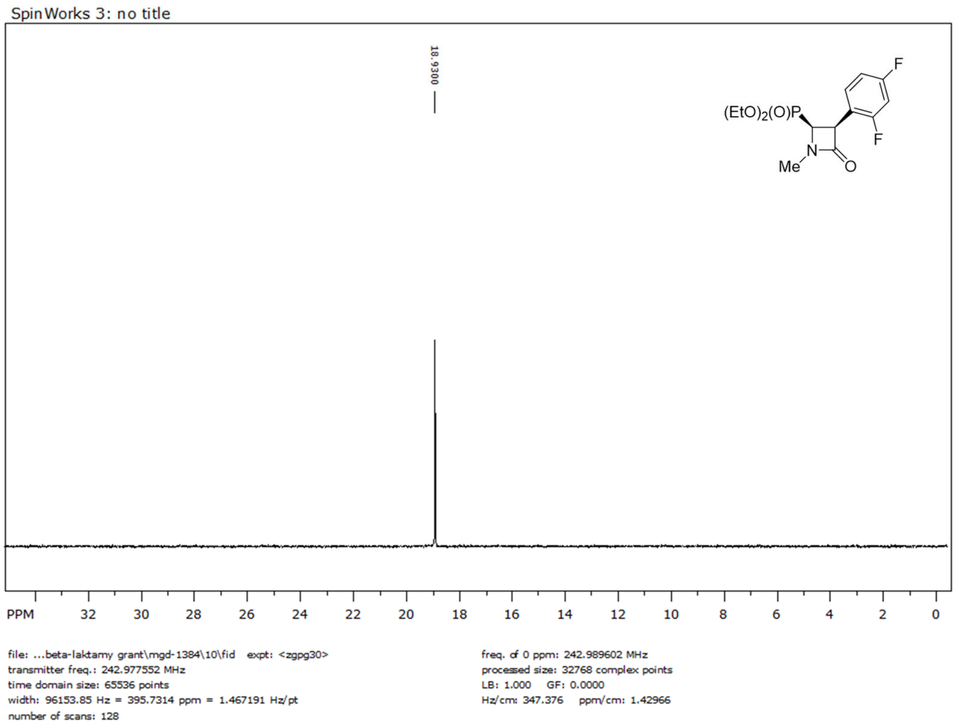

Figure S18: ^{31}P NMR Spectrum for *cis*-**10e** in $CDCl_3$

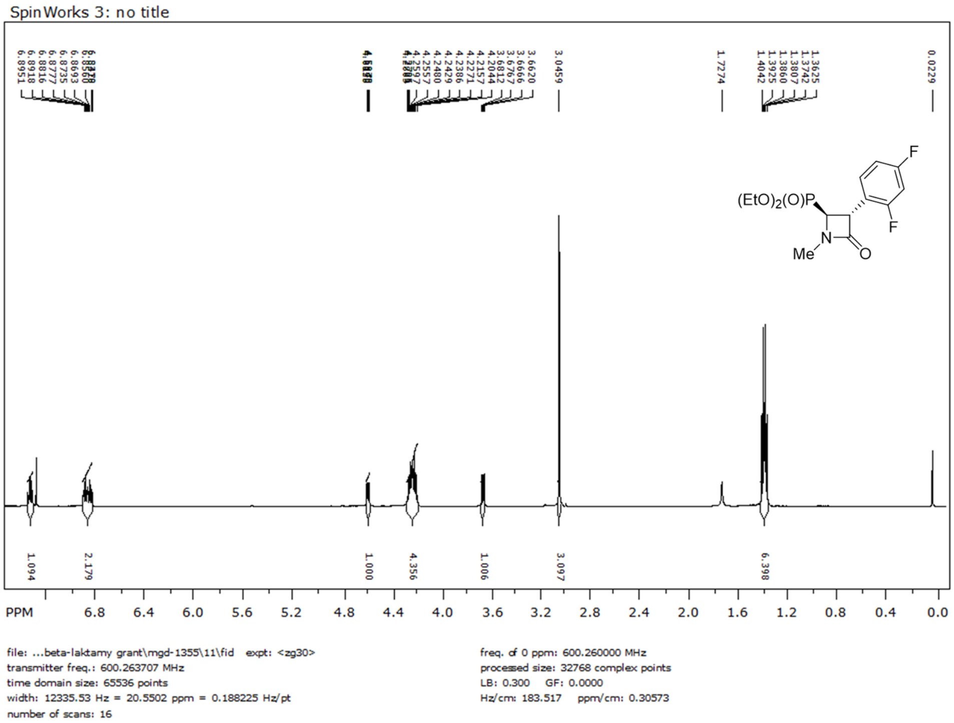

Figure S19: ^{1}H NMR Spectrum for *trans*-**10e** in $CDCl_3$

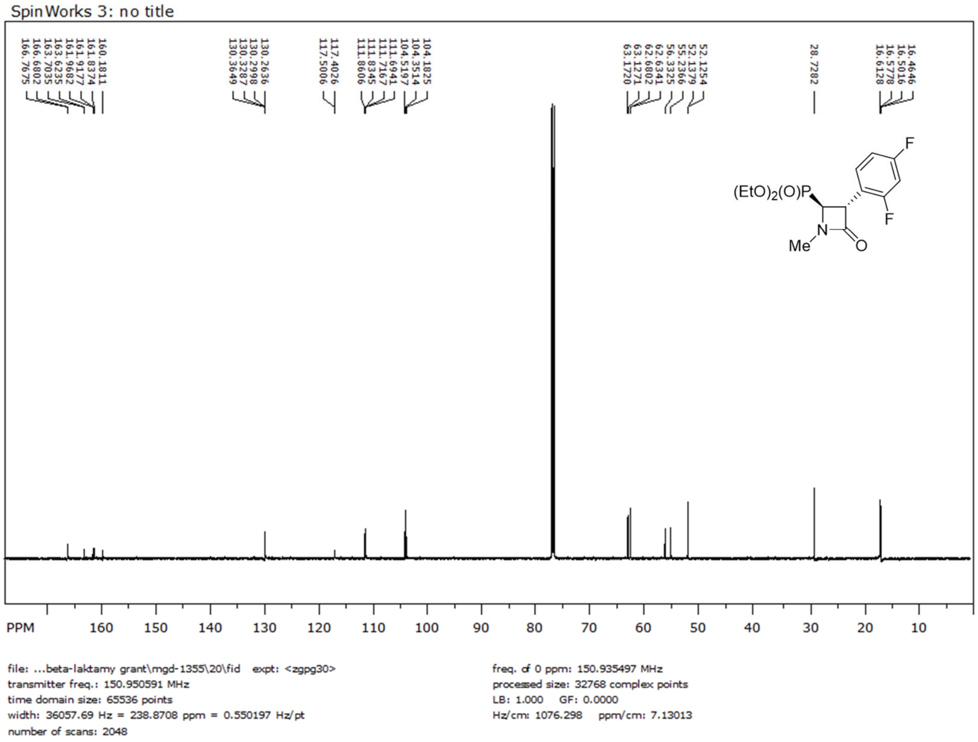

Figure S20: ^{13}C NMR Spectrum for *trans*-**10e** in $CDCl_3$

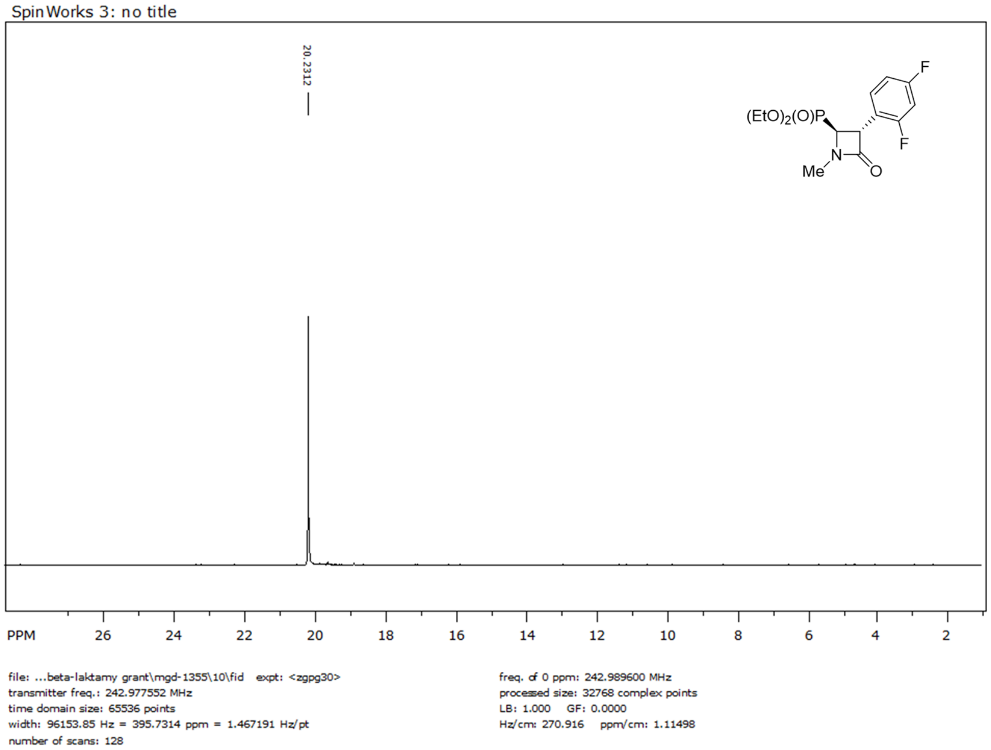

Figure S21: ^{31}P NMR Spectrum for *trans*-**10e** in $CDCl_3$

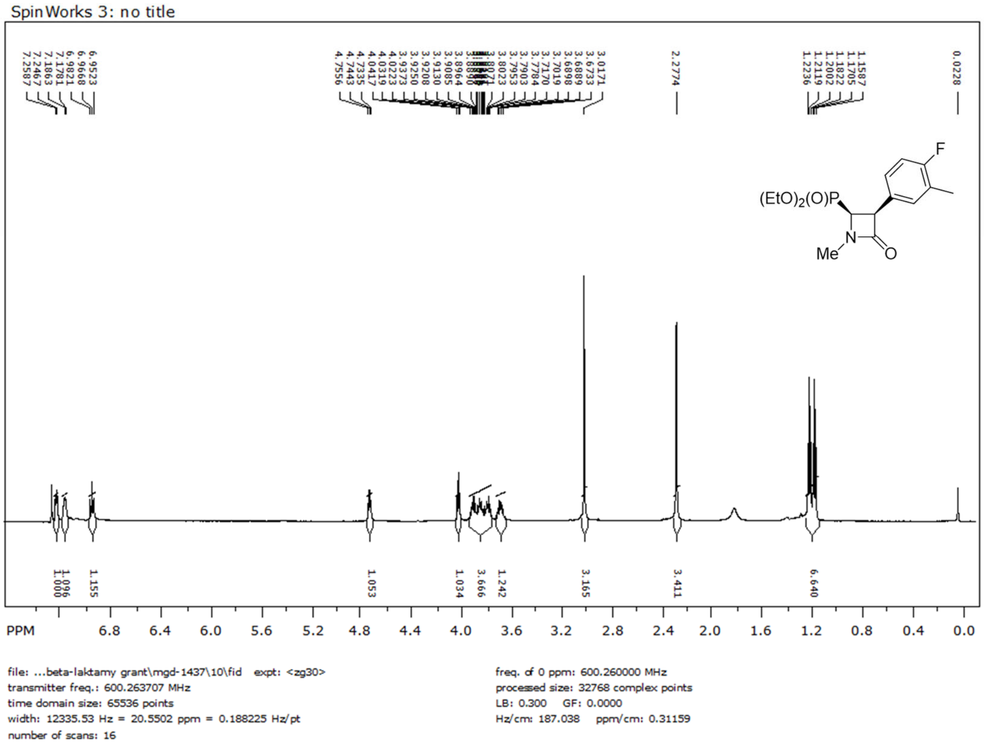

Figure S22: ^{1}H NMR Spectrum for *cis*-**10f** in $CDCl_3$

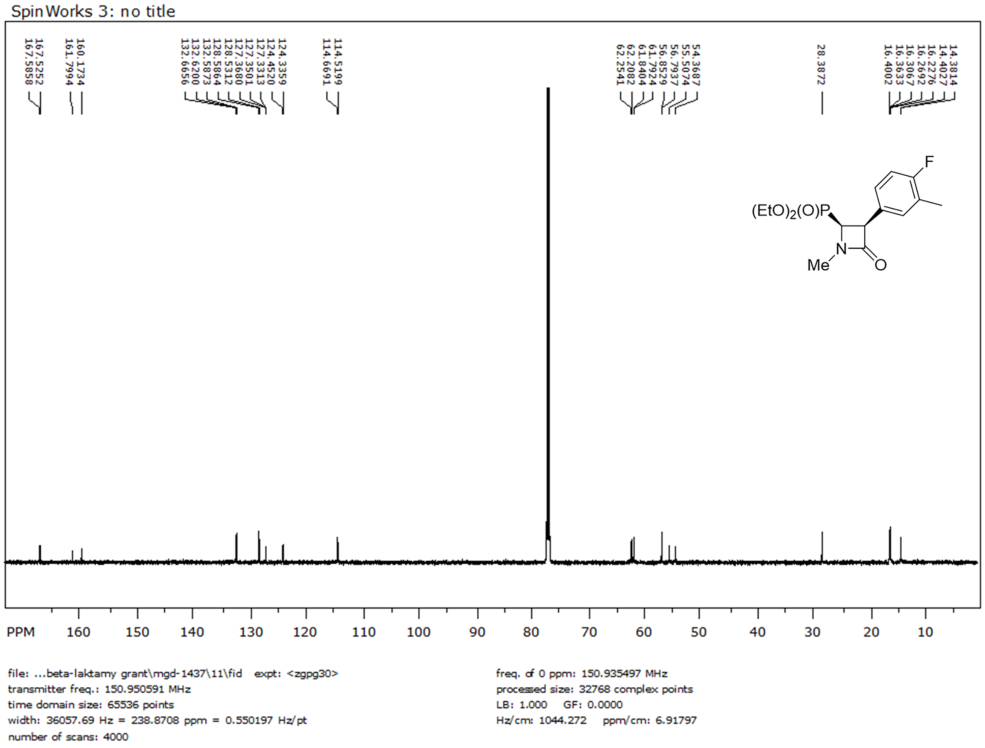

Figure S23: ^{13}C NMR Spectrum for *cis*-**10f** in $CDCl_3$

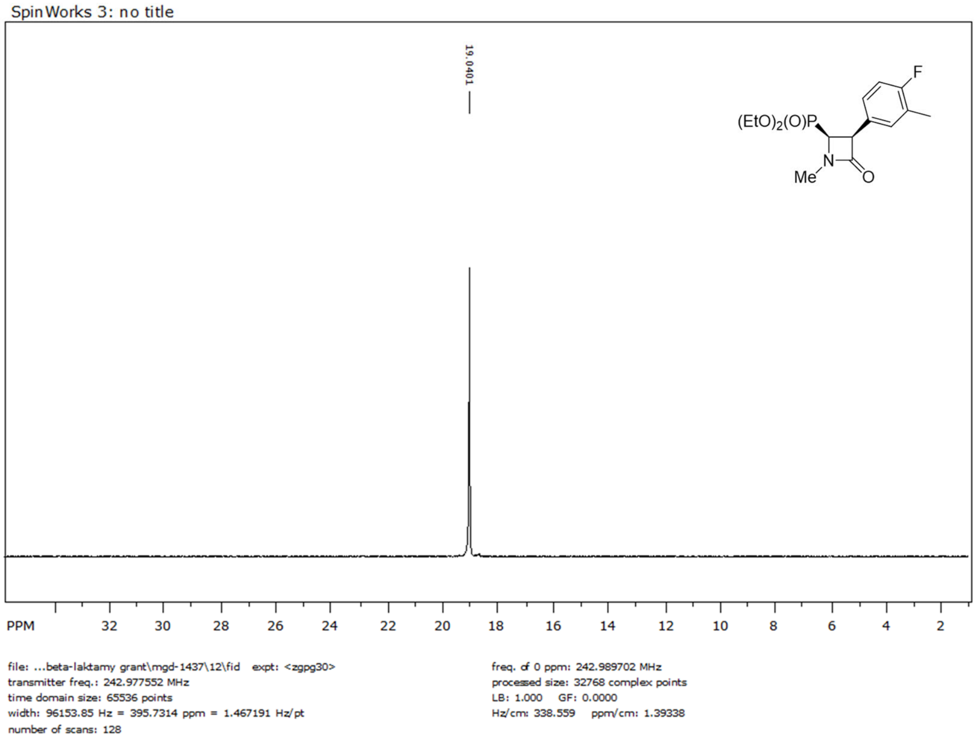

Figure S24: ^{31}P NMR Spectrum for *cis*-**10f** in $CDCl_3$

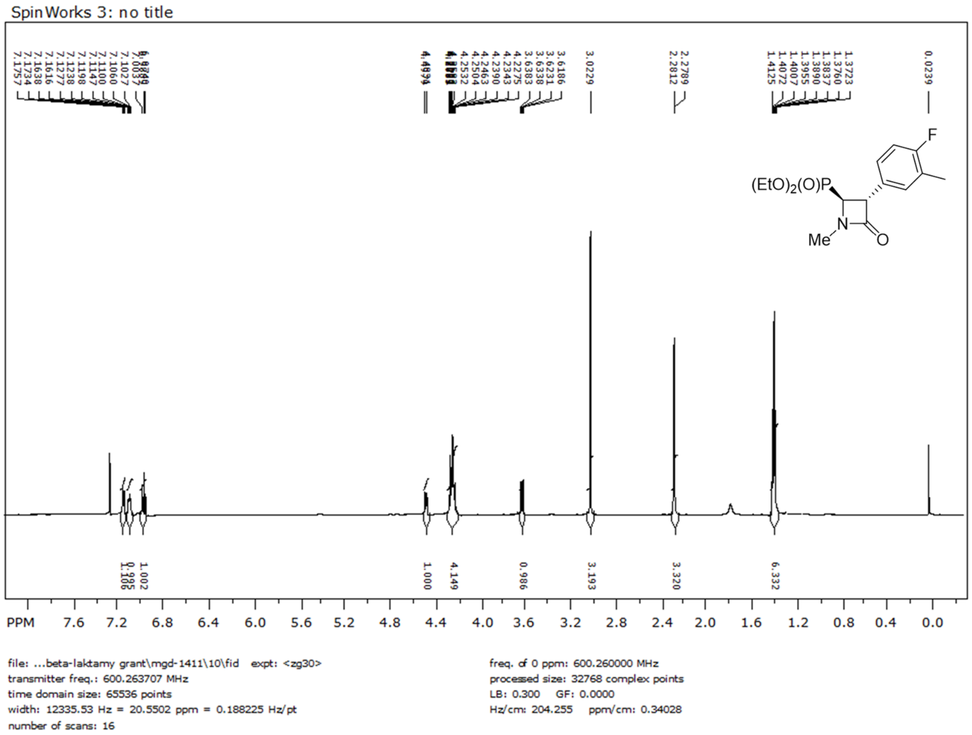

Figure S25: ^{1}H NMR Spectrum for *trans*-**10f** in $CDCl_3$

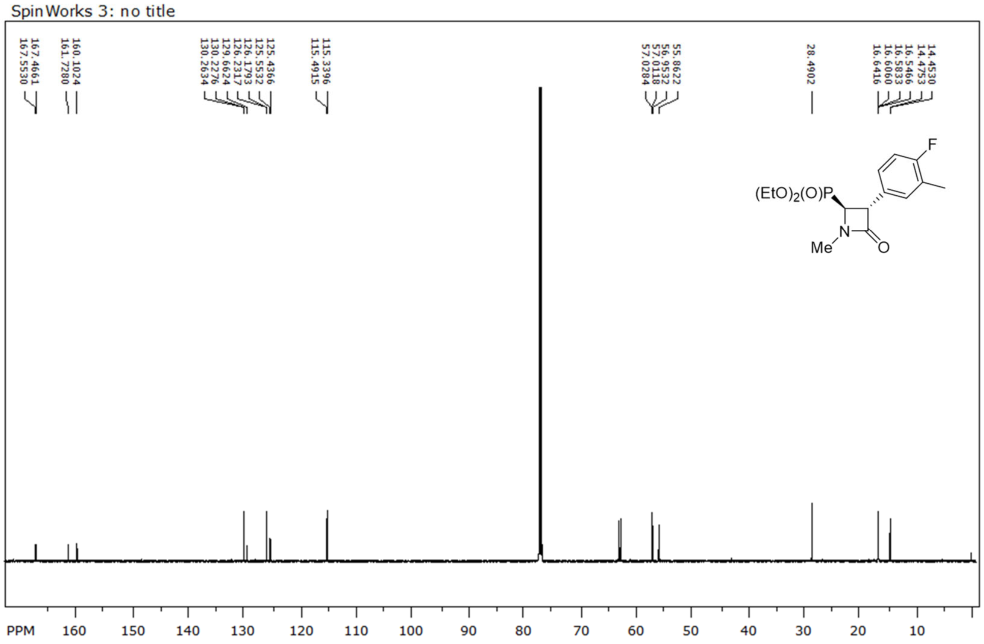

file: ...beta-laktamy grant\mgd-1411\11\fid expt: <zgpg30>
transmitter freq.: 150.950591 MHz
time domain size: 65536 points
width: 36057.69 Hz = 238.8708 ppm = 0.550197 Hz/pt
number of scans: 3000

freq. of 0 ppm: 150.935497 MHz
processed size: 32768 complex points
LB: 1.000 GF: 0.0000
Hz/cm: 1049.991 ppm/cm: 6.95586

Figure S26: ^{13}C NMR Spectrum for *trans*-**10f** in $CDCl_3$

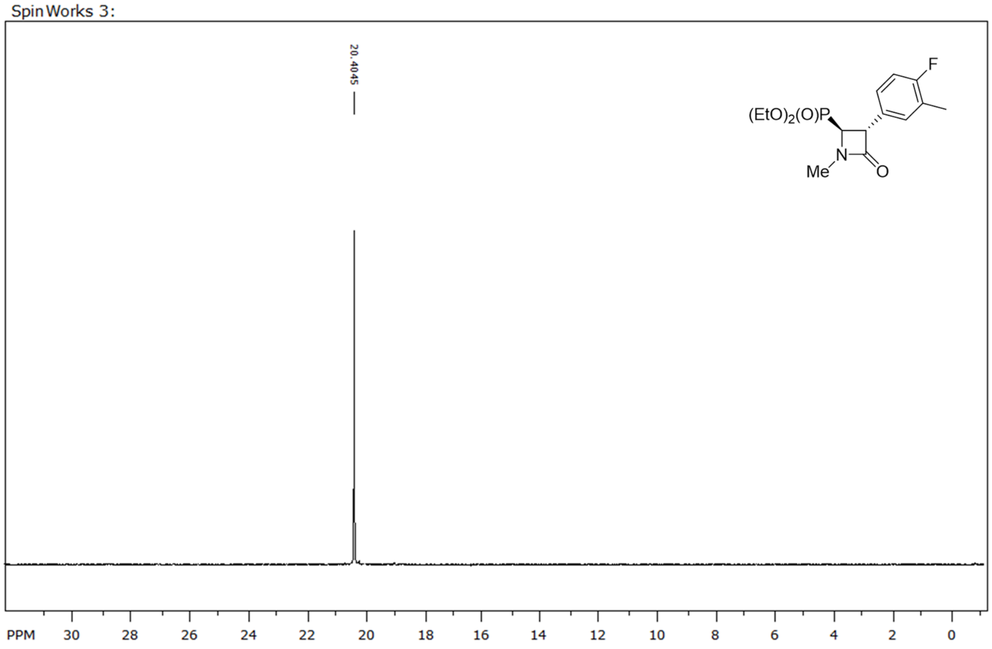

file: ... beta-laktamy grant\mgd-1411\1\fid expt: <zgpg30>
transmitter freq.: 242.977552 MHz
time domain size: 65536 points
width: 96153.85 Hz = 395.7314 ppm = 1.467191 Hz/pt
number of scans: 64

freq. of 0 ppm: 242.989702 MHz
processed size: 32768 complex points
LB: 0.300 GF: 0.0000
Hz/cm: 326.359 ppm/cm: 1.34316

Figure S27: ^{31}P NMR Spectrum for *trans*-**10f** in $CDCl_3$

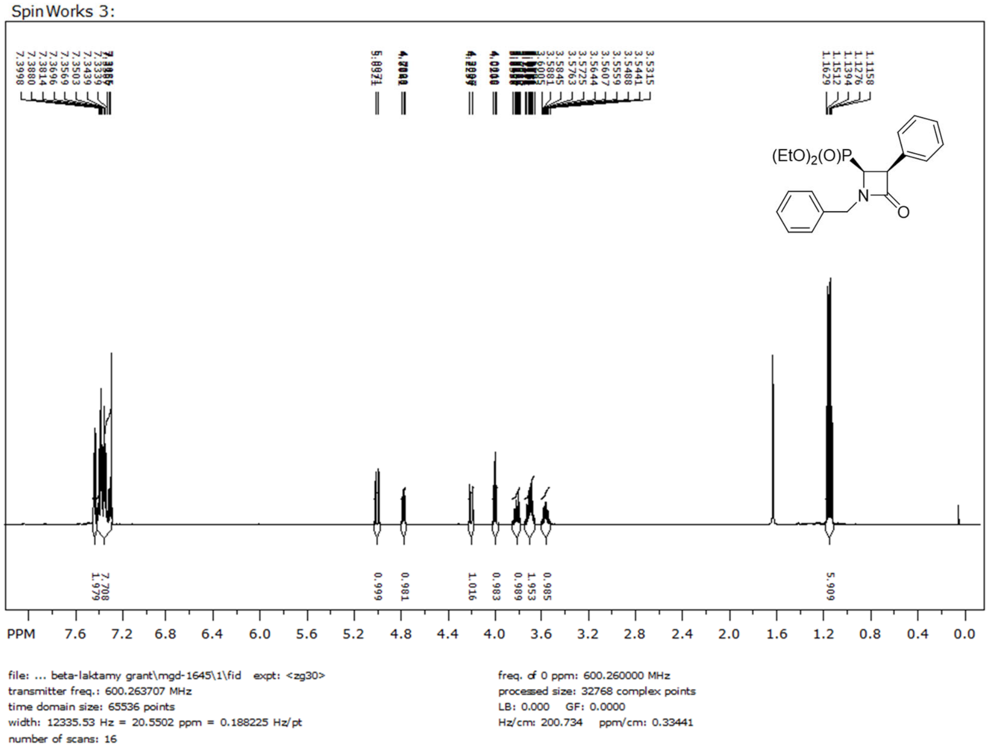

Figure S28: ^{1}H NMR Spectrum for *cis*-**11a** in $CDCl_3$

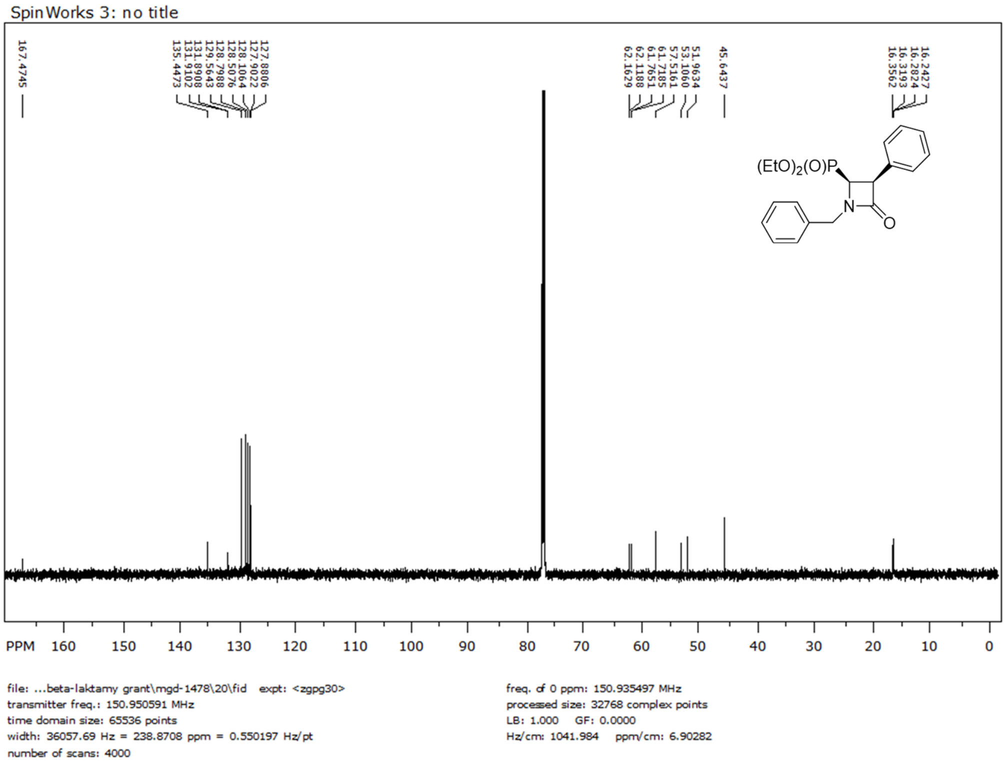

Figure S29: ^{13}C NMR Spectrum for *cis*-**11a** in $CDCl_3$

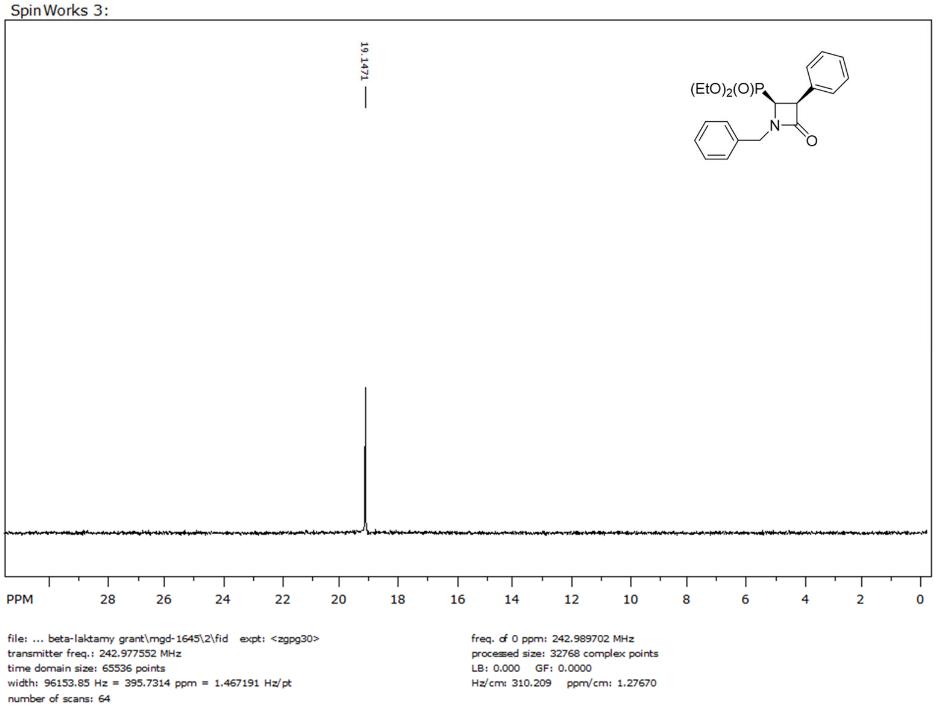

Figure S30: ^{31}P NMR Spectrum for *cis*-**11a** in $CDCl_3$

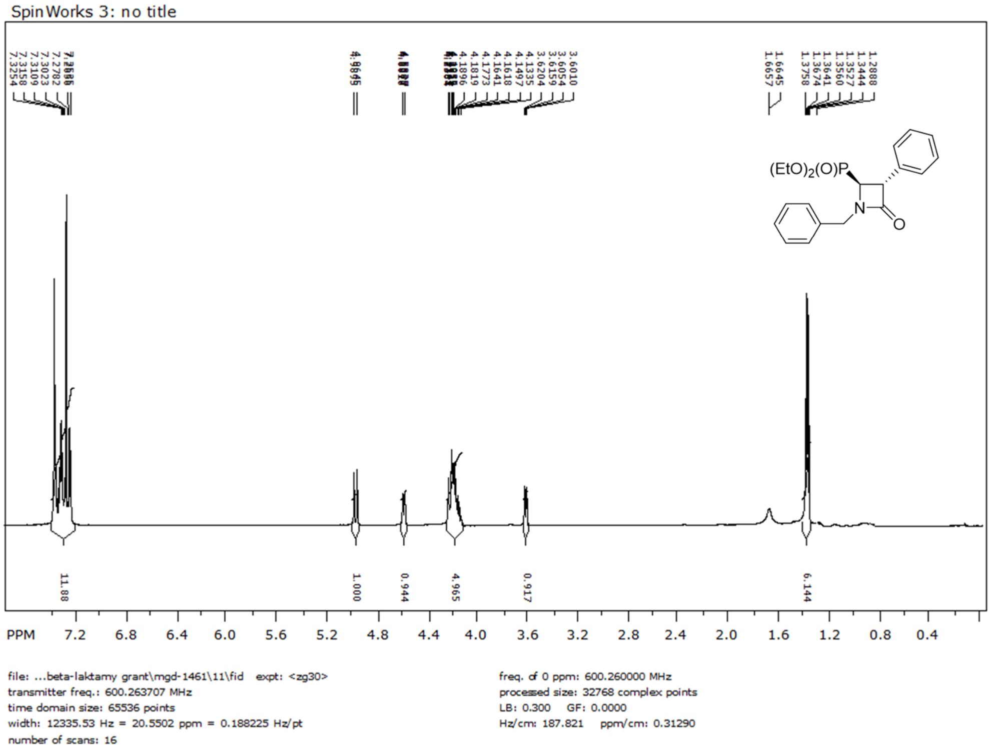

Figure S31: ^{1}H NMR Spectrum for *trans*-**11a** in $CDCl_3$

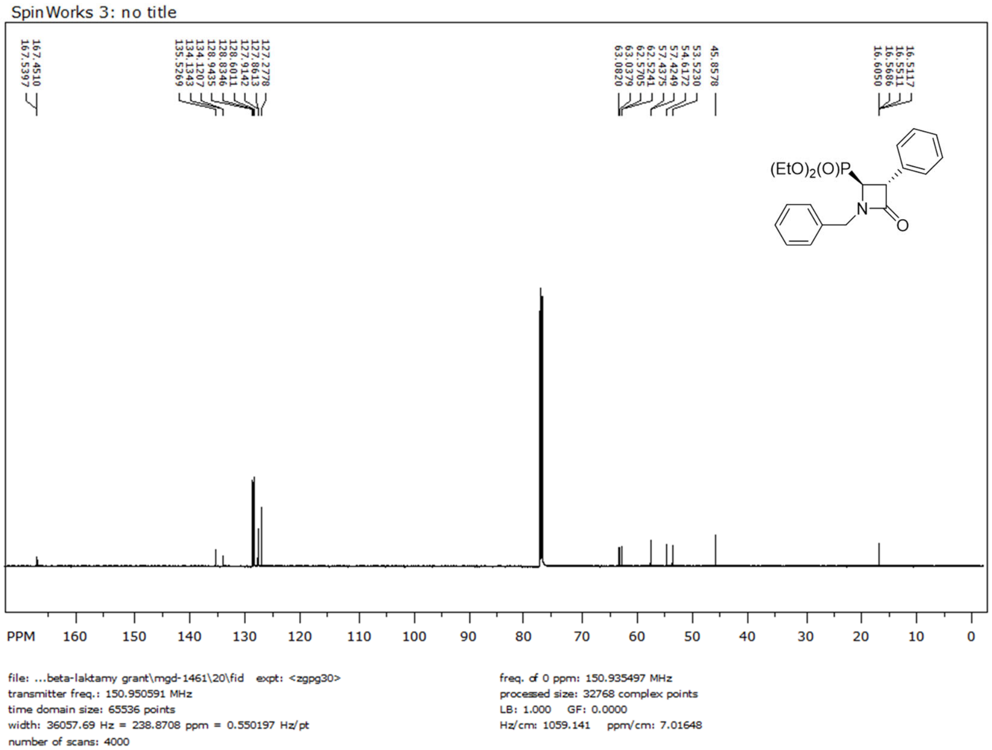

Figure S32: ^{13}C NMR Spectrum for *trans*-**11a** in $CDCl_3$

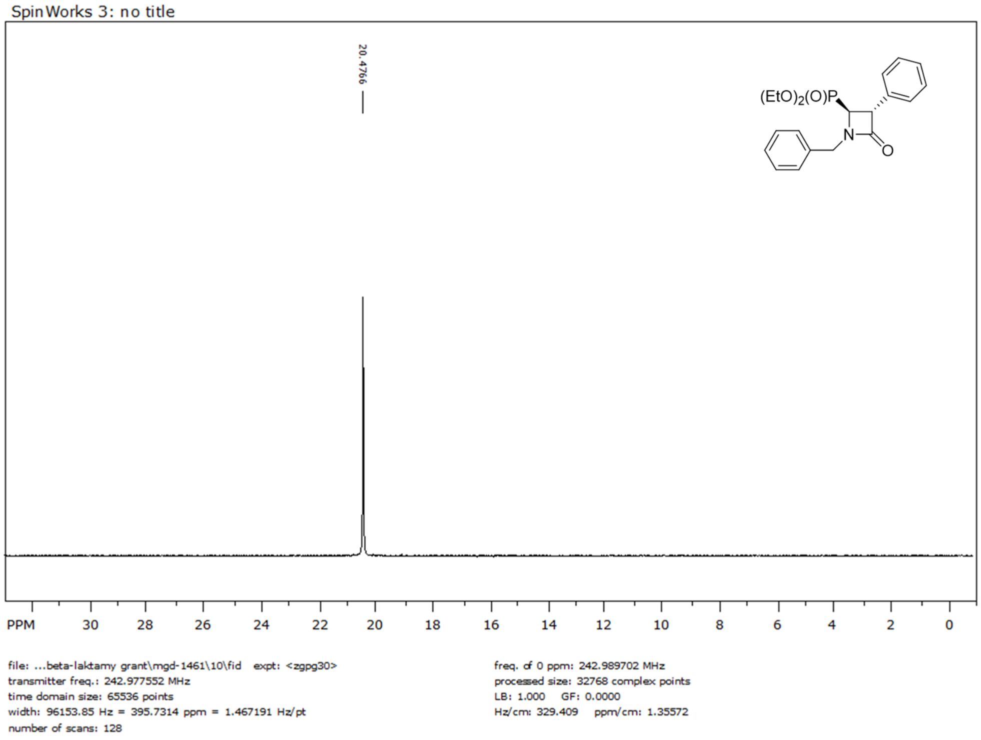

Figure S33: ^{31}P NMR Spectrum for *trans*-**11a** in $CDCl_3$

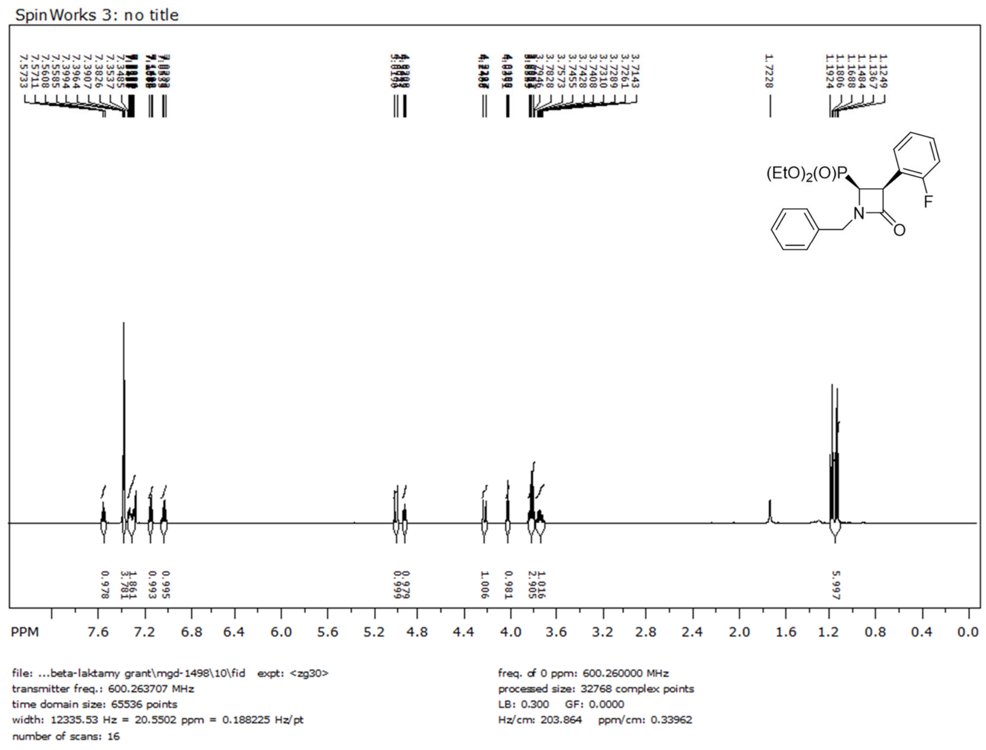

Figure S34: ^{1}H NMR Spectrum for *cis*-**11b** in $CDCl_3$

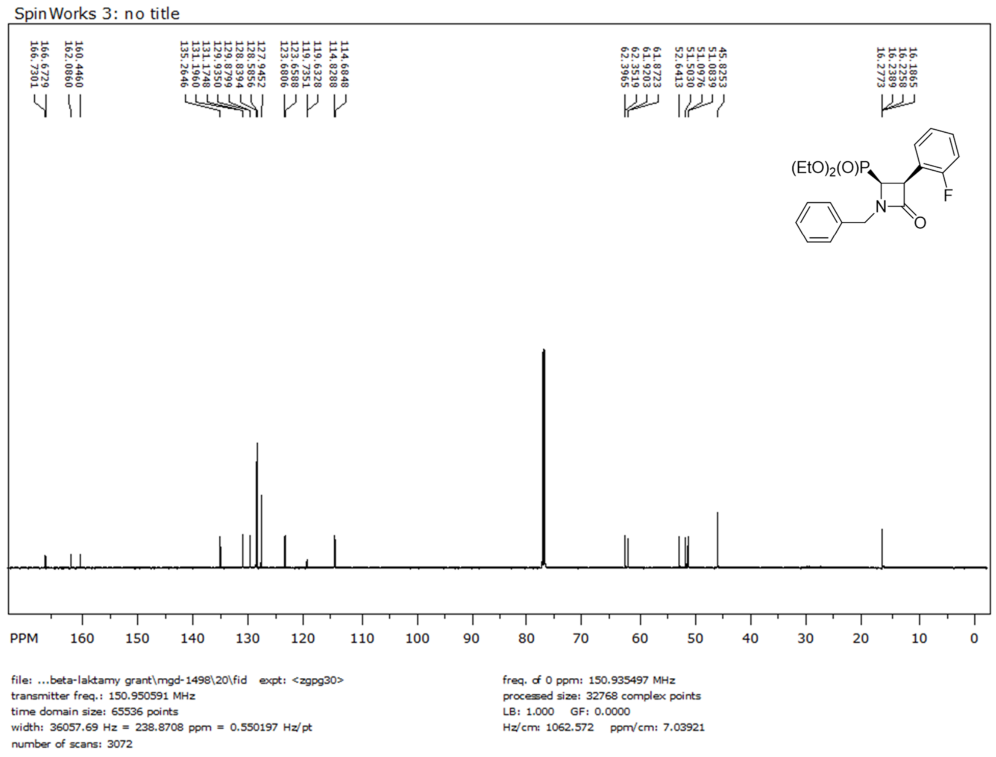

Figure S35: ^{13}C NMR Spectrum for *cis*-**11b** in $CDCl_3$

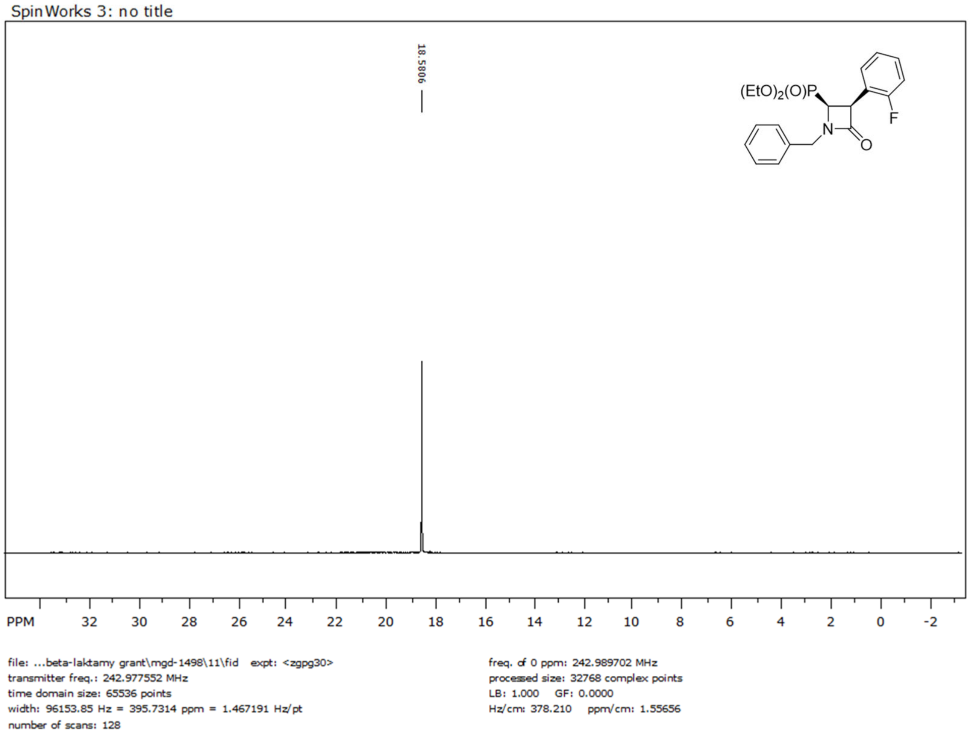

Figure S36: ^{31}P NMR Spectrum for *cis*-**11b** in $CDCl_3$

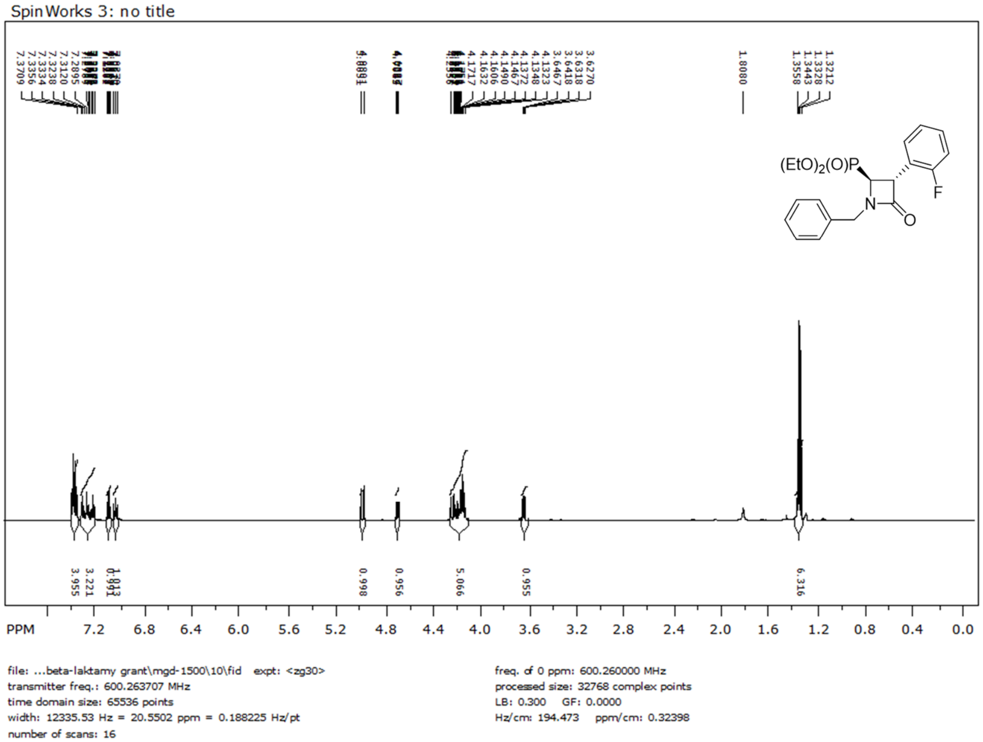

Figure S37: ^{1}H NMR Spectrum for *trans*-**11b** in $CDCl_3$

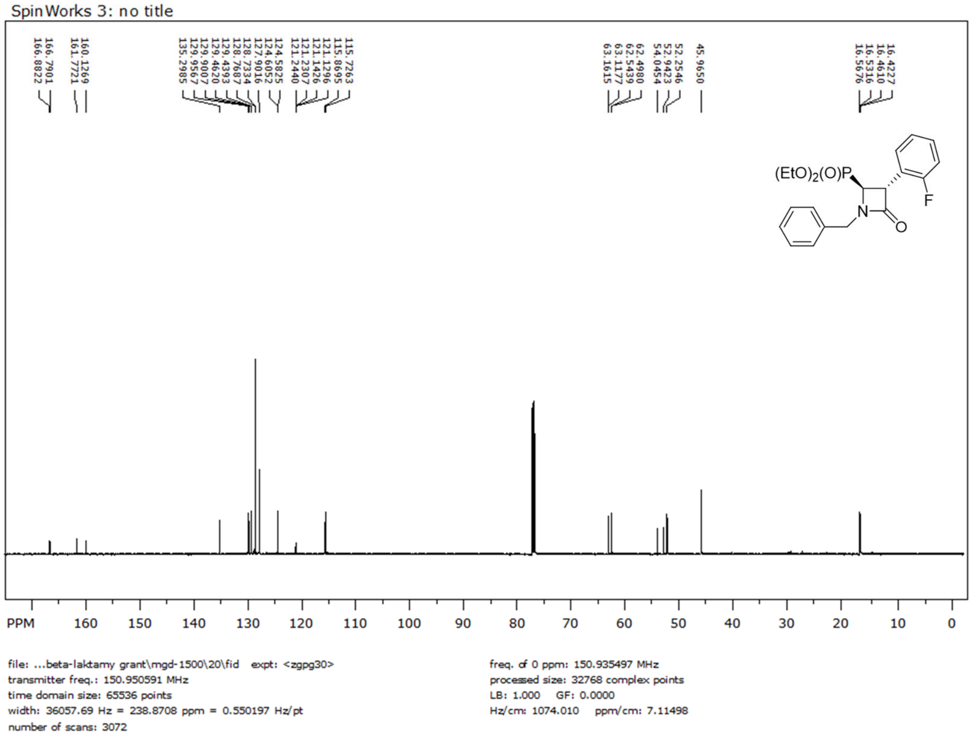

Figure S38: ^{13}C NMR Spectrum for *trans*-**11b** in $CDCl_3$

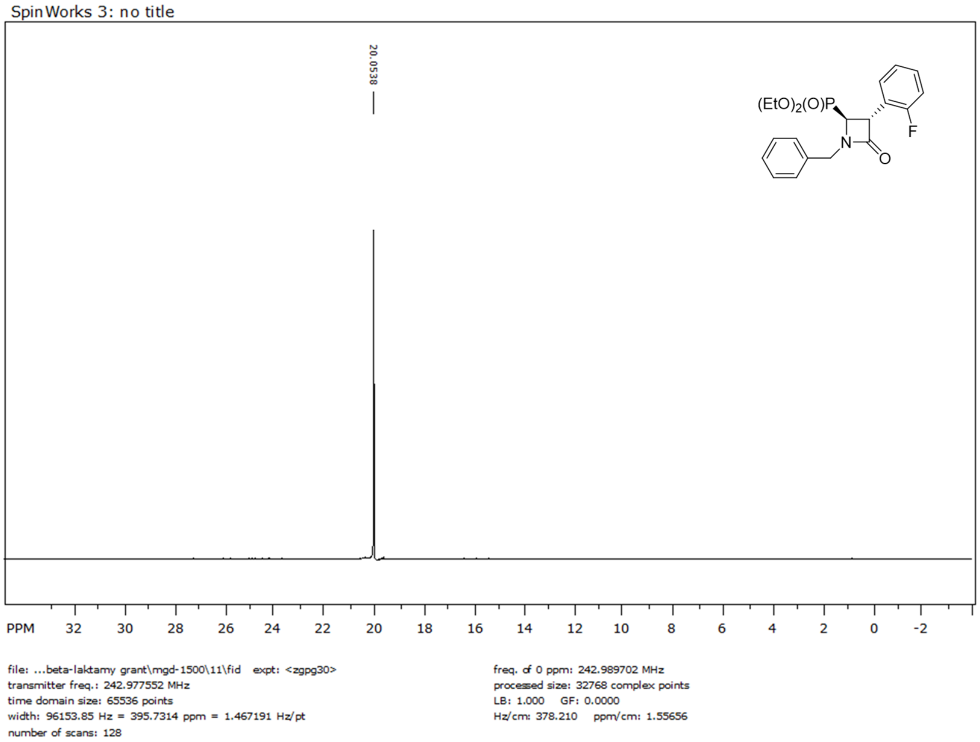

Figure S39: ^{31}P NMR Spectrum for *trans*-**11b** in $CDCl_3$

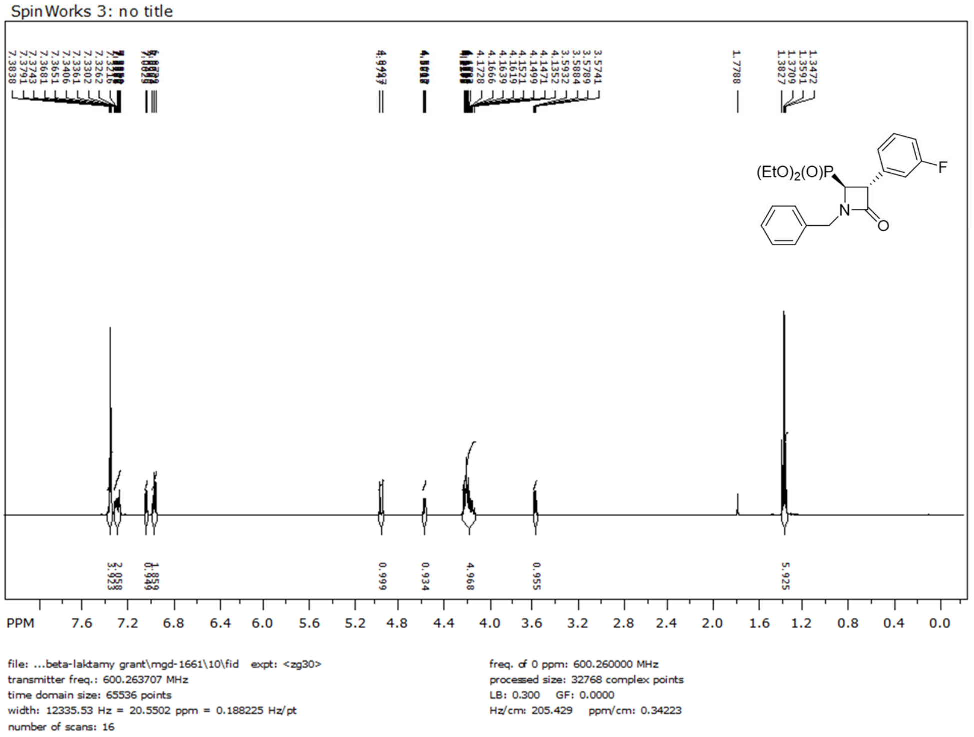

Figure S40: ^{1}H NMR Spectrum for *trans*-**11c** in $CDCl_3$

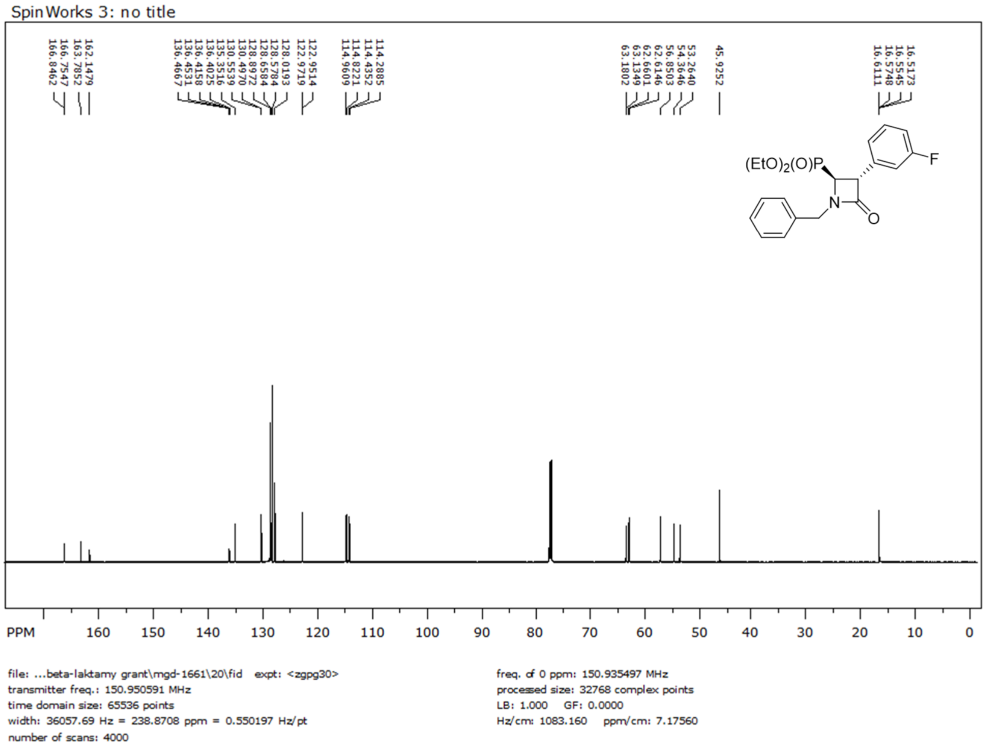

Figure S41: ^{13}C NMR Spectrum for *trans*-**11c** in $CDCl_3$

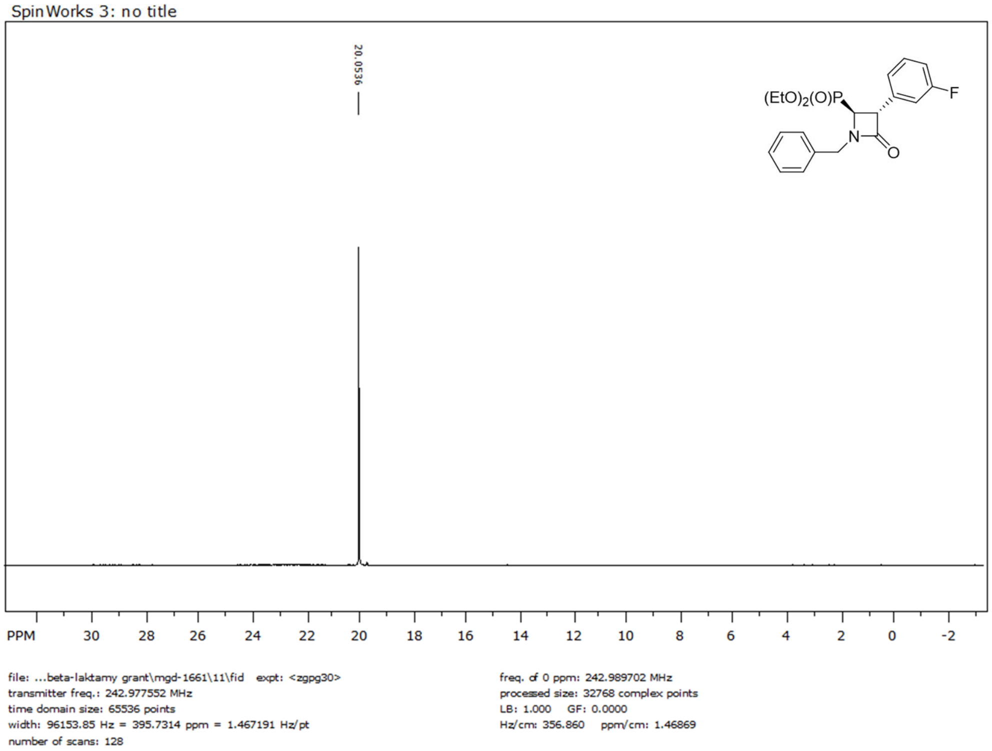

Figure S42: ^{31}P NMR Spectrum for *trans*-**11c** in $CDCl_3$

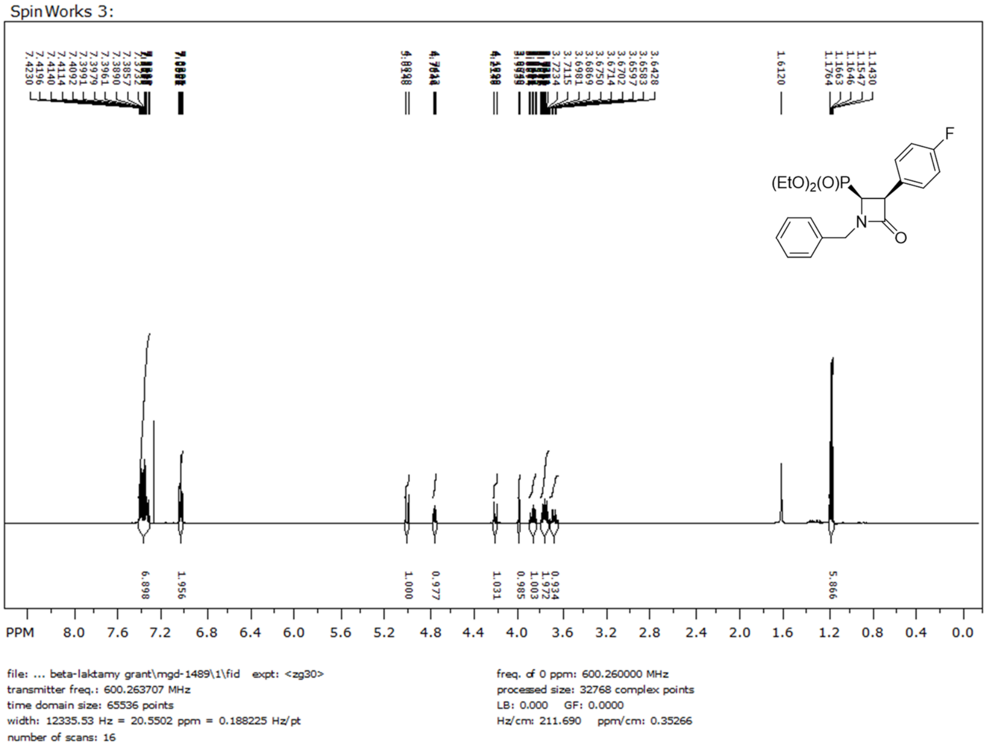

Figure S43: ^{1}H NMR Spectrum for *cis*-**11d** in $CDCl_3$

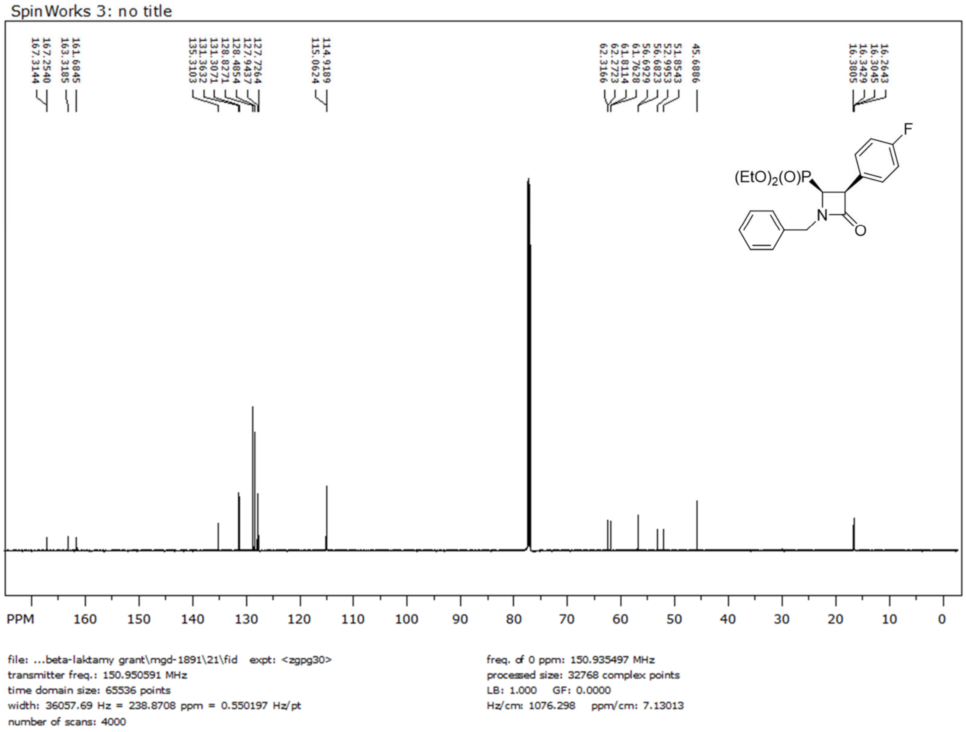

Figure S44: ^{13}C NMR Spectrum for *cis*-**11d** in $CDCl_3$

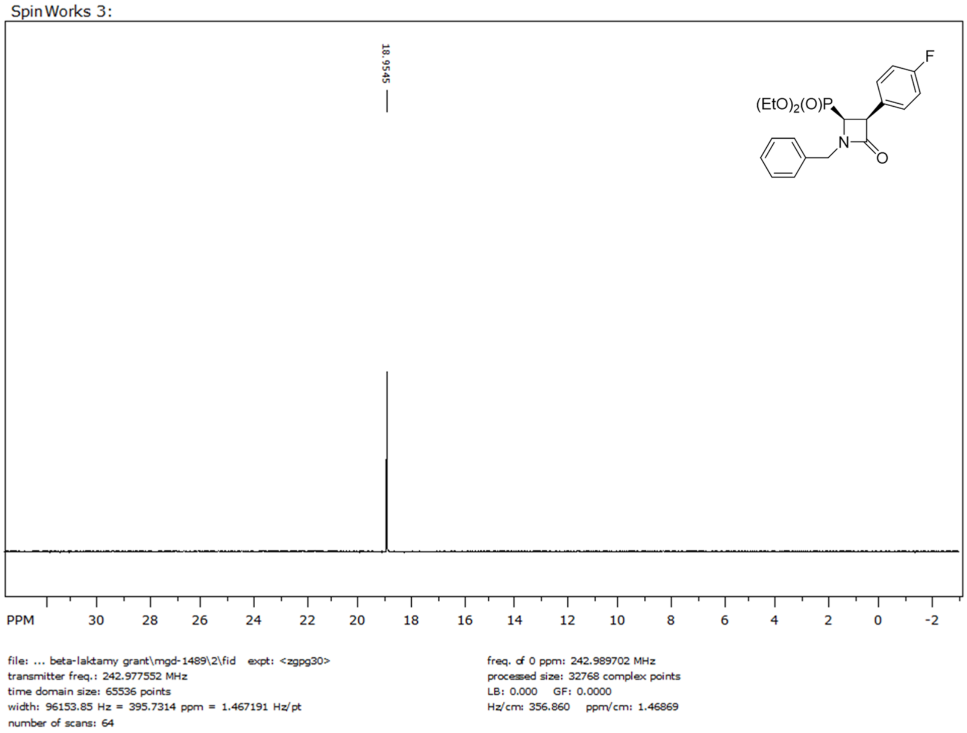

Figure S45: ^{31}P NMR Spectrum for *cis*-**11d** in $CDCl_3$

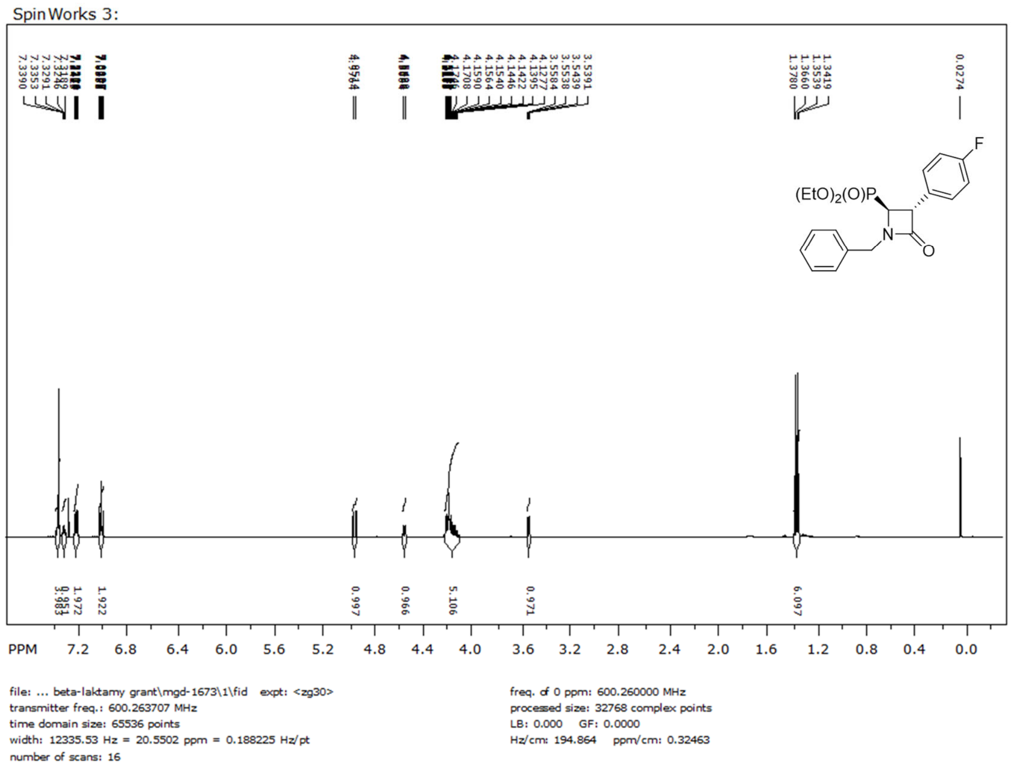

Figure S46: ^{1}H NMR Spectrum for *trans*-**11d** in $CDCl_3$

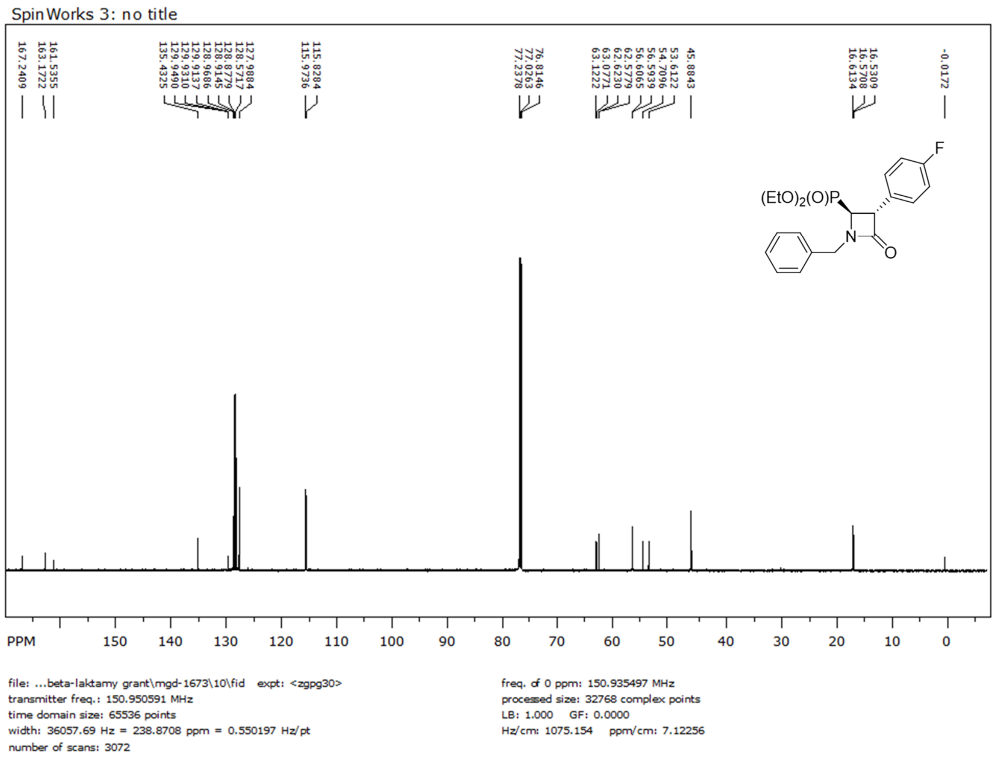

Figure S47: ^{13}C NMR Spectrum for *trans*-**11d** in $CDCl_3$

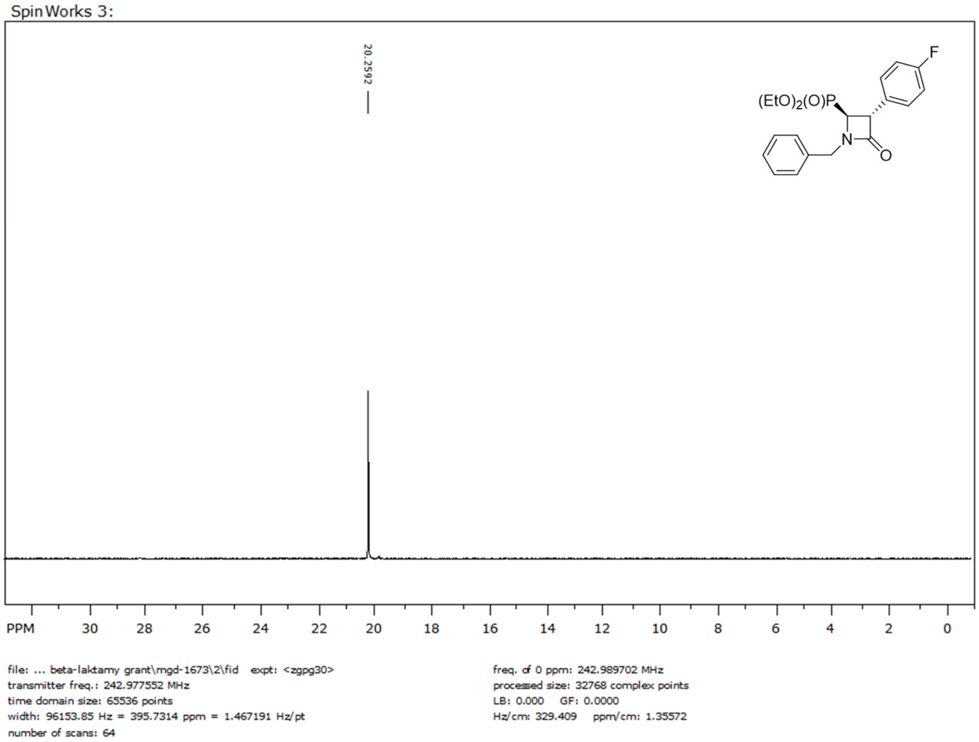

Figure S48: ^{31}P NMR Spectrum for *trans*-**11d** in $CDCl_3$

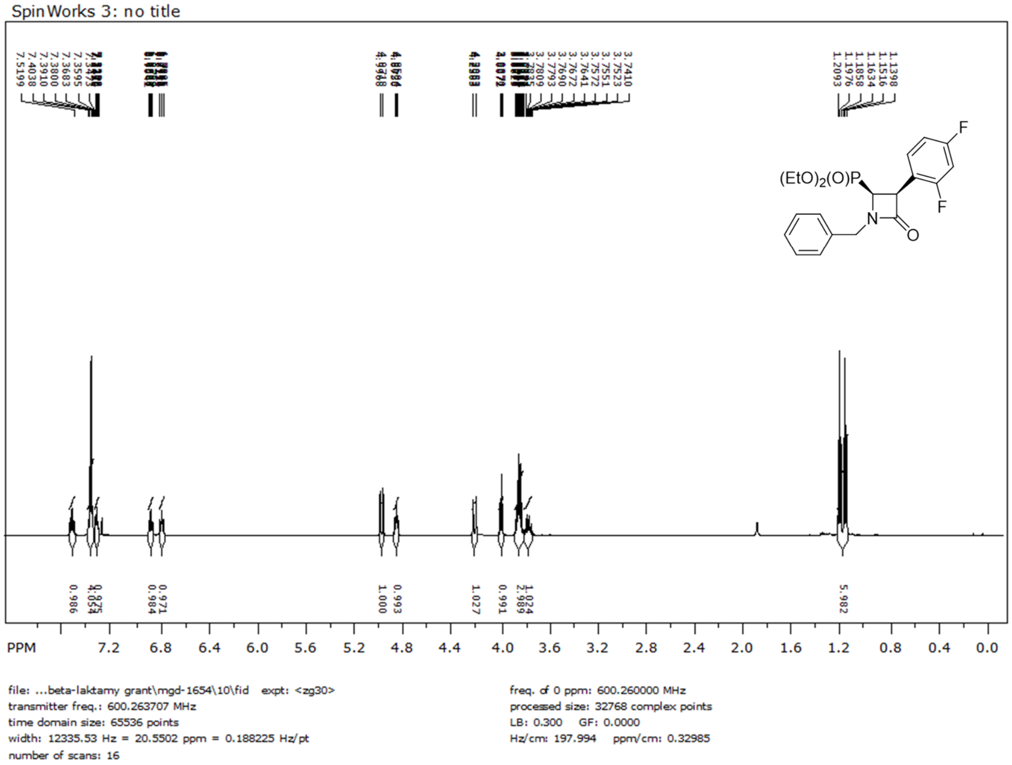

Figure S49: ^{1}H NMR Spectrum for *cis*-**11e** in $CDCl_3$

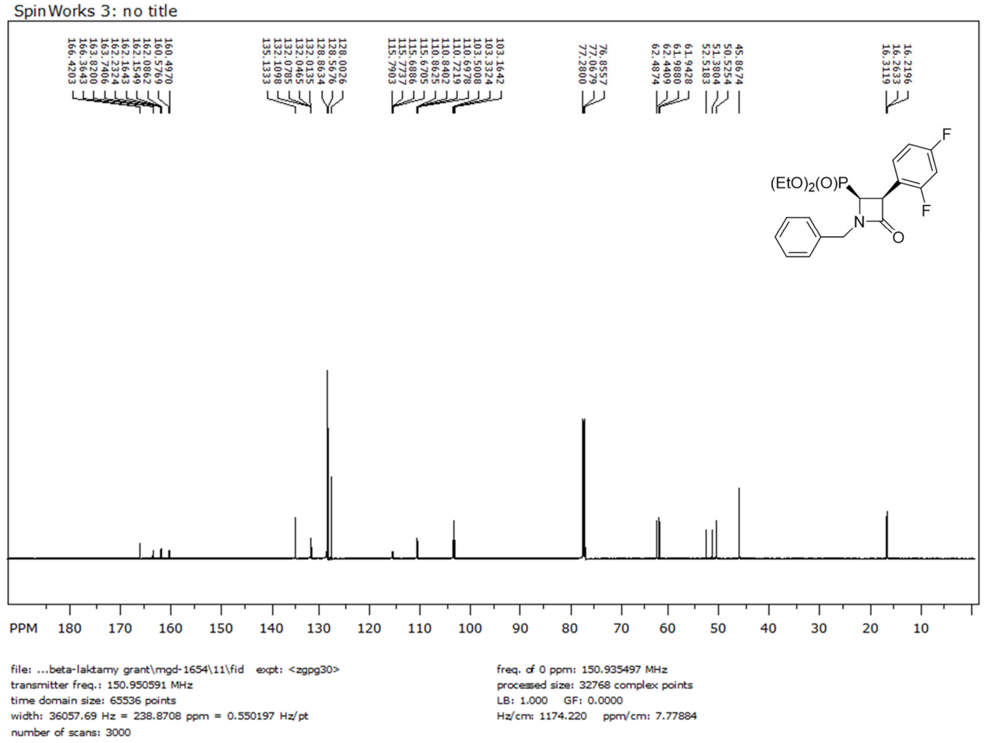

Figure S50: ^{13}C NMR Spectrum for *cis*-**11e** in $CDCl_3$

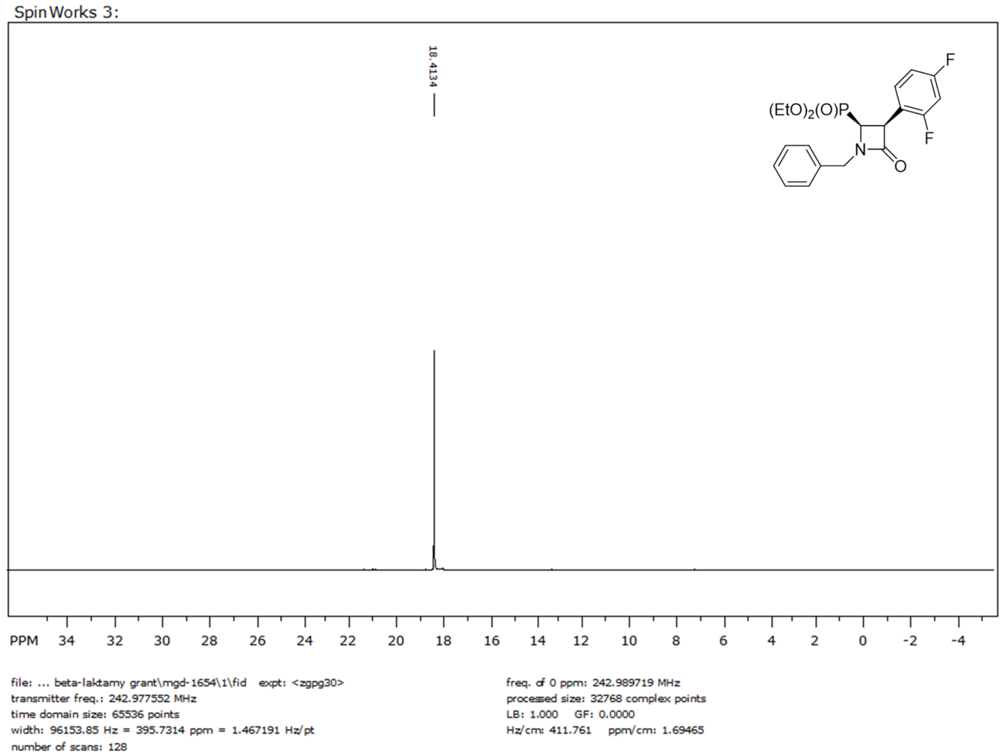

Figure S51: ^{31}P NMR Spectrum for *cis*-**11e** in $CDCl_3$

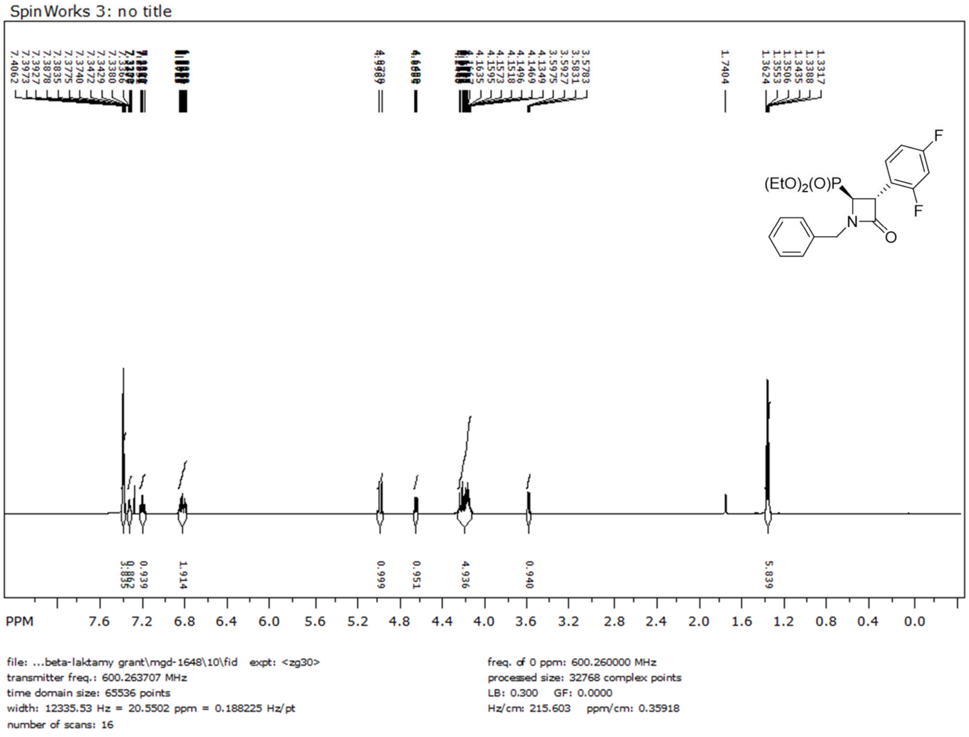

Figure S52: ^{1}H NMR Spectrum for *trans*-**11e** in $CDCl_3$

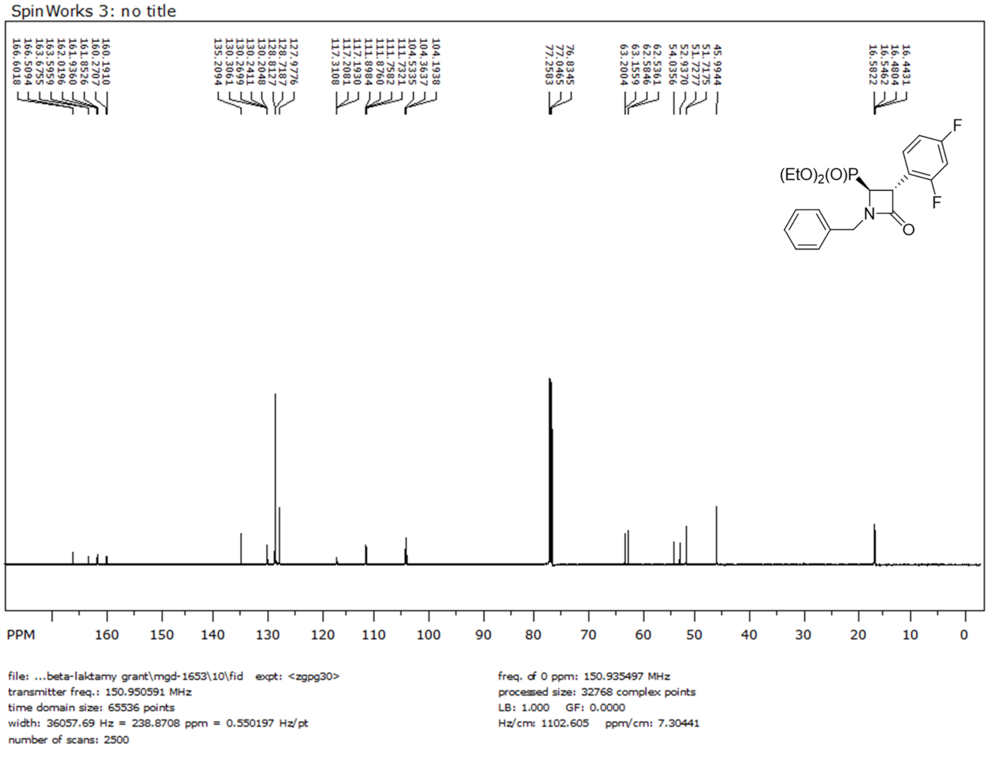

Figure S53: ^{13}C NMR Spectrum for *trans*-**11e** in $CDCl_3$

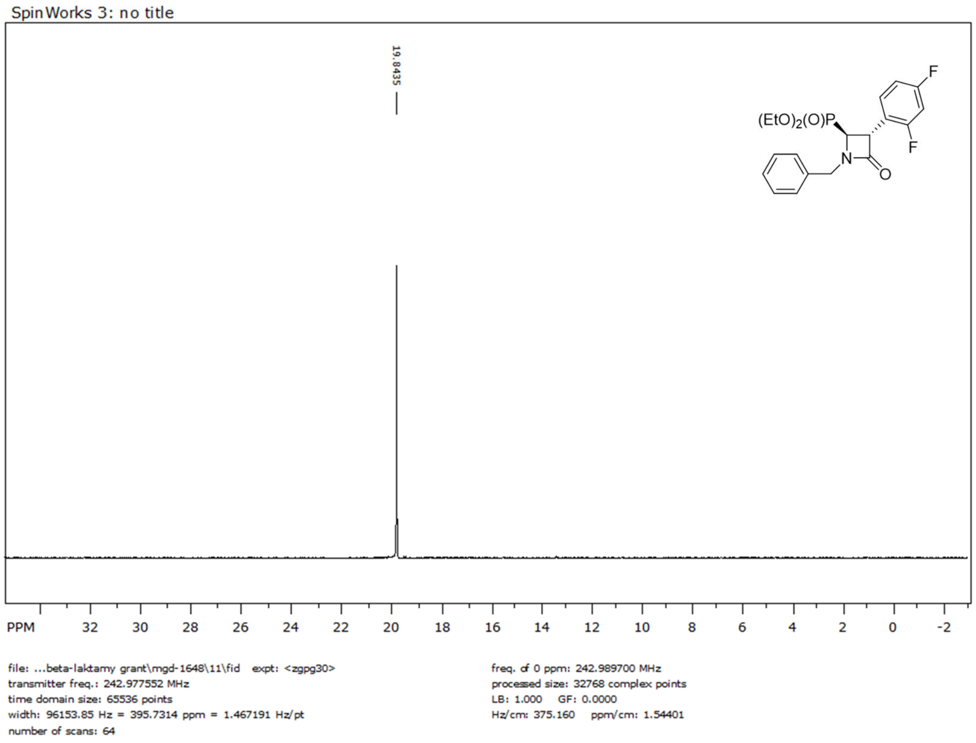

Figure S54: ^{31}P NMR Spectrum for *trans*-**11e** in $CDCl_3$

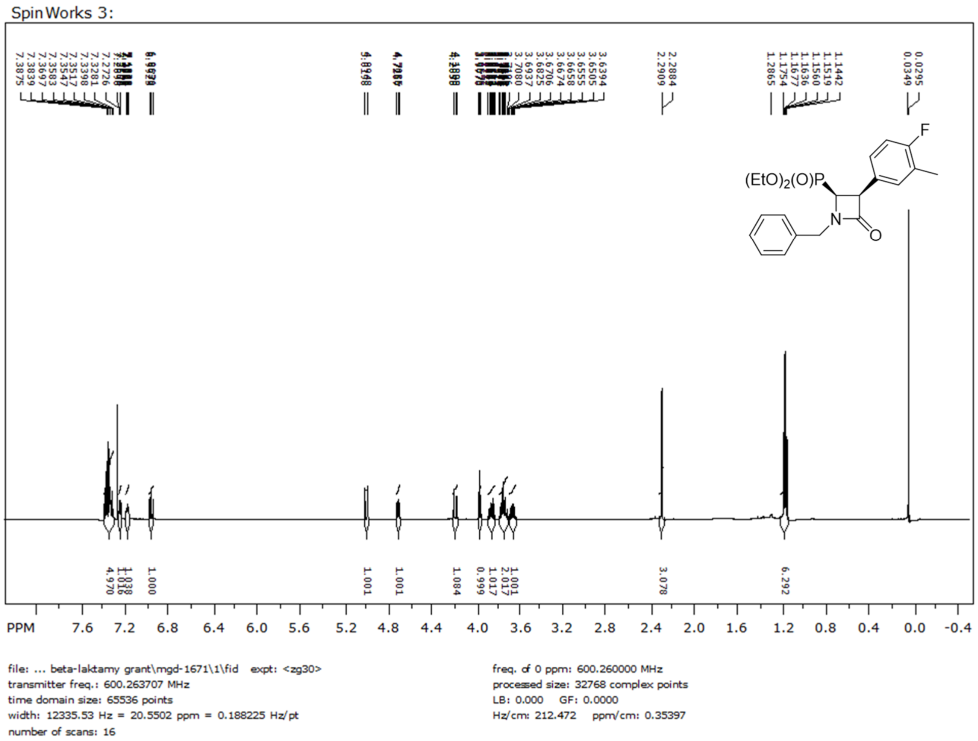

Figure S55: ^{1}H NMR Spectrum for *cis*-**11f** in $CDCl_3$

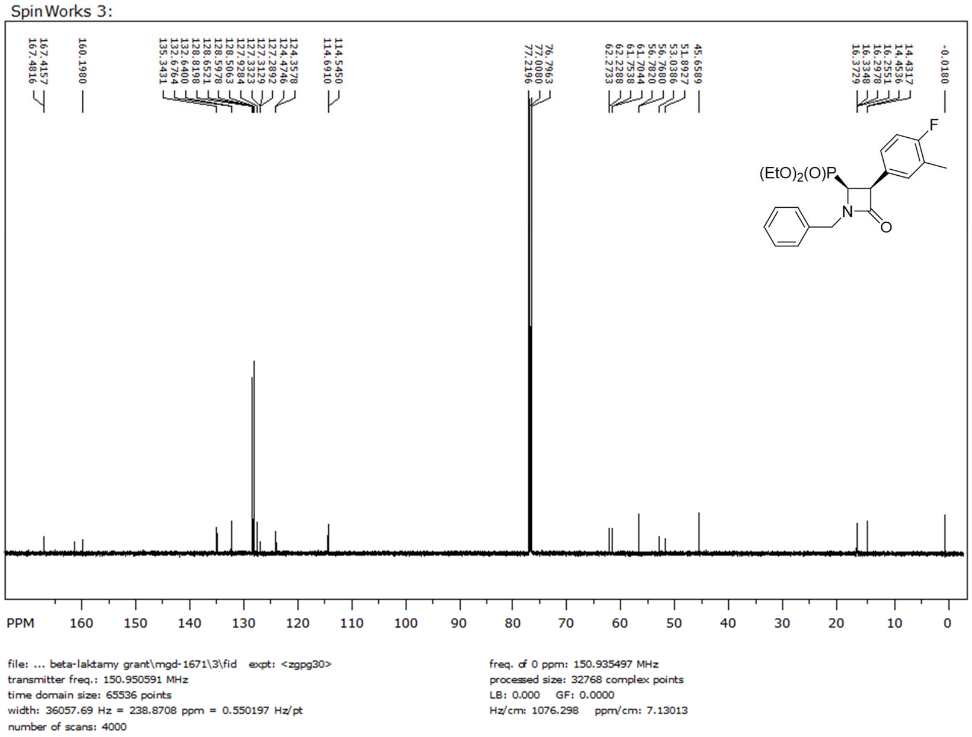

Figure S56: ^{13}C NMR Spectrum for *cis*-**11f** in $CDCl_3$

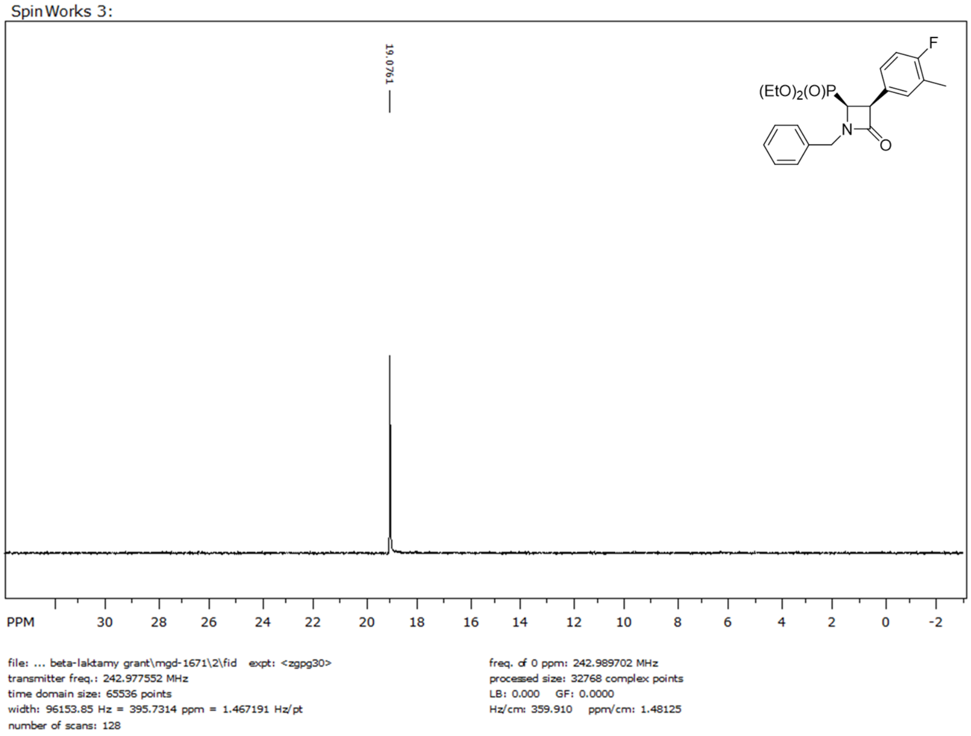

Figure S57: ^{31}P NMR Spectrum for *cis*-**11f** in $CDCl_3$

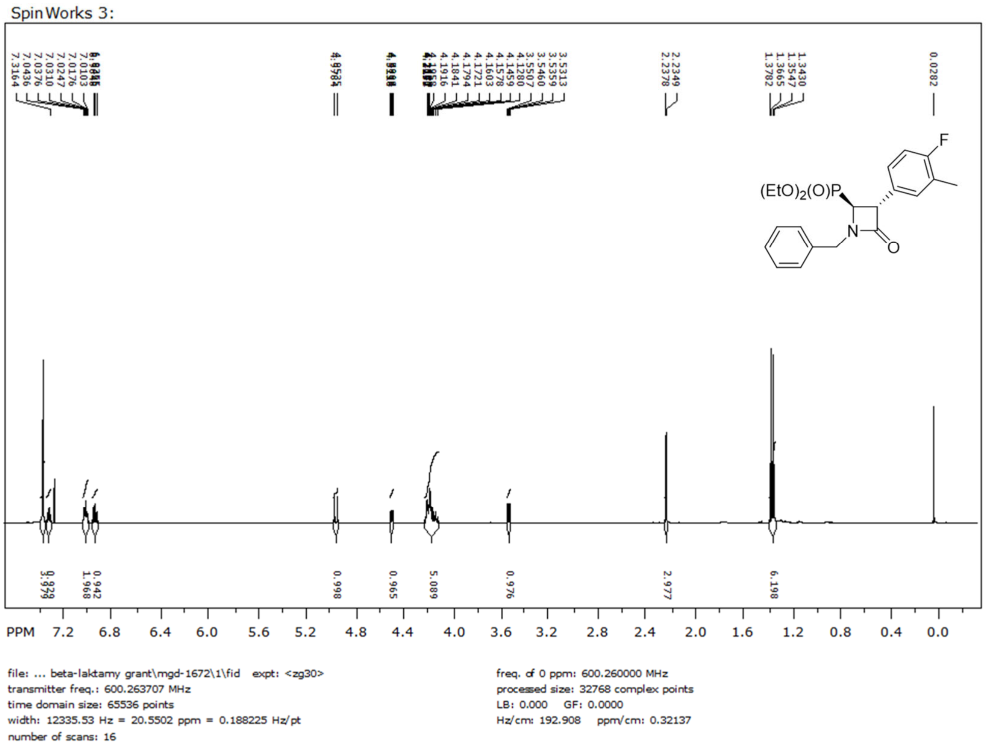

Figure S58: ^{1}H NMR Spectrum for *trans*-**11f** in $CDCl_3$

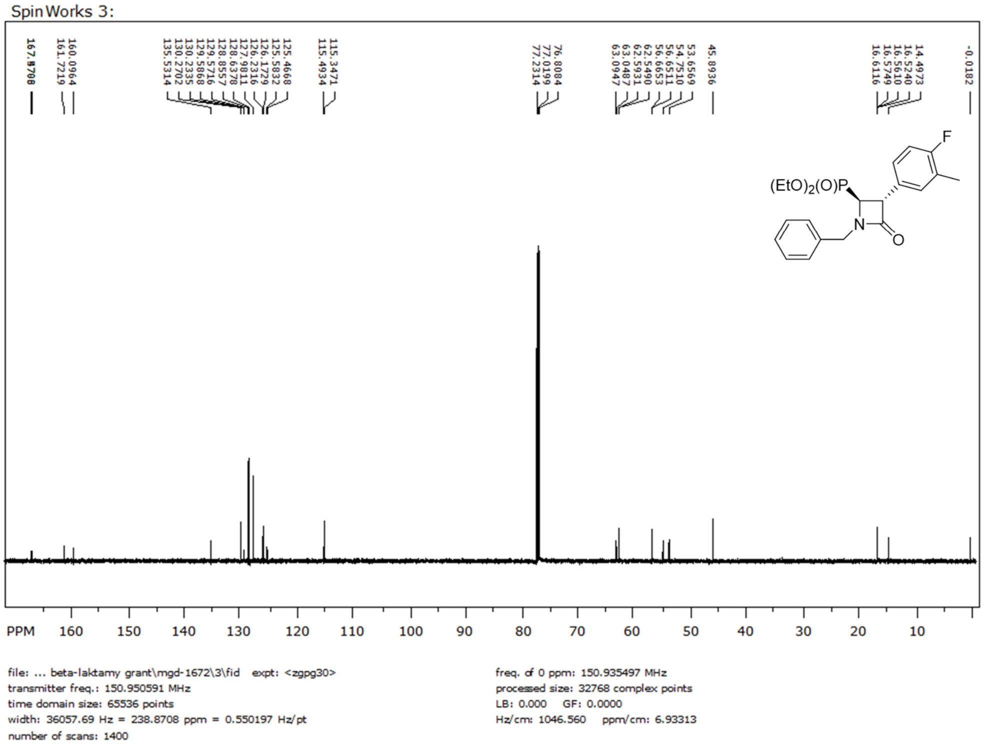

Figure S59: ^{13}C NMR Spectrum for *trans*-**11f** in $CDCl_3$

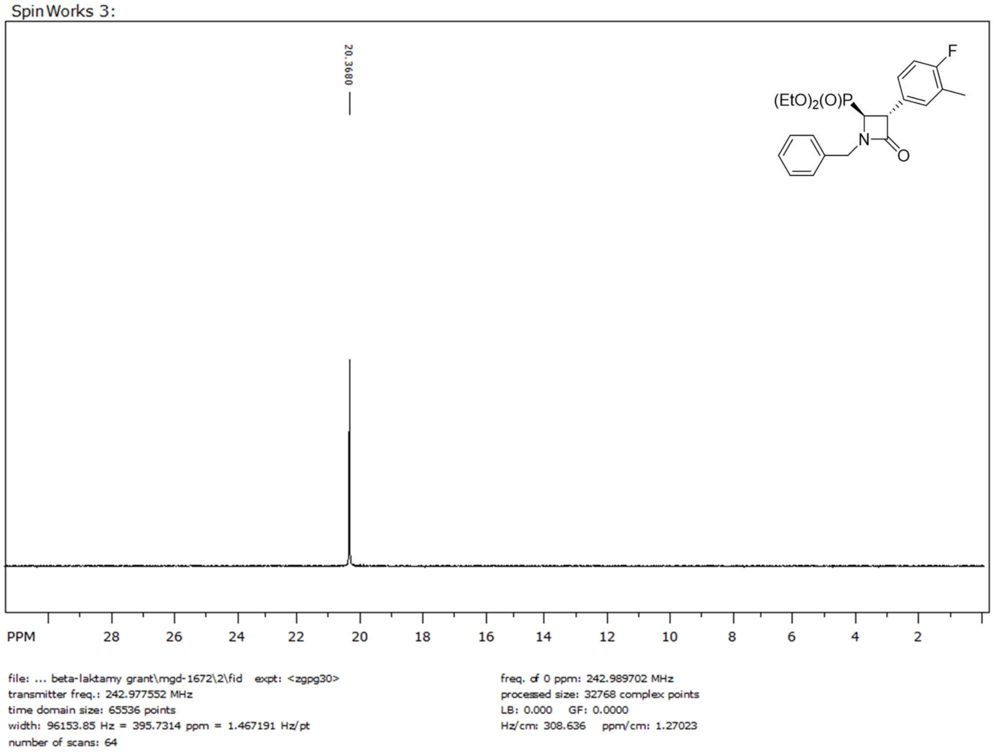

Figure S60: ^{31}P NMR Spectrum for *trans*-**11f** in $CDCl_3$
